# Reduced fire severity offers near-term buffer to climate-driven declines in conifer resilience across the western United States

**DOI:** 10.1073/pnas.2208120120

**Published:** 2023-03-06

**Authors:** Kimberley T. Davis, Marcos D. Robles, Kerry B. Kemp, Philip E. Higuera, Teresa Chapman, Kerry L. Metlen, Jamie L. Peeler, Kyle C. Rodman, Travis Woolley, Robert N. Addington, Brian J. Buma, C. Alina Cansler, Michael J. Case, Brandon M. Collins, Jonathan D. Coop, Solomon Z. Dobrowski, Nathan S. Gill, Collin Haffey, Lucas B. Harris, Brian J. Harvey, Ryan D. Haugo, Matthew D. Hurteau, Dominik Kulakowski, Caitlin E. Littlefield, Lisa A. McCauley, Nicholas Povak, Kristen L. Shive, Edward Smith, Jens T. Stevens, Camille S. Stevens-Rumann, Alan H. Taylor, Alan J. Tepley, Derek J. N. Young, Robert A. Andrus, Mike A. Battaglia, Julia K. Berkey, Sebastian U. Busby, Amanda R. Carlson, Marin E. Chambers, Erich Kyle Dodson, Daniel C. Donato, William M. Downing, Paula J. Fornwalt, Joshua S. Halofsky, Ashley Hoffman, Andrés Holz, Jose M. Iniguez, Meg A. Krawchuk, Mark R. Kreider, Andrew J. Larson, Garrett W. Meigs, John Paul Roccaforte, Monica T. Rother, Hugh Safford, Michael Schaedel, Jason S. Sibold, Megan P. Singleton, Monica G. Turner, Alexandra K. Urza, Kyra D. Clark-Wolf, Larissa Yocom, Joseph B. Fontaine, John L. Campbell

**Affiliations:** ^a^Department of Ecosystem and Conservation Sciences, University of Montana, Missoula, MT 59812; ^b^The Nature Conservancy, Tucson, AZ 85719; ^c^The Nature Conservancy, Portland, OR 97214; ^d^Region 6 Ecology Program, US Forest Service, Wenatchee, WA 98801; ^e^Monitoring, Evaluation, and Learning Program, Chief Conservation Office, The Nature Conservancy, Arlington, VA 22201; ^f^Ecological Restoration Institute, Northern Arizona University, Flagstaff, AZ 86011; ^g^The Nature Conservancy, Flagstaff, AZ 86001; ^h^The Nature Conservancy, Boulder, CO 80302; ^i^Department of Integrative Biology, University of Colorado–Denver, Denver, CO 80204; ^j^School of Environmental and Forest Sciences, University of Washington, Seattle, WA 98195; ^k^Department of Forest Management, University of Montana, Missoula, MT 59812; ^l^The Nature Conservancy, Seattle, WA 98121; ^m^Center for Fire Research and Outreach, University of California, Berkeley, CA 94720; ^n^School of Environment and Sustainability, Western Colorado University, Gunnison, CO 81231; ^o^Department of Natural Resources Management, Texas Tech University, Lubbock, TX 79409; ^p^New Mexico Forestry Division, Energy, Minerals, Natural Resources Division, Santa Fe, NM 87505; ^q^Department of Geography, The Pennsylvania State University, University Park, PA 16802; ^r^Department of Biology, University of New Mexico, Albuquerque, NM 87131; ^s^Graduate School of Geography, Clark University, Worcester, MA 01610; ^t^Conservation Science Partners, Truckee, CA 96161; ^u^US Forest Service, Pacific Southwest Research Station, Placerville, CA 95667-5199; ^v^The Nature Conservancy, Sacramento, CA 95811; ^w^Forest and Rangeland Stewardship Department, Colorado State University, Fort Collins, CO 80523; ^x^Colorado Forest Restoration Institute, Colorado State University, Fort Collins, CO 80523; ^y^Earth and Environmental Systems Institute, The Pennsylvania State University, University Park, PA 16802; ^z^Department of Forestry, Fire, and Rangeland Management, Cal Poly Humboldt University, Arcata, CA 95521; ^aa^Department of Plant Sciences, University of California, Davis, CA 95616; ^bb^School of Environment, Washington State University, Pullman, WA 99164; ^cc^Rocky Mountain Research Station, US Forest Service, Fort Collins, CO 80526; ^dd^Montana Department of Natural Resources and Conservation, Missoula, MT 59806; ^ee^Department of Geography, Portland State University, Portland, OR 97207; ^ff^Department of Forest and Wildlife Ecology, University of Wisconsin–Madison, Madison, WI 53706; ^gg^Rocky Mountain Research Station, US Forest Service, Ogden, UT 84401; ^hh^Washington State Department of Natural Resources, Olympia, WA 98504; ^ii^Department of Forest Ecosystems and Society, College of Forestry, Oregon State University, Corvallis, OR 97331; ^jj^Rocky Mountain Research Station, USDA Forest Service, Flagstaff, AZ 86001; ^kk^Wilderness Institute, University of Montana, Missoula, MT 59812; ^ll^Department of Environmental Sciences, University of North Carolina Wilmington, Wilmington, NC 28403; ^mm^Vibrant Planet, Incline Village, NV 89451; ^nn^Department of Environmental Science and Policy, University of California, Davis, CA 95616; ^oo^The Nature Conservancy, Missoula, MT 59802; ^pp^Department of Anthropology and Geography, Colorado State University, Fort Collins, CO 80523; ^qq^Graduate Degree Program in Ecology, Colorado State University, Fort Collins, CO 80523; ^rr^School of Forestry, Northern Arizona University, Flagstaff, AZ 86011; ^ss^Department of Integrative Biology, University of Wisconsin–Madison, Madison, WI 53706; ^tt^Rocky Mountain Research Station, USDA Forest Service, Reno, NV 89512; ^uu^Department of Wildland Resources and the Ecology Center, Utah State University, Logan, UT 84322; ^vv^Environmental and Conservation Sciences, Murdoch University, Perth, WA 6150, Australia

**Keywords:** climate change, wildfire, ecological transformation, post-fire regeneration, vegetation transition

## Abstract

Increasing fire severity and warmer, drier postfire conditions are making forests in the western United States (West) vulnerable to ecological transformation. Yet, the relative importance of and interactions between these drivers of forest change remain unresolved, particularly over upcoming decades. Here, we assess how the interactive impacts of changing climate and wildfire activity influenced conifer regeneration after 334 wildfires, using a dataset of postfire conifer regeneration from 10,230 field plots. Our findings highlight declining regeneration capacity across the West over the past four decades for the eight dominant conifer species studied. Postfire regeneration is sensitive to high-severity fire, which limits seed availability, and postfire climate, which influences seedling establishment. In the near-term, projected differences in recruitment probability between low- and high-severity fire scenarios were larger than projected climate change impacts for most species, suggesting that reductions in fire severity, and resultant impacts on seed availability, could partially offset expected climate-driven declines in postfire regeneration. Across 40 to 42% of the study area, we project postfire conifer regeneration to be likely following low-severity but not high-severity fire under future climate scenarios (2031 to 2050). However, increasingly warm, dry climate conditions are projected to eventually outweigh the influence of fire severity and seed availability. The percent of the study area considered unlikely to experience conifer regeneration, regardless of fire severity, increased from 5% in 1981 to 2000 to 26 to 31% by mid-century, highlighting a limited time window over which management actions that reduce fire severity may effectively support postfire conifer regeneration.

## Significance

Wildfires in the western United States are concerning in part because conifer forests may not regenerate under increasingly warm, dry climate conditions and severe burning. This study compared the relative importance of differences in fire-caused tree mortality, which limits seeds available for tree regeneration, to the impacts of warm, dry climate conditions in determining postfire conifer regeneration. Using observations from over 10,000 sites, we found that warmer, drier conditions are leading to less tree regeneration after wildfires. We also found evidence that management interventions that reduce wildfire severity can partially offset these climate-related declines in tree regeneration. Our work highlights the next few decades as a window of opportunity over which management could minimize the likelihood of fire-caused forest loss.

Ecological transformation due to climate change is a global phenomenon with significant socioecological impacts, including changes to carbon storage, water quality, biodiversity, and culturally important resources (1–3). Forests globally are increasingly vulnerable to ecological transformation due to changing climatic conditions that simultaneously increase wildfire activity (4–7) and alter key postfire demographic rates such as seedling establishment (8, 9), a phenomenon broadly termed “interval squeeze” (10). Declines in tree recruitment have been observed globally, causing widespread concerns about forest loss following wildfires and other disturbances (1, 3, 11–13).

Conifer forests of the western United States (West) may be especially vulnerable to ecological transformation because climate change impacts are compounded by more than a century of wildfire suppression, exclusion of indigenous burning, logging of large fire-resistant trees, and other forest management practices (14, 15). Together, these changes have fundamentally altered forest structure, composition, and fire regimes and are leading to more severe fire effects in many forests that historically experienced low- and moderate-severity fire (14). High-severity fire sets the stage for ecological transformation by killing mature trees, altering microclimate (16) and soil properties (17), and reducing seed sources on the landscape. Even in forests that historically burned in stand-replacing fires, recent changes in fire frequency and postfire climate may significantly alter vegetation trajectories following high-severity fire (3, 12, 18–20).

The combination of changes in fire regimes and climate is driving declines in postfire conifer regeneration across the West (21–24). Yet, despite the abundance of postfire tree regeneration data that have been collected in the West, the relative importance, interactions, and feedbacks between these two drivers of conifer regeneration are poorly resolved at the regional scale. This knowledge is crucial for identifying vulnerability to ecological transformation and key opportunities for potential management interventions. For instance, local management actions can readily and rapidly drive changes in fuel and forest structure, an important driver of fire severity, but have limited immediate impact on macroscale climate conditions. There is substantial evidence that fuel reduction treatments, especially those using prescribed burning, effectively reduce local wildfire severity in dry forests (25, 26). Significant increases in government investment in these types of treatments in the United States, including $3 billion dedicated to a 10-y strategy to reduce fuels across 20 million ha (27, 28), highlight the need for spatial prioritization of treatments.

Forward-looking management approaches, such as the Resist-Accept-Direct (RAD) framework (2, 29), also require quantitative information on the probability of fire-driven ecological transformation. The RAD framework, which shares similarities with previous frameworks (30, 31), helps managers make informed, deliberate choices about the trajectory of change when managing systems undergoing rapid ecological transformation. Effectively prioritizing where to resist, accept, or direct postfire vegetation trajectories requires an understanding not only of the likelihood of postfire ecological transformation, but also of where reducing fire severity through management interventions could change this likelihood (32, 33), and when and where climate conditions have crossed critical thresholds that may preclude forest recovery (9), regardless of fire severity.

**Fig. 1.**
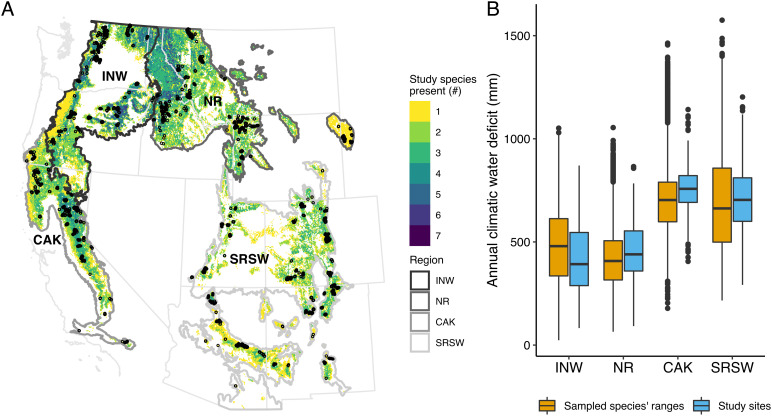
Characteristics of the 10,230 plots utilized in this study. (*A*) Four study regions (gray outlines), study sites from which postfire tree regeneration was sampled (black points), and forest area that contains at least one of the eight study species within each region (colored by number of study species), hereafter “study area”. The four study regions (INW: interior Northwest; NR: northern Rockies; CAK: California and the Klamath; SRSW: southern Rockies and AZ/NM mountains) were defined by aggregating level 3 US Environmental Protection Agency ecoregions that contained field sites. Across the study area (*A*), the eight study species account for 89% of the conifer basal area (based on values from ref. 34). (*B*) 30-y mean annual climatic water deficit (1981 to 2010) of the study area compared with that of the sampled study sites for each region.

Here, we resolve how the interactive impacts of changing climate and fire severity have influenced postfire conifer regeneration across western US forests. We use a dataset of postfire conifer regeneration from 10,230 field plots that represents 334 fires that occurred between 1984 and 2018 (Fig. 1 and Dataset S1) and spans the climatic range of eight widespread conifer species across the West (white fir, *Abies concolor*; grand fir, *Abies grandis;* subalpine fir, *Abies lasiocarpa*; Engelmann spruce, *Picea engelmannii*; lodgepole pine, *Pinus contorta*; Jeffrey pine, *Pinus jeffreyi*; ponderosa pine, *Pinus ponderosa*; and Douglas-fir, *Pseudotsuga menziesii*). Together, these species represent 89% of the conifer basal area in the study area [Fig. 1*A*; (34)]. All species are obligate seeders. The interior subspecies of *P. contorta* (spp. *latifolia*) can be serotinous and regenerate prolifically following high-severity fire, although levels of serotiny vary widely among individuals and populations (35). We used these field data to model postfire recruitment probability for each species individually and for all species combined as a function of biophysical predictors representing seed availability, fire severity, 30-y mean climate, and postfire climate (*SI Appendix*, Table S1). Models for *P. ponderosa* and *P. jeffreyi* and for *A. concolor* and *A. grandis* were combined (see *Methods*) for a total of six species models. These models reveal key drivers of postfire conifer regeneration across western US conifer forests. We use these models to project postfire recruitment probability under multiple scenarios of fire severity (low- and high-severity) and climate change (2031 to 2050; Representative Concentration Pathway (RCP) 4.5 and 8.5; *SI Appendix*, Tables S2 and S4). We are thus able to: 1) compare the relative impacts of changes in climate and fire severity on postfire conifer regeneration across the West; 2) assess how these impacts vary by region and species; and 3) identify when and where climate change is likely to cause postfire seedling recruitment failure and resulting ecological transformation. We discuss the implications of our results for forest management using the RAD framework.

## Results

### Climate Change Will Reduce the Probability of Postfire Tree Regeneration.

**Fig. 2.**
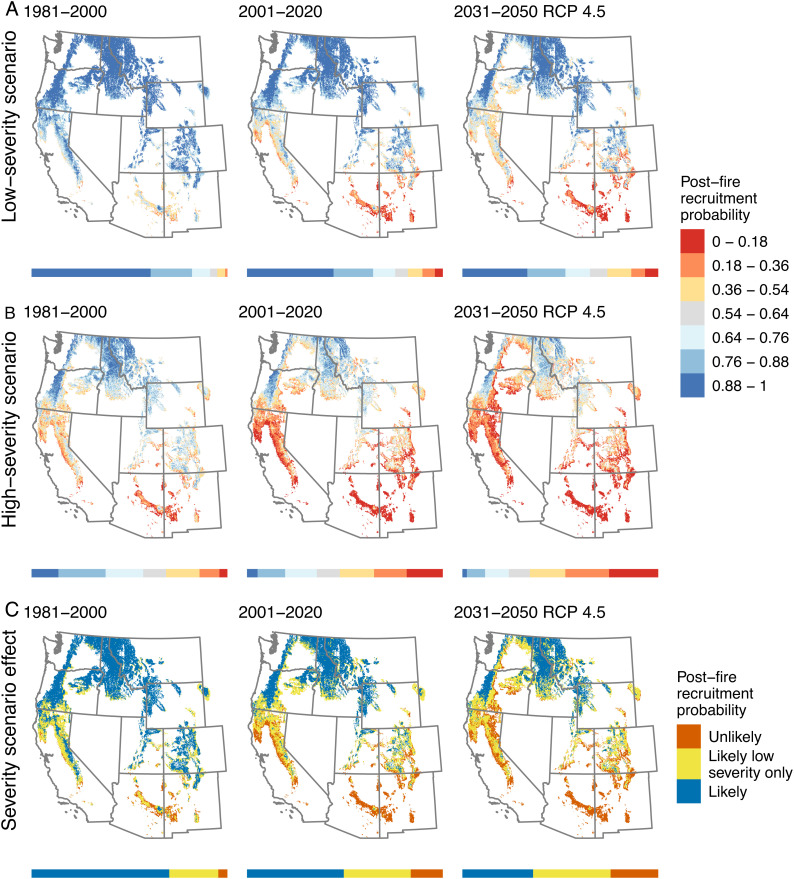
Regional variability in postfire conifer recruitment under past and future climate and fire-severity scenarios. Postfire conifer recruitment probability from the all-species model under past and current climate, a future climate scenario (Representative Concentration Pathway (RCP) 4.5), and (*A*) low- and (*B*) high-severity fire scenarios. Bars beneath maps show the proportion of the study area that falls into each category. Shades of blue represent areas where recruitment is likely, whereas warm colors represent areas where recruitment is unlikely. Areas in gray highlight the range of threshold probabilities above which recruitment is likely (see *Methods*). (*C*) Differences in recruitment probability between fire severity scenarios. Map shows where recruitment is unlikely under both fire-severity scenarios (orange), likely under only the low severity scenario (yellow), or likely under both severity scenarios (blue; *SI Appendix*, Table S18). Results for future climate under the RCP 8.5 scenario shown in *SI Appendix*, Fig. S10.

Our findings highlight intensifying losses of dominant conifer species regeneration capacity across the western United States. The likelihood of postfire regeneration for all species declined under future climate scenarios across the study area (Fig. 2). For example, the percent of the study area considered likely to experience regeneration (recruitment probability > 0.54) by 10 y postfire decreased from 95% in 1981 to 2000 to 74% by 2031 to 2050 under RCP 4.5 in projections from the all-species model.

**Fig. 3.**
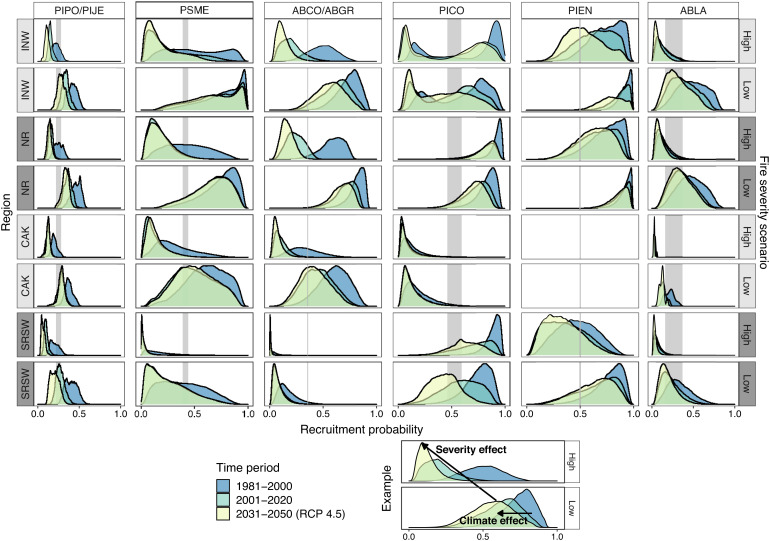
Changing postfire tree recruitment under past and future climate and fire-severity scenarios. Distribution of recruitment probability projected by individual species models across the range of each species within each region (see Fig. 1 caption for region abbreviations). Species columns are ordered from lower elevation species (*Left*) to higher elevation species (*Right*). Different colors represent the different time periods. Rows represent fire-severity scenario and region combinations. The gray vertical band highlights the range of threshold probabilities above which recruitment is likely (see *Methods*). “PIPO/PIJE” is *P. ponderosa/P. jeffreyi*; “PSME” is *P. menziesii*; “ABCO/ABGR” is *A. concolor/A. grandis*; “PICO” is *P. contorta*; “PIEN” is *P. engelmannii*; “ABLA” is *A. lasiocarpa*.

Importantly, spatial and temporal patterns of change in recruitment probability varied by species and region (Fig. 3 and *SI Appendix*, Figs. S15–S26). Lower elevation species (*P. ponderosa, P. jeffreyi*, *P. menziesii*, *A. concolor*, and *A. grandis*) have already experienced a significant decline in recruitment probability between the 1981 to 2000 and the 2001 to 2020 time periods, while higher elevation species such as *P. contorta* and *P. engelmannii* are predicted to experience more declines in the coming decades in some regions (Fig. 3). Likewise, forests in the California and Klamath (CAK) and southern Rockies and AZ/NM mountain (SRSW) regions (Fig. 1*A*) saw significant declines in recruitment probability over the same time period, and we project similar patterns to expand northward by 2050, especially in drier forests of the interior Northwest (INW; Figs. 2 and 3). However, wetter and cooler portions of the INW and much of the northern Rockies (NR) were projected to remain climatically suitable for postfire regeneration of the study species through mid-century. Although there was some variability in future projections among global climate models (GCMs), the general patterns were consistent (*SI Appendix*, Figs. S13 and S14).

### Reductions in Fire Severity Can Ameliorate Impacts of Climate Change.

Our results suggest reductions in overall fire severity or the size of high-severity patches could partially offset expected declines in postfire regeneration attributed to climate change alone. In our projections across the study area, changes in fire severity, which included changes to seed availability, had a greater relative effect on recruitment probabilities than did changing climate conditions for most species, with the exception of *P. contorta*, which due to its serotinous cones can regenerate prolifically following high severity fire in some areas (Figs. 2 and 3 and *SI Appendix*, Figs. S9 and S15–S26). For example, when we projected recruitment across the study area for all species under climate and fire severity scenarios, median recruitment probability decreased by an average of 0.34 with a change from low to high fire severity, as opposed to a decrease of only 0.12 on average due to climate change in successive time periods (*SI Appendix*, Table S17). In a substantial portion of the study area (26 to 42%, 17 to 28 million ha, depending on time period and future climate scenario; *SI Appendix*, Table S18), postfire conifer regeneration was likely (probability > 0.54) in the low-severity scenario but unlikely in the high-severity scenario (Fig. 2*C*). The effect of fire severity was most pronounced in the NR and least pronounced for dry forest species like *P. ponderosa* and *P. menziesii* in the SRSW, where climate and fire severity had similar impacts (Figs. 2 and 3 and *SI Appendix*, Fig. S9).

**Fig. 4.**
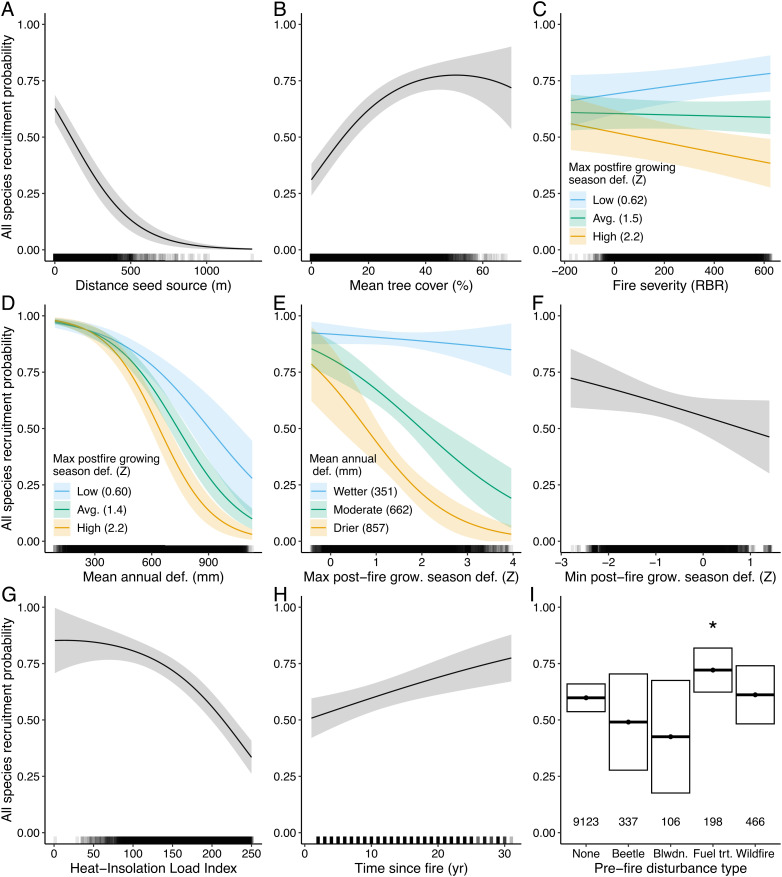
Climatic and nonclimatic controls of postfire tree regeneration. Partial dependence plots for the all-species model showing the relationship between model predictors and postfire recruitment probability while holding other variables at their median values. Mean annual def. is the 30-y mean climatic water deficit from 1981 to 2010. “Max. (Min.) postfire growing season def.” is the maximum (minimum) growing season (April to September) climatic water deficit anomaly experienced in the first 5 y postfire. Where interactions were significant, they are shown by plotting blue, green, and orange lines for the 10th, 50th, and 90th percentiles, respectively, of the interacting variables from our dataset. Bands in *A*–*H* and boxes in *I* are 95% CIs. “*” indicates significantly different (*P* < 0.05) than no prefire disturbance. Rug plot on the *x*-axis in *A*–*H* show the distribution of data. Numbers above *x*-axis in *I* show sample size for each group. Partial dependence plots for individual species models shown in *SI Appendix*, Figs. S2–S7.

Fire severity interacted significantly with climate such that high fire severity exacerbated the impacts of warm, dry postfire climate for several species and in the all-species model (Fig. 4*C* and *SI Appendix*, Figs. S2–S4). For example, for both *P. ponderosa* and *P. menziesii*, the reduction in recruitment probability associated with high climatic water deficit (“water deficit”) following fire was stronger in areas that burned at high severity than in areas that burned at low severity (*SI Appendix*, Figs. S2 and S3). For the true fir species (*Abies* spp.), we found similar interactions between postfire climate and surrounding tree cover (within 300 m), which is correlated with the proportion of the area surrounding a plot that burned at high severity (*SI Appendix*, Figs. S4–S7). In areas with more remaining live tree cover postfire, the effects of warm, dry postfire climate were ameliorated. Under future climate conditions, these interactions led to greater projected declines in recruitment probability in the high- than low-severity scenario for some species (e.g., Fig. 3, *A. concolor/A. grandis* and *P. menziesii*; *SI Appendix*, Figs. S18 and S20).

### Seed Availability and Moisture Deficits Control Postfire Conifer Regeneration.

Postfire recruitment probability was strongly related to metrics of seed availability and fire severity (Fig. 4 and *SI Appendix*, Figs. S2–S7 and Tables S9–S15). For each species, recruitment declined at farther distances from the nearest seed source and when surrounding tree cover was lower—indicating fewer living trees within plausible dispersal distances to provide seeds—a situation that may arise in areas with larger high-severity patches (36). The effect of satellite-derived fire severity metrics varied by species, with ecologically coherent patterns: recruitment probability declined at higher fire severities with relatively shade-tolerant *P. menziesii* and true firs (*Abies* spp.); in contrast, after accounting for seed availability, recruitment probability increased with higher fire severity for the Rocky Mountain varieties of the shade-intolerant *P. contorta* and *P. ponderosa* (*SI Appendix*, Figs. S2–S7). Notably, Rocky Mountain *P. contorta* (spp. *latifolia*) exhibits cone serotiny, which facilitates recruitment in areas burned at high severity (35).

Postfire conifer regeneration also strongly depended on both average climate conditions and postfire climate anomalies. In both cases, climate metrics reflecting water availability to trees were the most influential in statistical models. Considering all-species combined, seedlings were more sensitive to postfire climate conditions when they were present in warm, dry sites, as indicated by higher mean water deficit (Fig. 4*E*). Additionally, warmer, drier sites also had lower postfire recruitment probabilities overall (Fig. 4*D* and *SI Appendix*, Figs. S2–S7). A single hot, dry year (high growing season water deficit) within the first 5 y following a fire significantly reduced the probability of postfire regeneration for most species (i.e., *P. ponderosa/P. jeffreyi*, *P. contorta*, *A. lasiocarpa*, *P. menziesii*) and when considering all species combined (Fig. 4*E* and *SI Appendix*, Figs. S2–S7). Each species except subalpine fir also experienced an increase in recruitment probability if there was at least one unusually cool, wet year following fire (Fig. 4*F* and *SI Appendix*, Figs. S2–S7), represented by high growing season precipitation (*P. ponderosa/P. jeffreyi*, *P. contorta*), low growing season water deficit (*P. engelmannii*, *P. menziesii*), or low summer vapor pressure deficit (*A. concolor/A. grandis*).

## Discussion

Our analyses shed light on the interactive impacts of changing climate and fire severity on past and likely future postfire conifer regeneration across a broad region of the West. Climate change over the past four decades has already led to significant reductions in the probability of conifer regeneration after wildfires across this region, and we project that climate will increasingly limit postfire tree recruitment in the future, consistent with the interval squeeze phenomenon. Consequently, we expect postfire ecological transformation will become more likely, underscoring the importance of the Resist-Accept-Direct (RAD) framework for informing prefire and postfire management decisions (37). Equally important, however, we also project continued successful conifer regeneration in many areas, especially the northern Rockies and higher elevation forests.

Despite the importance of climate for postfire conifer recruitment, we found that fire severity had a larger relative impact on projected recruitment probability than the direct effects of near-term climate change for most species studied here. Fire severity impacted recruitment probability directly via impacts to seed supply and indirectly by exacerbating the effects of dry postfire climate, potentially through changes to microclimate (16), soil properties (17) or competing vegetation. Changes to fire regimes resulting from fire suppression policies, past management practices, or climate change that have increased the likelihood of high fire severity and increased high-severity patch sizes (5, 6, 14, 38) may be playing a larger role driving reductions in postfire conifer regeneration than direct climate impacts alone. Our work resolves key ecological uncertainties across a broad spatial extent and highlights significant opportunities to influence postfire regeneration and resist ecological transformation through management to reduce fire severity in some forests (26, 32).

While reduced fire severity and subsequent increases in seed availability can partly ameliorate the impacts of increasing moisture deficits on postfire conifer regeneration, there are important limitations to this tradeoff. First, a warming climate has been associated with more area burning at high severity in recent decades (5, 6). Thus, climate change impacts postfire recruitment directly by creating warmer, drier conditions after fire and indirectly through an increase in high-severity fire. Second, our work highlights the critical importance of understanding climate thresholds for recruitment (9, 18). During years when climate exceeds the moisture deficit thresholds that can support postfire conifer germination and seedling survival, regeneration is unlikely regardless of fire severity. The more pronounced effect of postfire climate at dry sites (Fig. 4*E*) suggests that as average climate conditions become warmer and drier, these thresholds limiting conifer recruitment will be crossed more frequently (9, 39). Importantly, experiencing just 1 y with a high moisture deficit within the first 5 y following fire significantly decreased the likelihood of postfire conifer recruitment. Combined, these limitations suggest that efforts to resist loss of western US forests via reductions in fire severity may only be effective during a relatively short period over the upcoming decades, a window of opportunity that varies by ecoregion and forest type. Managers and decision makers may thus wish to prioritize interventions in forests most vulnerable to fire-driven forest loss (e.g., dry forests of the Southwest and California) where this window is expected to close within the next few decades.

By identifying where and when fire-driven transformation is likely, our near-term projections of recruitment probability can inform management choices to resist, accept, or direct postfire vegetation transitions when applying the RAD framework (2, 29). These decisions are highly context-specific, informed by potential future conditions, local management goals, and integrating social-ecological factors beyond this work (29, 33, 40, 41). Nonetheless, our results provide insight into the tradeoffs between these choices. For example, in areas of severe climate limitations on recruitment of current species, management to resist change will only delay change and it may be prudent to consider how to direct change in those areas; however, in areas of continued climate suitability investment in resisting change may yield longer-term results.

Decision constraints and opportunities vary throughout conifer forests of the western United States depending on forest type, historical land use patterns, and historical fire regimes (15). In lower elevation dry conifer forests that historically experienced frequent fire, our results highlight the potential to resist fire-driven transformations from forest to nonforest through management activities that effectively reduce fire severity [e.g., treatments including forest thinning and reduction of surface fuels with prescribed or cultural fire (25, 26)]. Identifying areas where a reduction in fire severity will have the highest potential to mitigate postfire conifer regeneration failure (Fig. 2*C*) may help to prioritize locations for management activities. Despite the potential benefits of effective management interventions, wildfire affects much more area than management actions may be able to feasibly treat (42), highlighting the need to proactively integrate managed wildfire with other strategies to reduce fuels and enhance ecosystem resilience across large landscapes (25, 43). Where climate is already unsuitable for conifer regeneration, managers may decide to accept transitions to other vegetation types where key management goals can still be met, vital ecosystem services are sustained or stabilized, or nonconifer or early-successional habitat (e.g., oak woodlands, shrublands) are established in areas where these habitat types are limited compared with their historical extent (14, 15, 44). In other cases, historically unprecedented large high-severity patches dominated by nonforest vegetation types may homogenize landscape patterns and/or lead to novel species assemblages (5, 45, 46). Where accepting change would be undesirable from a societal or management perspective, managers may consider directing change toward more desirable conditions, for example by planting drought and fire-resistant species or genotypes (including *Populus tremuloides* or *Quercus* spp. that resprout following fire) or planting at lower densities (33, 40, 47–49). Given significant uncertainty about the longer term impacts of directing change (40, 49, 50), adaptive management and monitoring approaches will be particularly important (51).

Subalpine conifer forests in our study area have been resilient to high-severity fire for millennia (e.g., refs. 52 and 53). However, our results suggest that changes to climate (Fig. 3), combined with changes to forest structure (14) and the frequency of burning (19, 54, 55), have altered the conditions that made these forests historically resilient to high-severity burning, even in contemporary forests where serotinous *P. contorta* is present. Postfire forest recovery in these systems occurs over decades to centuries (52), and continues to be likely in much of the northern Rockies (Figs. 3 and 4 and *SI Appendix*, Figs. S21–S26); however, in some areas such as the southern Rockies, the suitable climate window for regeneration is rapidly closing. Where climate is currently conducive to conifer establishment but will become unsuitable in the coming decades, managers may be able to resist changes in the short-term by postfire planting or seeding (33). Longer term resistance may be challenging in these forest types given the historical high-severity fire regimes and increasing potential for short-interval fire. Thus, as in lower elevation forests, periods immediately after fire may also provide opportunities to direct change toward species or genotypes that are better adapted to future climate conditions and fire regimes (33, 56). Where we project low recruitment probability, in both subalpine and lower elevation forests, the relatively coarse scale of our climate data (4-km resolution) may underestimate the role that local site factors, microclimate, and disturbance refugia (57–59) play in supporting postfire tree regeneration and forest persistence (60); thus identifying and protecting these refugia will be critical if the goal is to retain conifer forests (33, 40).

Our work highlights several key drivers of postfire conifer regeneration, but the broadscale nature of our study means that we could not account for local and microsite conditions, fine-scale weather events, intraspecific variation, cone serotiny, or phenotypic plasticity. For example, competition or facilitation from shrubs (23, 61), seed dispersal and predation (36, 62), interannual variation in seed production (63), pathogens, herbivory, or edaphic factors not captured in our models may affect conifer establishment and likely vary across our study area. Short-term exposure to extreme conditions [e.g., high soil surface temperatures (64)] can also cause seedling mortality. Additionally, the level of serotiny in *P. contorta* stands strongly impacts forest development after high severity fire (24, 35). We stress that such fine-scale patterns and processes are essential to regeneration dynamics and should be explicitly considered in developing site-specific management strategies (33, 40). Furthermore, while we project reduced recruitment potential within the range of the study species, in some cases fire may provide an opportunity for tree species to establish beyond their current range (e.g., ref. 65). Finally, by combining data from multiple studies with different field methods, we also introduce additional uncertainty.

We project that a substantial portion of the forests in our study region will experience declines in postfire conifer regeneration, which would have major implications for ecosystem structure and function. These results highlight the need to better understand what type of ecosystems will replace these forests when regeneration fails—likely to vary greatly by region—and the implications for carbon sequestration, hydrology, wildlife habitat, and other key ecosystem services on which society depends. Despite the pronounced impact of climate change, the stark contrast in the projections of conifer recruitment probability from the low- and high-severity scenarios emphasize how management actions taken to reduce fire severity can significantly shape postfire vegetation trajectories. Identifying whether, when, and where management intervention is appropriate to resist or direct trajectories of change in these forests will become more critical as wildfire affects more of the landscape each year (4, 29, 41). Importantly, by elucidating the interactive effects of climate and fire severity, we show that windows of opportunity for management intervention may decline as climate increasingly limits conifer recruitment in the near-term future.

## Methods

### Data Collection.

Existing datasets were solicited from collaborators across the western United States that met several criteria: 1) Plots were located in conifer-dominated forests of the western Unites States; 2) exact plot location and area were available; 3) data included presence of postfire conifer juveniles by species; 4) sampling occurred at a minimum of 2 y after wildfire (not prescribed fire) occurrence; and 5) surveyed wildfires occurred between 1984 and 2018. Control plots that were unburned or plots that were planted following fire were removed from the aggregated dataset, for a total of 10,230 plots (Dataset S1).

### Predictors.

Biophysical predictors for each site included a heat load index, fire severity, tree cover, and climate data (*SI Appendix*, Table S1). For consistency, we omitted predictors from our analysis that were not present in all datasets (e.g., prefire stand structure, serotiny). To represent the effects of insolation and topographic shading on seedling recruitment, we used a 90-m continuous heat-insolation load index (CHILI; (66)). To represent fire severity, we used a mean compositing approach in Google Earth Engine (67) to calculate the relativized burn ratio (RBR) with a phenology offset (30-m resolution).

We used two metrics to represent seed source availability. First, to represent the overall abundance of live trees (surrounding tree cover), we used the percent tree cover in a 300-m radius around each plot derived from postfire imagery from the Rangeland Analysis Platform [30-m resolution; (68)]. Second, we used distance to nearest live seed source. Data from 78% of plots included field measured distance to seed source, defined as distance to the nearest live reproductive tree(s). For plots without field measured distance to seed source, we manually recorded distance to the nearest live tree using 1-m aerial postfire imagery (National Agriculture Imagery Program) from the closest available year to the field sampling date in Google Earth Engine (*SI Appendix*, *Methods*). We did not represent the level of *P. contorta* serotiny in our models, due to a lack of available information on serotiny across the study area.

We extracted 4-km resolution daily climate data for 1979 to 2020 for each plot from gridMET (69). We developed metrics from these daily climate data to represent 30-y climatologies and 5-y postfire seasonal conditions. We used monthly averages to run a water balance model following Dobrowski et al. (70) and Rodman et al. (22) to calculate monthly climatic water deficit (potential evapotranspiration minus actual evapotranspiration) from 1979 to 2020. We then calculated 30-y mean annual and growing season (April to September) water deficit from 1981 to 2010 to represent average site conditions. We chose water deficit because water balance metrics are of direct physiological importance to plants (71), and initial analyses showed that water deficit was more strongly related to postfire regeneration across multiple species than were precipitation or temperature. To represent interannual variability in postfire conditions, we calculated z-scores (relative to the 1981 to 2010 mean at each site) for three seasonal metrics: growing season water deficit, June to August vapor pressure deficit, and growing season precipitation. Initial comparisons across a broader suite of climate variables showed that these variables captured the range of conditions while minimizing correlations between variables. To represent the most extreme conditions experienced at each site in the first 5 y following fire, when much of the regeneration occurs (24, 72), we took the maximum and the minimum of these metrics over that time period. Climate extremes are better correlated with regeneration than average conditions between 3 and 5 y postfire (22).

Future climate data (Coupled Model Intercomparison Project Phase 5 model outputs) were downscaled using the Multivariate Adaptive Constructed Analogs method version 2 with the gridMET training dataset (73) for the years 2031 to 2050 for five global climate models (GCMs; *SI Appendix*, Table S2). GCMs were selected based on model performance in the western United States and represent a range of possible future conditions that include a wetter and drier scenario (74, 75). Water deficit, seasonal, and postfire climate metrics were calculated with data from each GCM following the same methods as used for the historic climate data.

To account for effects of known disturbances that occurred within 50 y prior to wildfire, prefire disturbance type (none, wildfire, fuel treatment, beetle outbreak, or blowdown) was included in models as a categorical predictor (*SI Appendix*, *Methods*). Some plots likely experienced disturbances within 50 y prior to fire that were not recorded in our dataset. Thus, prefire disturbance was included in models to account for variability in regeneration due to known disturbances, but not to thoroughly investigate the effect of compound disturbances on regeneration.

### Analyses.

We created models of presence/absence of regeneration (at least one conifer seedling per plot) by species and for all species combined using generalized linear mixed effect models with a binomial distribution and a logit link. We modeled *P. ponderosa* and *P. jeffreyi* together as it is difficult to distinguish between seedlings of the two species. We also modeled *A. grandis* and *A. concolor* together given the widespread hybridization of the two species from central Idaho to south-central Oregon (76). Although *P. contorta* was the only study species that can have serotinous cones, we chose to include it in the model with all species because serotiny is highly variable over space, and because the relationship between distance to seed source and recruitment probability was similar between *P. contorta* and other species. We created models in R version 4.0.4 (77) with the glmmTMB package (78). All models included an offset of log(plot size) to account for variation in sampling effort. Simulation-based residuals from the DHARMa package (79) were used to assess model fit and dispersion.

All models included the following biophysical variables as fixed effects: Time since fire, distance to seed source, fire severity, surrounding tree cover, prefire disturbance type, and CHILI. Models included climate variables as described below. We tested for possible quadratic relationships between the response and each variable. For species that have multiple recognized varieties or subspecies, we also included a variety term in the model (*SI Appendix*, Table S5). Each model included a random intercept that varied by wildfire identity to account for lack of independence between observations within the same wildfire.

We started model selection with a full model that included the above biophysical predictors, postfire climate predictors (*SI Appendix*, Table S1), 30-y mean annual or growing season water deficit (depending on species), and interactions between the 30-y mean climate and the postfire climate metrics to account for differential effects of drought across each species range. All predictors included in a single model had Pearson’s correlations <0.6 and variance inflation factors <5. We then used 10-fold cross validation to iteratively remove interaction terms and climate variables to maximize model skill based on cross-validated area under the receiver operating characteristics curve (AUC) (*SI Appendix*, *Methods*). After model selection for climate variables, we checked for interactions between postfire climate variables and fire severity and seed availability (RBR, distance to seed source, surrounding tree cover).

To assess changes over time in recruitment probability, we used our models to predict the mean recruitment probability throughout the study area in three 20-y time periods: 1981 to 2000, 2001 to 2020, and 2031 to 2050. We made projections for each period under two fire severity scenarios (low severity: 10 m distance to seed source, 30% surrounding tree cover, 100 RBR; high severity: 150 m distance to seed source, 10% surrounding tree cover, 400 RBR; *SI Appendix*, *Methods*). We held time since fire and plot size constant at 10 y and 100 m^2^, respectively, for all projections. Recruitment probability thus represents the probability of at least one seedling regenerating in a 0.01-ha plot, which is equivalent to a density of 100 trees ha^−1^. We summarized results of recruitment probability by region, which were defined by aggregating level 3 Environmental Protection Agency ecoregions that contained field sites (Fig. 1*A*). The threshold probability at which recruitment is likely varies between models and between approaches to choosing a threshold (80, 81). In figures, we present thresholds based on methods that maximize kappa (81) and the sum of specificity and sensitivity (80). For Fig. 2*C*, we show the results based on the threshold that maximizes kappa (0.54).

## Supplementary Material

Appendix 01 (PDF)

Dataset S01 (PDF)

## Data Availability

Data will be published and publicly available in the Dryad data repository (https://doi.org/10.5061/dryad.0rxwdbs47). Projections of recruitment probability are publicly viewable here: https://kimberleytaylor7.users.earthengine.app/view/mapping-postfire-conifer-regeneration-probability. Previously published data were used for this work and all citations and links to the data can be found in Dataset S1.
