# Supplementary material for: Reduced fire severity offers near-term buffer to climate-driven declines in conifer resilience across the western United States
Source: Proc Natl Acad Sci U S A. 2023 Mar 6;120(11):e2208120120. doi: 10.1073/pnas.2208120120 (PMC10089158; doi:10.1073/pnas.2208120120)
Supplement: Supplementary file 1 — Appendix 01 (PDF) [file pnas.2208120120.sapp.pdf]

Supplementary Information for

Reduced fire severity offers near-term buffer to climate-driven declines in conifer resilience across the western United States

Kimberley T. Davis, Marcos D. Robles, Kerry B. Kemp, Philip E. Higuera, Teresa Chapman, Kerry L. Metlen, Jamie L. Peeler, Kyle C Rodman, Travis Woolley, Robert N. Addington, Brian J. Buma, C. Alina Cansler, Michael J. Case, Brandon M. Collins, Jonathan D. Coop, Solomon Z. Dobrowski, Nathan S Gill, Collin Haffey, Lucas B. Harris, Brian J. Harvey, Ryan D. Haugo, Matthew D. Hurteau, Dominik Kulakowski, Caitlin E. Littlefield, Lisa A. McCauley, Nicholas Povak, Kristen L. Shive, Edward Smith, Jens T. Stevens, Camille S. Stevens-Rumann, Alan H. Taylor, Alan J. Tepley, Derek J. N. Young, Robert A. Andrus, Mike A. Battaglia, Julia K. Berkey, Sebastian U. Busby, Amanda R. Carlson, Marin E. Chambers, Kyra D. Clark-Wolf, Erich Kyle Dodson, Daniel C. Donato, William M. Downing, Paula J. Fornwalt, Joshua S. Halofsky, Ashley Hoffman, Andres Holz, Jose M. Iniguez, Meg A. Krawchuk, Mark R. Kreider, Andrew J. Larson, Garrett W. Meigs, John Paul Roccaforte, Monica T. Rother, Hugh Safford, Michael Schaedel, Jason S. Sibold, Megan Singleton, Monica G. Turner, Alexandra K. Urza, Larissa Yocom, Joseph B. Fontaine, John L. Campbell

Corresponding author: Kimberley T. Davis
Email: Kimberley.Davis@usda.gov

This PDF file includes:

Supplementary Methods
Figures S1 to S29
Tables S1 to S18
SI References

Supplemental Methods

Distance to seed source

Data from 78% of plots included field measured distance to seed source, defined as distance to the nearest live reproductive tree/s. Most studies measured to the single closest tree but four studies (7% of plots) measured to the nearest 3-10 trees and averaged the distance. For plots without field measured distance to seed source, we manually measured distance to the nearest live tree using 1-m aerial post-fire imagery (National Agriculture Imagery Program) in Google Earth Engine. We selected post-fire imagery from the year that most closely matched the field sampling date. To assess how this method compared to field measured distances we selected 466 random validation plots with field measured distance to seed source to obtain distances with the methods described above. The Pearson correlation between field and aerial-imagery distances was 0.77. Uncertainty may be introduced into our dataset because the majority of studies (61% of plots) measured distance to nearest live tree of any species, whereas the remaining studies measured distance to seed source by species. Sampling locations were generally constrained to forests where the focal species were present, so distance to the nearest live tree of any species is likely similar to distance to conspecifics in most cases. We retained the species-specific data when available in the species-specific models. In the all-species model, we used the shortest distance to seed source of any of the study species as the predictor in the model.

Surrounding tree cover

To represent the overall abundance of live trees (surrounding tree cover), we used the percent tree cover in a 300-m radius around each plot derived from post-fire imagery from the Rangelands Analysis Platform (30-m resolution; 1). The root mean square error for tree cover for this 30-m resolution dataset is 7-10% depending on validation dataset. We chose a 300-m radius because a recent study in *Pinus ponderosa* and dry mixed conifer forests at a subset of our sites (2) conducted a sensitivity analysis and found that when considering surrounding tree cover as a model predictor, a radius of 300 m was more related to post-fire tree regeneration than smaller radii. There has also been evidence in moister mixed conifer forests that post-fire regeneration does not decline until after 200-400 m from live trees (3-5). Additionally, a recent review (6) underscored that dispersal by animals is often neglected in forest disturbance research and that seed dispersal into burned patches may reach farther than expected based on wind-mediated dispersal kernels alone (which often suggest distances closer to 100 m). To understand the implications of using 300 m rather than a smaller radius of 100 m for our surrounding tree cover predictor, we also extracted surrounding tree cover with a radius of 100 m for all study plots. We found that the correlation between the 100-m radius and 300-m radius surrounding tree cover for all plots was 0.90. When we replaced the 300-m radius surrounding tree cover with the 100-m surrounding tree cover in the final all-species model the AUC declined marginally (0.001) and the relationship between recruitment probability and surrounding tree cover was similar.

Pre-fire disturbance

To represent the effect of known disturbances that occurred within 50 years prior to each wildfire, dataset authors included information on recent pre-fire disturbances at their study sites, which included wildfire, prescribed fire, fuel treatments, beetle outbreak or blowdown. We did not include past timber harvest as a disturbance, which likely impacted many of the study sites at some point in the fifty years prior to field sampling. It is likely that some plots experienced other disturbances within the half century prior to the wildfire that were neither recorded nor known

(nor knowable) by dataset authors, and thus pre-fire disturbance was included in models to account for variability in regeneration due to known disturbances, but not necessarily to thoroughly investigate the effect of compound disturbances on regeneration. Sample size of plots with known pre-fire disturbances were small relative to the sample size of plots without a recorded disturbance (Table S6).

Individual species models

To determine if a plot belonged in a model for a given species, we used a combination of field data and raster layers of species basal area (7; Table S7). The raster layers of species basal area were developed by the US Forest Service (USFS) and are based on 30-meter Landsat data, climate, terrain, and soil predictor layers and field data from the USFS Forest Inventory and Analysis plot data (https://www.fs.usda.gov/foresthealth/applied-sciences/mapping-reporting/indiv-tree-parameter-maps.shtml). If field data recorded a live or dead adult or juvenile of a given species the plot was included in the model for that species. We also used the species basal area maps in addition to field data because not all plots had pre-fire stand data and we assumed that a given species may have been present pre-fire in some of these plots even if there was no regeneration. A total of 1,240 plots had no pre-fire stand composition data, and inclusion of these plots in individual species models was based solely on the raster layers of species basal area. This approach could result in an increase in plots with no regeneration of a given species, where adults of that species were not actually present in the field but were predicted to be present based on the raster layer. Alternatively, there are also likely some plots where a species was present prior to fire but the raster data did not indicate the presence of that species. Raster data was initially at 240 m resolution. Based on a sensitivity analysis and comparison to field data, we scaled this to 480 m resolution and designated a species as “present” if it had basal area > 0 in any of the cells within the new 480 m resolution cell.

White fir (*Abies concolor*) and grand fir (*Abies grandis*) hybridize across parts of their ranges in OR and ID and studies have identified two hybrids. Given this tendency, we modeled the two species together and included variety in the model. Here we define varieties following Ott et al. (8) which includes: pure *A. concolor* (Con), pure *A. grandis* (Gra), a hybrid of the two species (Hyb) found in OR and central ID, and *A. concolor* var. *lowiana* (Low) which occupies CA and has admixed ancestry from the hybrid group of the Siskiyou and southern Cascades and *A. concolor*. We also modeled *Pinus ponderosa* and *P. jeffreyi* together given the difficulty in distinguishing between seedlings of the two species where their ranges overlap, as has been done in other studies of post-fire regeneration (9, 10).

Model selection

We created models of presence/absence of regeneration (defined as at least one conifer seedling per plot) by species and for all species combined using generalized linear mixed effect models with a binomial distribution and a logit link. We initiated each of the six species-specific models, as well as the all-species model, with a full model that included post-fire climate predictors from Table S1 and 30-year average (1981-2010) annual or growing season climatic water deficit (depending on species). Post-fire climate predictors represented the maximum or minimum anomaly (z-scores relative to 1981-2010 means) of each predictor (vapor pressure deficit, climatic water deficit, growing season precipitation) that occurred in the first five years post-fire (Table S1). Full models included an interaction between the 30-year mean conditions and the

post-fire climate metrics to account for differential effects of drought across each species range. All predictors included in a single model had Pearson's correlations less than 0.6 and variance inflation factors (VIF) less than five. Where the correlation was >0.6 or VIF>5 we made two full models, one with each of the correlated variables (e.g. one with maximum growing season deficit and one with minimum growing season precipitation; Table S3), and proceeded with the model that had the highest cross-validated AUC. We then used 10-fold cross validation to iteratively remove interaction terms and climate variables to maximize model skill based on cross-validated AUC. If removing an interaction or variable resulted in higher cross-validated model skill, then we removed the interaction/variable that resulted in the greatest increase and repeated the cross-validation until removing interactions/variables reduced model skill. Where cross-validated AUC values were within 0.005 of each other we chose the model with the lowest BIC. Plots located within the same fire were all included within a single fold and predictions to fires used for validation assumed the fire-level intercept was equal to the global intercept. After model selection for climate variables, we checked for interactions between the final post-fire climate variables and metrics related to fire severity and seed availability (RBR, distance to seed source, surrounding tree cover). When variety/subspecies was included in a model, we also assessed for interactions between variety/subspecies and other predictors.

Spatial projections

To create climate inputs for the future projections we calculated 20-year mean annual or growing season climatic water deficit for each period (1981-2000, 2001-2020, 2031-2050). For post-fire climate we calculated z-scores relative to 1981-2010 for each year across the study regions. We then took the rolling 5-year minimum/maximum and averaged that by time period to create predictors that represent the average minimum/maximum climate conditions experienced in the first five post-fire years if a fire were to happen during those time periods. We made future projections using climate outputs from each GCM to allow us to assess variability between GCMs (Fig. S12-S14). Results in the main text show the average recruitment probability across the five GCMs under the RCP 4.5 scenario. Results were similar for RCP 8.5 which are included in the Supplemental Results. The two fire severity scenarios (low severity: 10 m distance to seed source, 30% surrounding tree cover, 100 RBR; high severity: 150 m distance to seed source, 10% surrounding tree cover, 400 RBR; Table S4) were developed based on published values and expert opinion. The RBR values are derived from tables in Parks et al. (11). Distance to seed source and surrounding tree cover were developed based on examining our dataset and discussions among co-authors, who combined have spent thousands of hours in the field in recently burned forests. It is possible for areas to burn at high severity but have a low distance to seed source and high surrounding tree cover if they are in small high severity patches surrounded by areas burned at lower severity. Our scenario instead represents a high severity patch with a distance to seed source of 150 m and 10% surrounding tree cover to better represent a larger high severity patch size, which is becoming increasingly common due to changes in fire regimes and climate (12-14). However, our high severity scenario is still not an extreme situation: the distance to seed source value represents the 82nd highest percentile and the surrounding tree cover the 27th highest percentile measured in plots in our dataset that burned at high severity (RBR>283; (11)). Furthermore, many of the studies in our dataset used a sampling design that involved stratifying plots by distance to seed source which may result in a lower median distance to seed source for high severity plots than would be expected if plots were placed randomly. Past studies have found that post-fire conifer regeneration declines significantly between 40 to 400 m

from a live seed source (2-4, 15), thus 150 m represents a distance that may be limiting for some conifer species but which does not completely preclude seed dispersal. Our high severity scenario does not represent the interior of extremely large high severity patches, where exceptionally low seed availability would severely limit conifer regeneration. Such extremely large patches are increasing, especially under dry conditions (12, 16, 17). We also made initial projections with a with a high severity scenario with distance to seed source set at 75 m and obtained results consistent with the results for the final high severity scenario used here. Our low severity scenario had slightly higher distance to seed source (10 m) and surrounding tree cover (30%) than the median values of the low severity plots in our dataset (5 m and 23%, respectively).

Supplemental figures

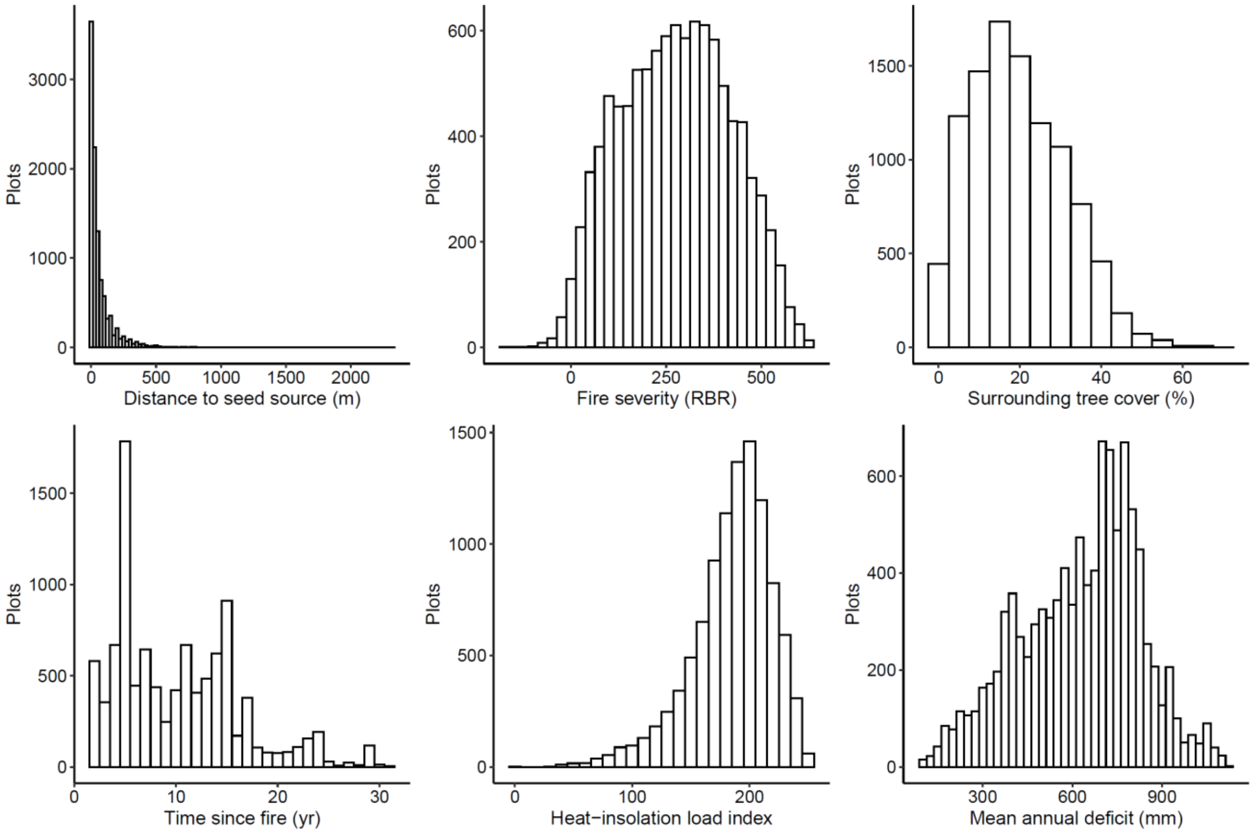

Figure S1. Distribution of sample plots across biophysical predictors. "Deficit" is climatic water deficit and the 30-year mean from 1981-2010 is shown. "RBR" is the relativized burn ratio.

Model output

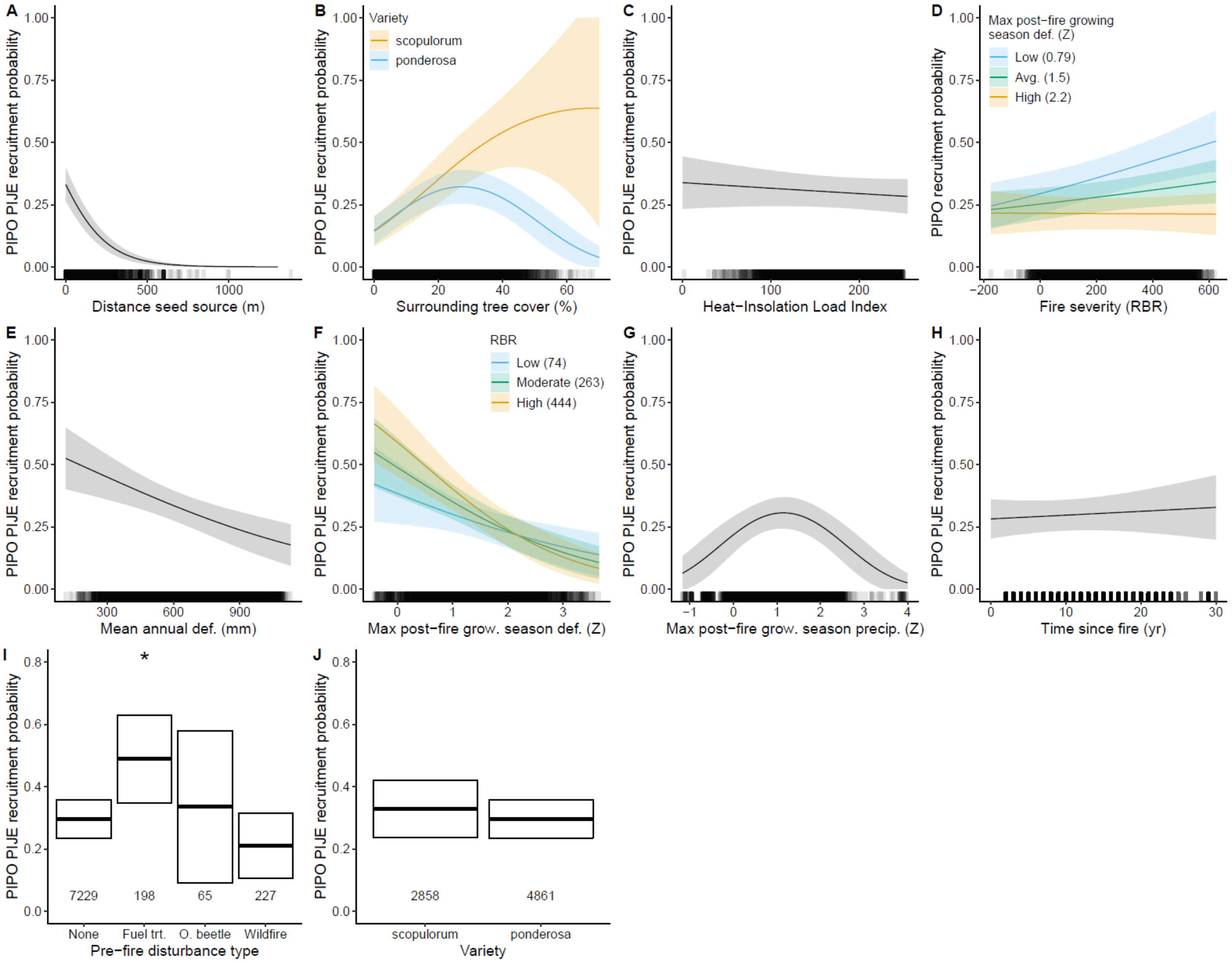

Figure S2. Partial dependence plots for the *Pinus ponderosa/P. jeffreyi* model showing relationship between model predictors and post-fire regeneration while holding other variables constant at their medians. The interaction between RBR and maximum post-fire growing season water deficit ("def."; max within the first 5 post-fire years) is shown by plotting lines for the 10th, 50th, and 90th percentile of RBR from the dataset used to create the model. Significant interactions between the interior (var. *scopulorum*) and coastal (var. *ponderosa*) variety and mean tree cover and RBR are also shown. "O. beetle" is beetles that do not affect ponderosa or Jeffrey pine. "Fuel trt." is fuel reduction treatment. Bands in A-H and boxes in I-J are 95% confidence intervals. "*" indicates significantly different (p<0.05) than no pre-fire disturbance. Rug plot on the x-axis in A-H show the distribution of data. Numbers above x-axis in I-J show sample size for each group.

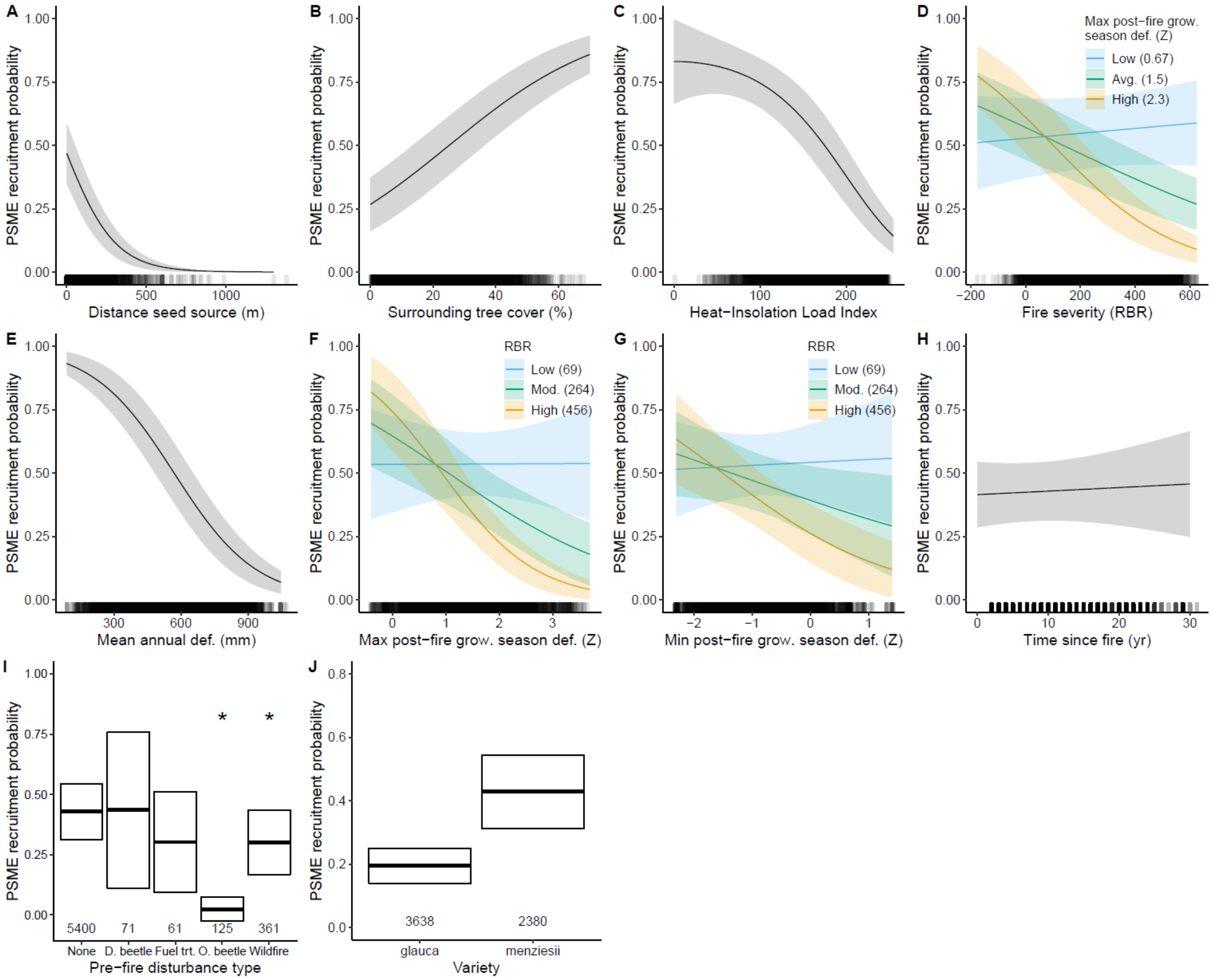

Figure S3. Partial dependence plots for the *Pseudotsuga menziesii* model showing relationship between model predictors and post-fire regeneration while holding other variables constant at their medians. The interaction between fire severity (RBR) and post-fire maximum and minimum growing season water deficit (“def”; max/min within the first five post-fire years) is shown by plotting lines for the 10th, 50th, and 90th percentile of RBR from the dataset used to create the model. Pre-fire disturbance level “D. beetle” is Douglas-fir beetle (*Dendroctonus pseudotsugae*) and “O. beetle” is other types of beetles that do not affect Douglas-fir. “Fuel trt.” is fuel reduction treatment. Bands in A-H and boxes in I-J are 95% confidence intervals. “*” indicates significantly different (p<0.05) than no pre-fire disturbance. Rug plot on the x-axis in A-H show the distribution of data. Numbers above x-axis in I-J show sample size for each group.

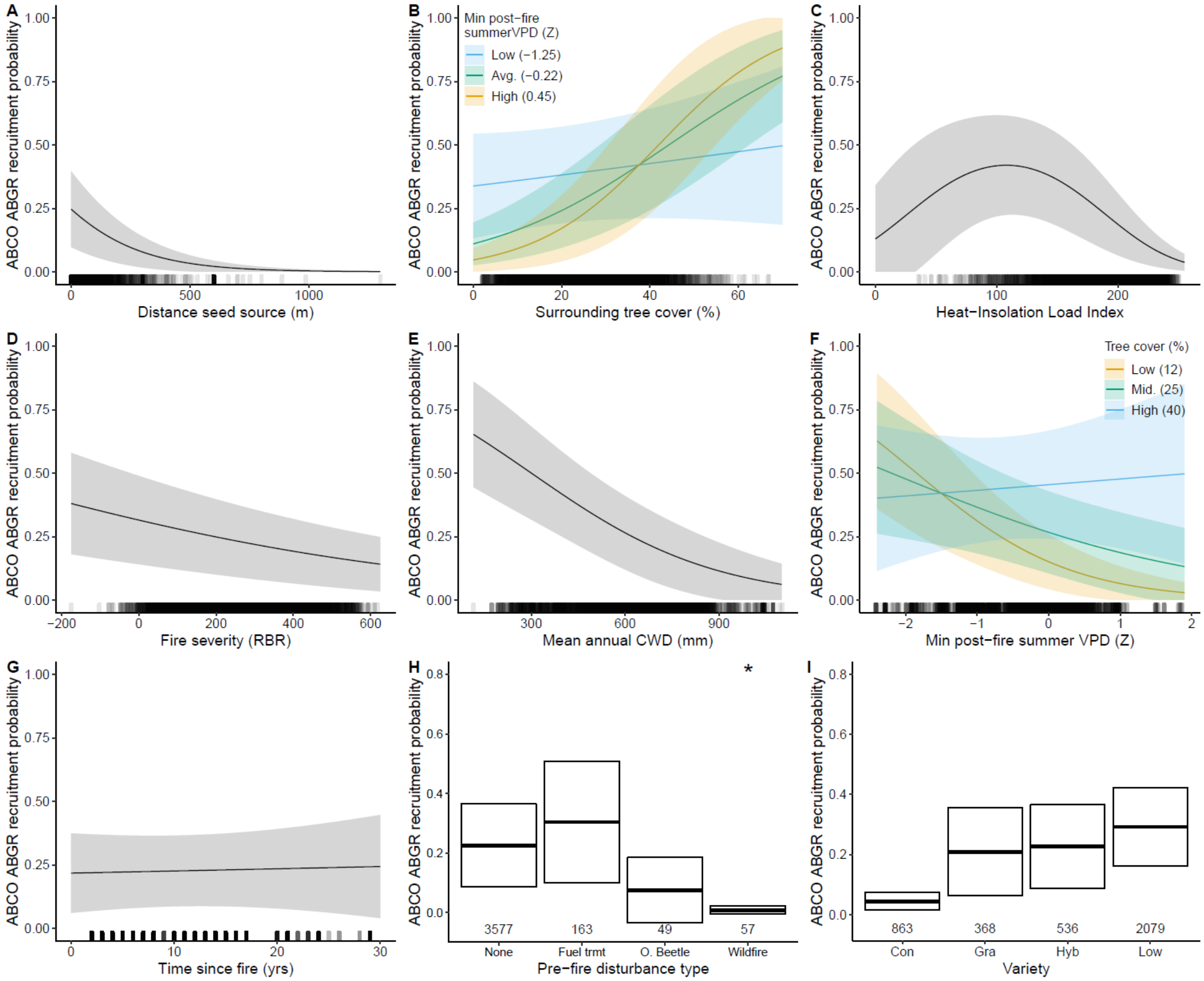

Figure S4. Partial dependence plots for the *Abies concolor/A. grandis* model showing relationship between model predictors and post-fire regeneration while holding other variables constant at their medians and pre-fire disturbance set to "none". The interaction is shown between mean tree cover and the minimum post-fire summer vapor pressure deficit ("VPD"; min within first five post-fire years) by plotting lines for the 10th, 50th, and 90th percentile values of the interacting variable from the dataset used to create the model. "O. beetle" refers to beetles affecting other tree species. Varieties follow Ott et al. (2015) and include: pure *Abies concolor* ("Con"), pure *A. grandis* ("Gra"), a hybrid of the two species ("Hyb"), and *A. concolor* var. *lowiana* ("Low"; see supplemental methods). Bands in A-G and boxes in H-I are 95% confidence intervals. "*" in H indicates significantly different (p<0.05) than no pre-fire disturbance. Rug plot on the x-axis in A-G show the distribution of data. Numbers above x-axis in H-I show sample size for each group.

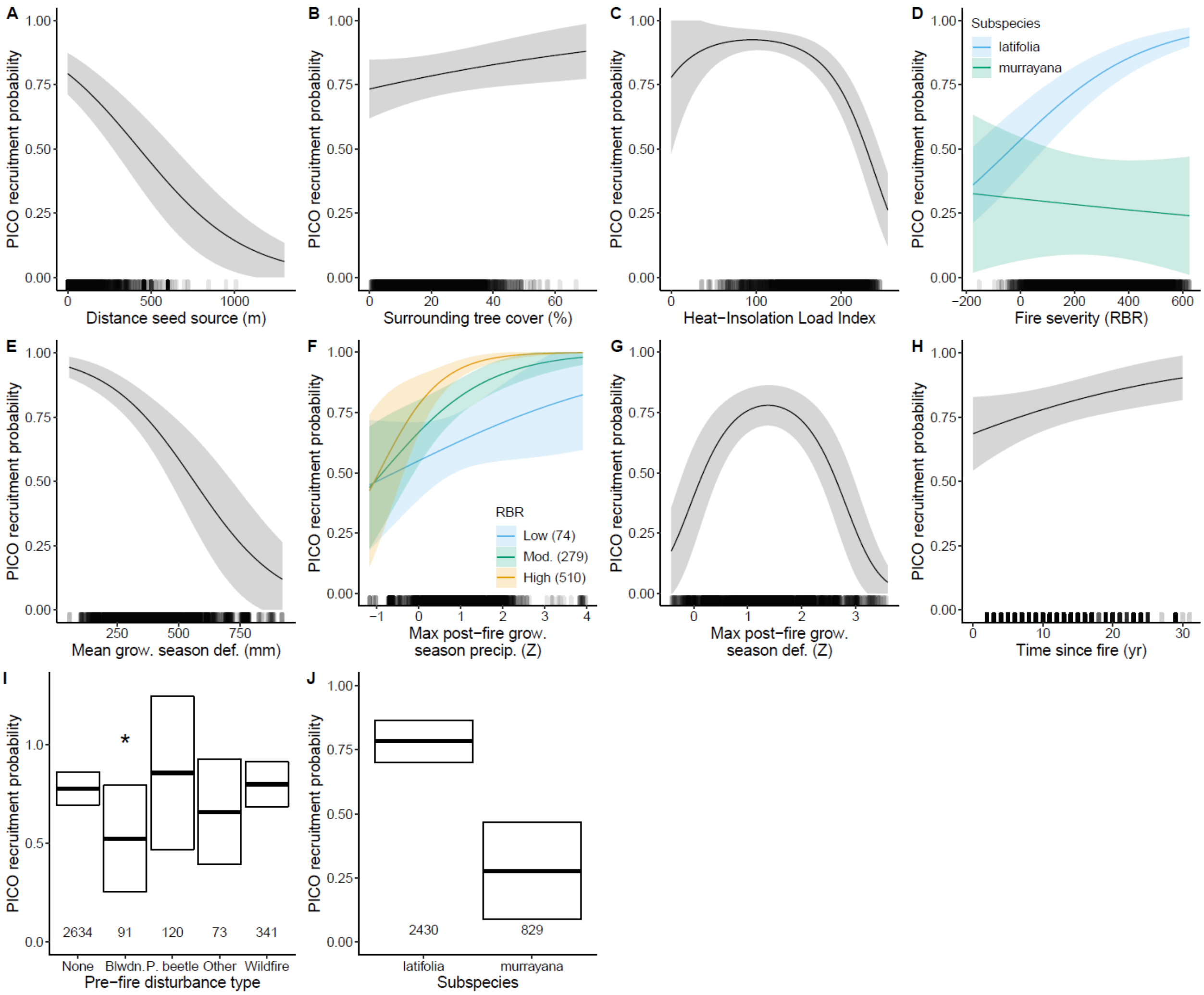

Figure S5. Partial dependence plots for the *Pinus contorta* model showing relationship between model predictors and post-fire regeneration while holding other variables constant at their medians. The interaction between subspecies and RBR is shown. The interaction between RBR and post-fire maximum growing season precipitation (“precip.”; max within the first five post-fire years) is shown by plotting lines for the 10th, 50th, and 90th percentile of RBR from the dataset used to create the model. Pre-fire disturbance level “P. beetle” is mountain pine beetle (*Dendroctonus ponderosae*). The “other” group included other types of beetles that do not affect lodgepole pine (49 plots) and fuel treatments (24 plots) due to small numbers of plots in both categories. “Blwdwn” is blowdown. Bands in A-H and boxes in I-J are 95% confidence intervals. “*” in I indicates significantly different ($p<0.05$) than no pre-fire disturbance. Rug plot on the x-axis in A-H show the distribution of data. Numbers above x-axis in I-J show sample size for each group.

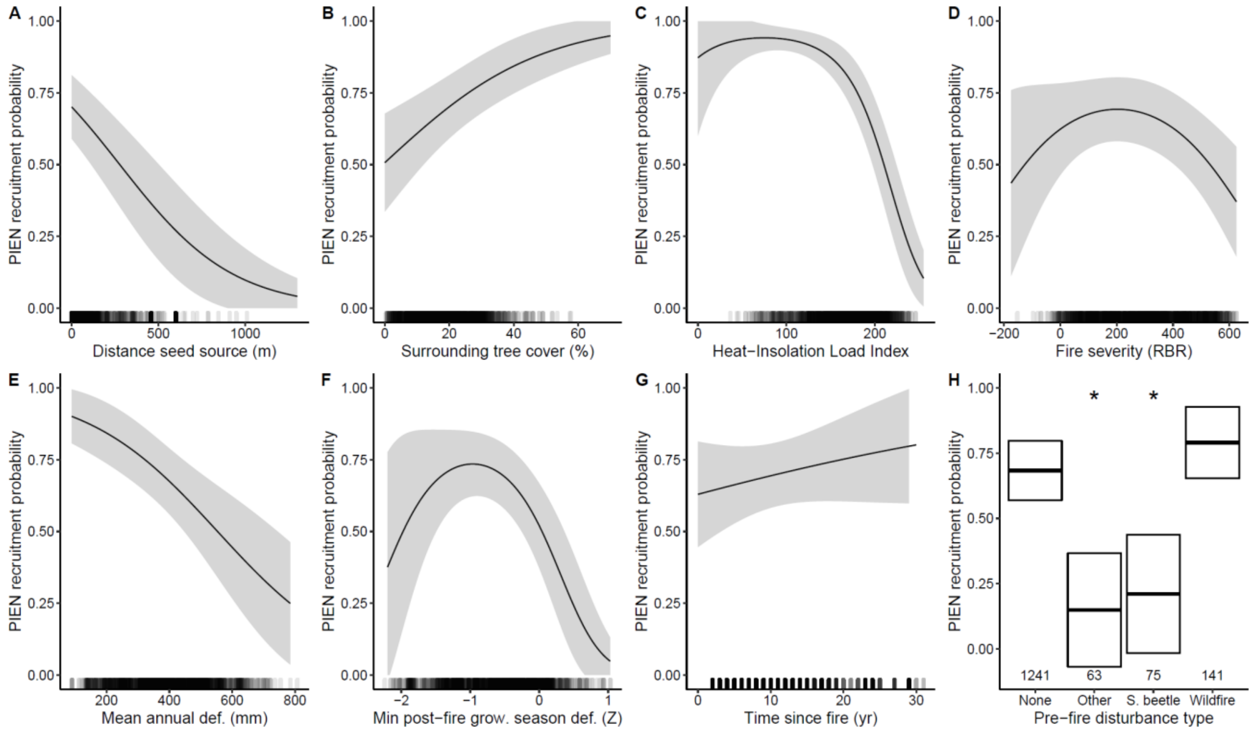

Figure S6. Partial dependence plots for the *Picea engelmannii* model showing relationship between model predictors and post-fire regeneration while holding other variables constant at their medians and pre-fire disturbance set to “none”. The minimum post-fire growing (“grow.”) season water deficit (“def.”) is the minimum of the first five post-fire years. “S. beetle” refers to spruce beetle (*Dendroctonus rufipennis*). “Other” refers to beetles affecting other tree species (10 plots) and blowdown (53 plots). Bands in A-G and boxes in H are 95% confidence intervals. “*” in H indicates significantly different ($p<0.05$) than no pre-fire disturbance. Rug plot on the x-axis in A-G show the distribution of data. Numbers above x-axis in H show sample size for each group.

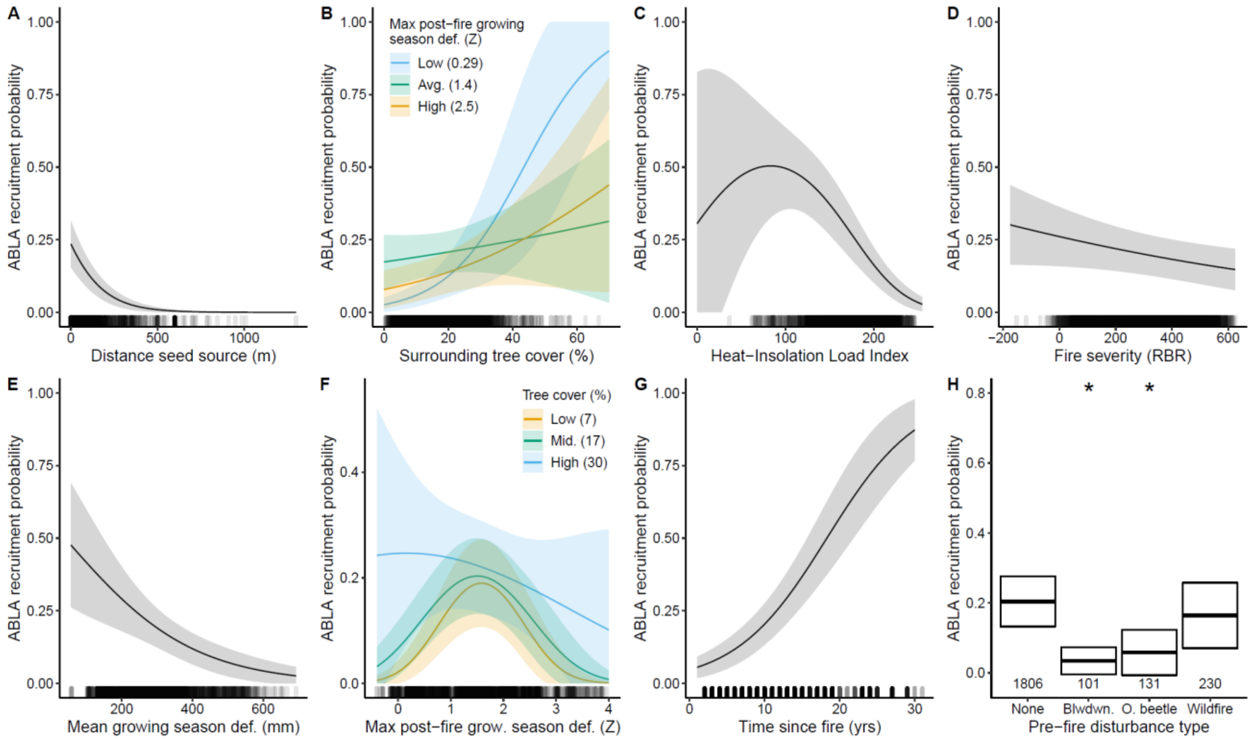

Figure S7. Partial dependence plots for the *Abies lasiocarpa* model showing relationship between model predictors and post-fire recruitment while holding other variables constant at their medians and pre-fire disturbance set to “none”. The interaction is shown between surrounding tree cover and maximum post-fire growing season water deficit (“def.”; max within the first five post-fire years) by plotting lines for the 10th, 50th, and 90th percentile values of the interacting variable from the dataset used to create the model. “Blwdwn” is blowdown. “O. beetle” refers to beetles affecting other tree species. Bands in A-G and boxes in H are 95% confidence intervals. “*” in H indicates significantly different ($p<0.05$) than no pre-fire disturbance. Rug plot on the x-axis in A-G show the distribution of data. Numbers above x-axis in H show sample size for each group.

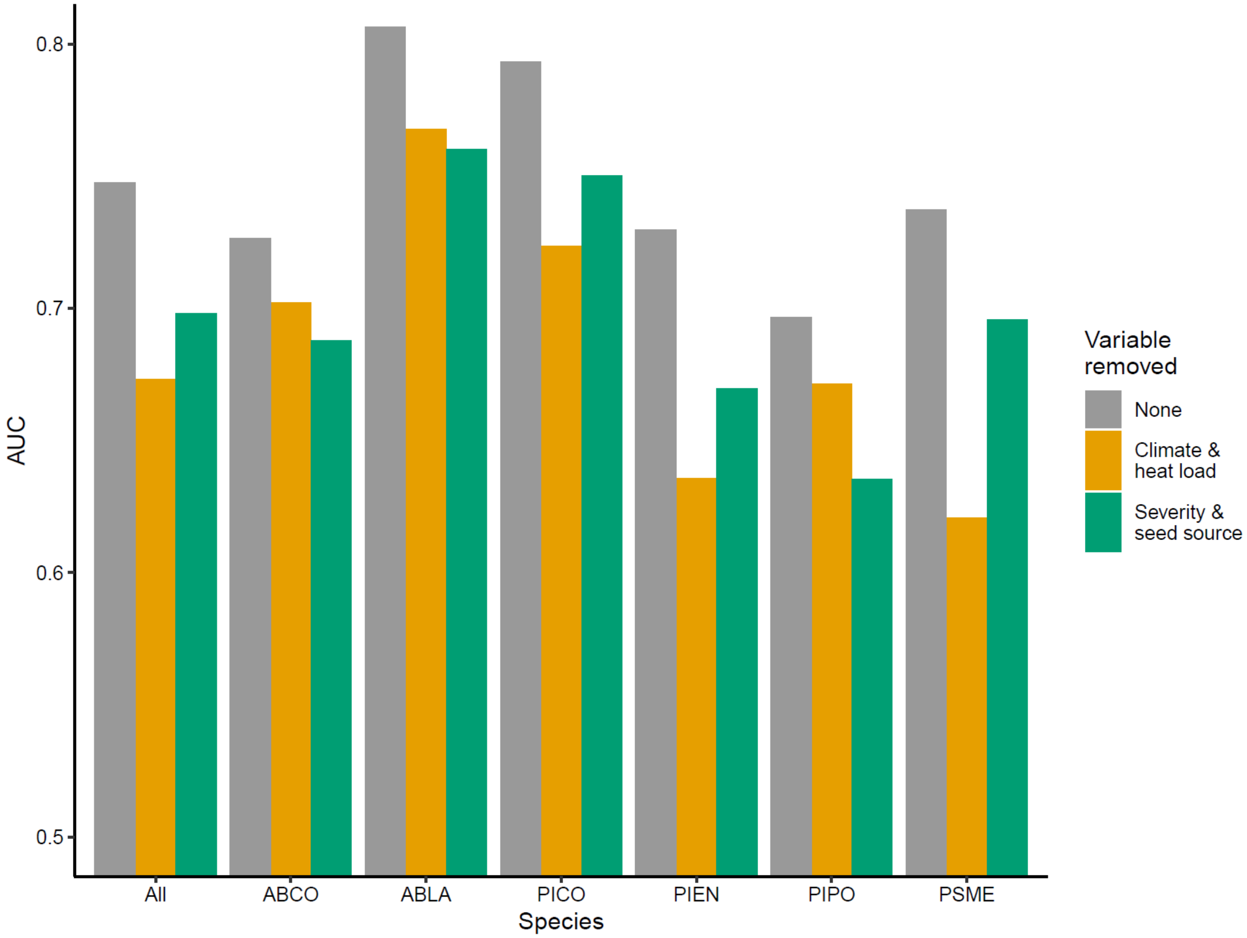

Figure S8. Relative influence of predictor groups on model skill. Bars show the area under the receiver operating characteristic curve (AUC) for the full model (None) and when two groups of variables are removed. “Climate & heat load” includes 30-year mean climatology, post-fire climate and the heat insolation load index (CHILI). “Severity & seed source” includes distance to seed source, satellite-derived fire severity (RBR) and surrounding tree cover. Lower values when a variable group is removed indicates higher relative influence on model skill.

Model projections under different climate and fire severity scenarios

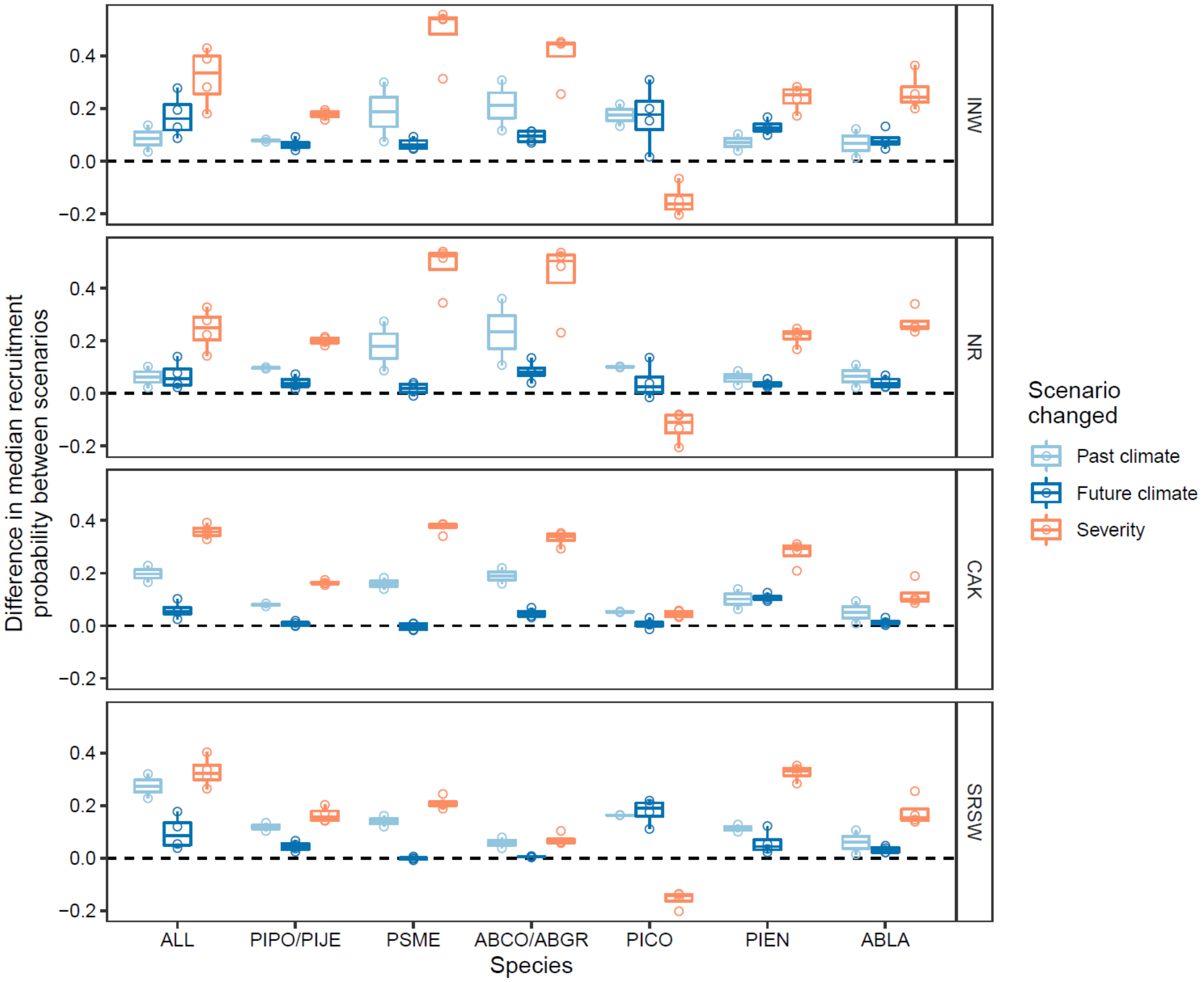

Figure S9. Difference in the median recruitment probability projected for each region and species between successive time periods while holding fire severity scenario constant (Scenario changed: "Past climate" difference between 1981-2000 and 2001-2020, light blue symbols; "Future climate" difference between 2001-2020 and 2031-2050, both RCP scenarios shown, dark blue symbols) or between fire severity scenarios while holding time period constant (orange symbols). Positive values indicate higher median recruitment under earlier time periods (blue climate effect symbols) or under the low severity scenario (orange severity effect symbols).

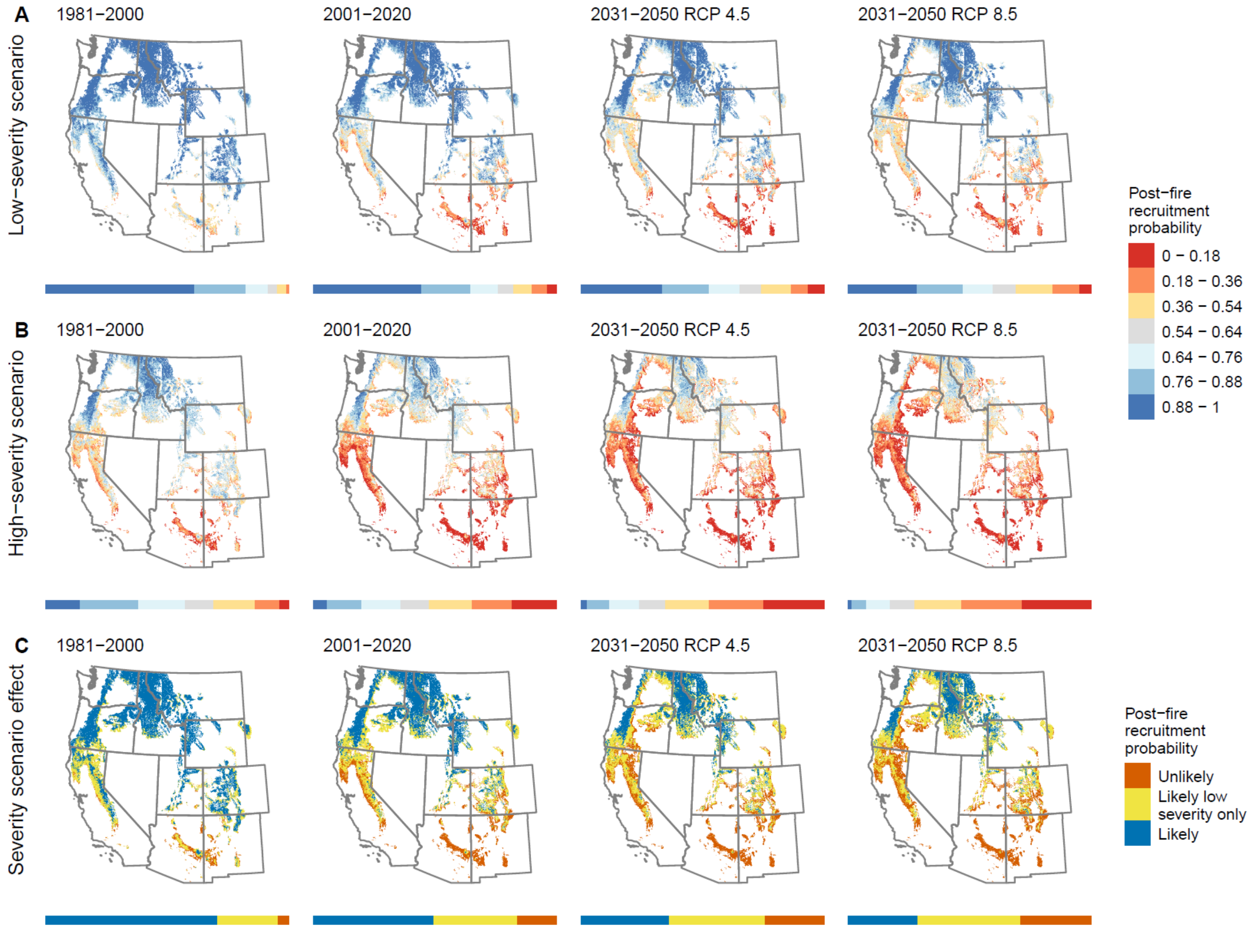

Figure S10. Regional variability in post-fire conifer recruitment under past and future climate and fire-severity scenarios. Post-fire conifer recruitment probability from the all-species model under past and current climate, two future climate scenarios (RCP 4.5 and RCP 8.5), and (A) low- and (B) high-severity scenarios. Bars beneath maps show the proportion of the study area that falls into each category. Shades of blue represent areas where recruitment is likely, whereas warm colors represent areas where recruitment is unlikely. Areas in gray highlight the range of threshold probabilities above which recruitment is likely (see methods; Table S8). (C) Differences in recruitment probability between fire severity scenarios. Map shows where recruitment is unlikely under both fire-severity scenarios (orange), likely under only the low severity scenario (yellow), or likely under both severity scenarios (blue). See Table S18 for proportion of study area that falls in each group.

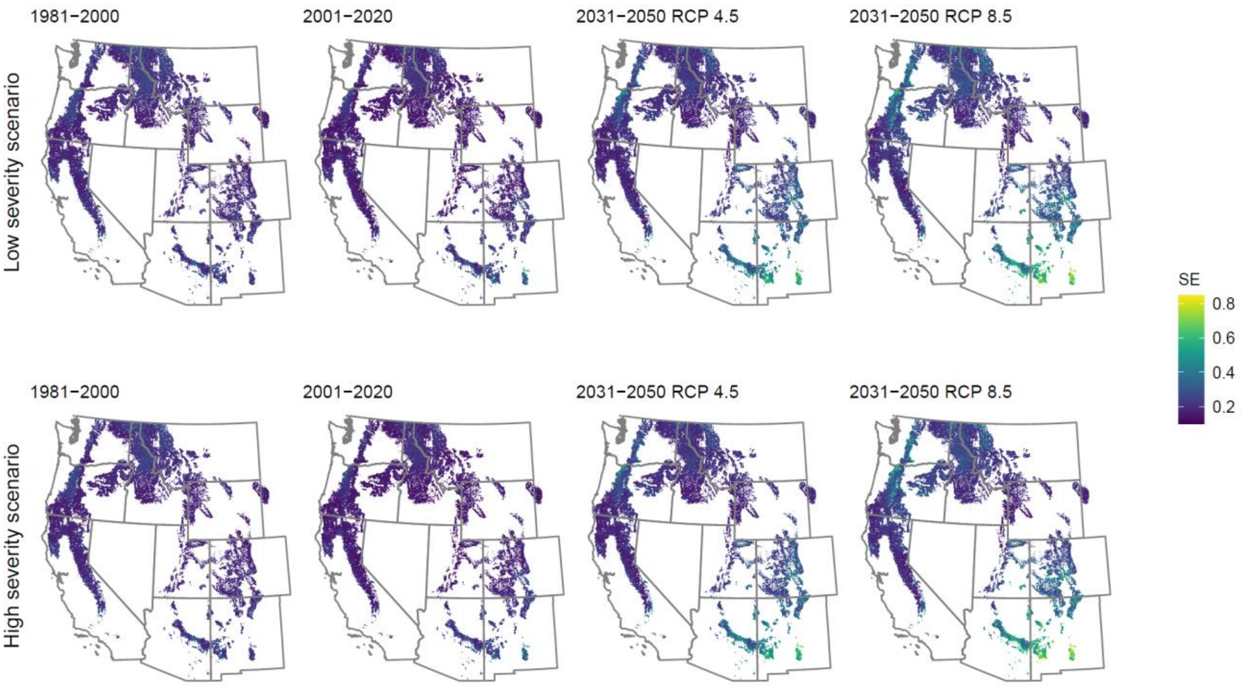

Figure S11. Uncertainty associated with model predictions from the all-species model. Standard error (SE) from the all-species model projected across the study region under past climate, current climate, two future climate scenarios (RCP 4.5 and RCP 8.5), and low- (top row) and high-severity (bottom row) scenarios.

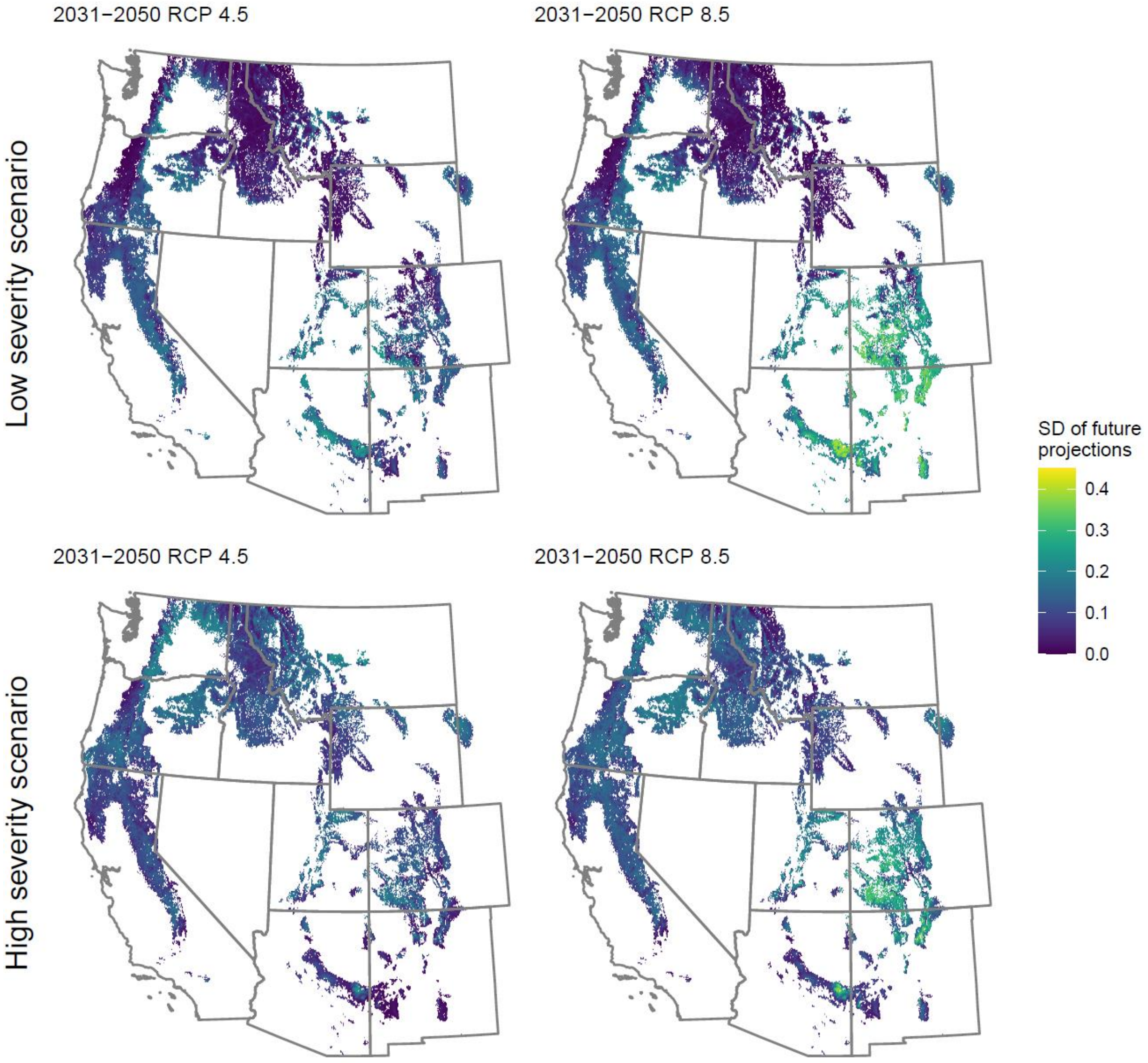

Figure S12. Variability associated with future model projections of recruitment probability from the all-species model. The standard deviation of the five future projections of recruitment probability made with climate data from the five GCMs (Table S2) under each fire severity and RCP scenario.

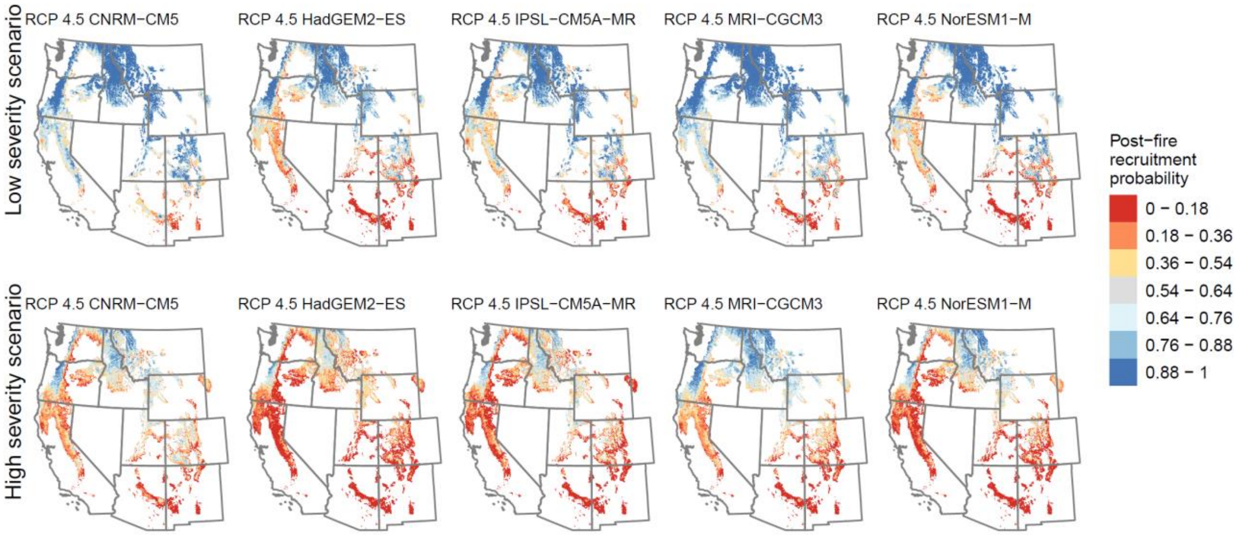

Figure S13. Variability in future recruitment probability. Projections made for 2031-2050 under the RCP 4.5 scenario and either the low fire severity (top row) or high fire severity (bottom row) scenario with climate data from each of five GCMs (Table S2). Shades of blue represent areas where recruitment is likely, whereas warm colors represent areas where recruitment is unlikely. Areas in gray highlight the range of threshold probabilities above which recruitment is likely ($Thresh_{SS}$=0.64, $Thresh_{K}$=0.54; Table S8).

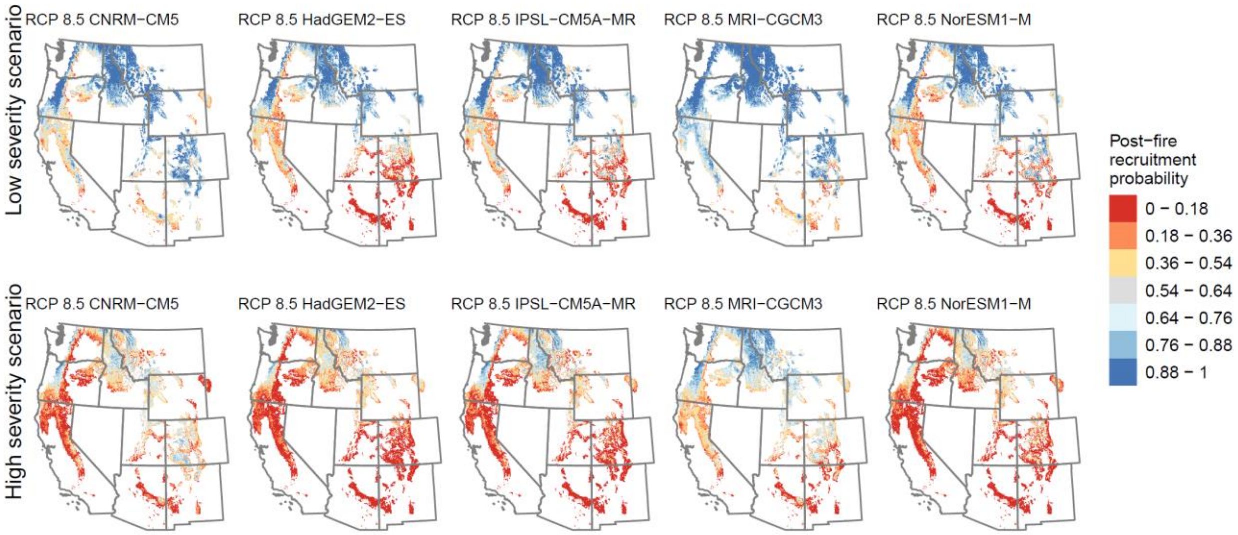

Figure S14. Variability in future recruitment probability. Projections made for 2031-2050 under the RCP 8.5 scenario and either the low fire severity (top row) or high fire severity (bottom row) scenario with climate data from each of five GCMs (Table S2). Shades of blue represent areas where recruitment is likely, whereas warm colors represent areas where recruitment is unlikely. Areas in gray highlight the range of threshold probabilities above which recruitment is likely ($Thresh_{SS}$=0.64, $Thresh_{K}$=0.54; Table S8).

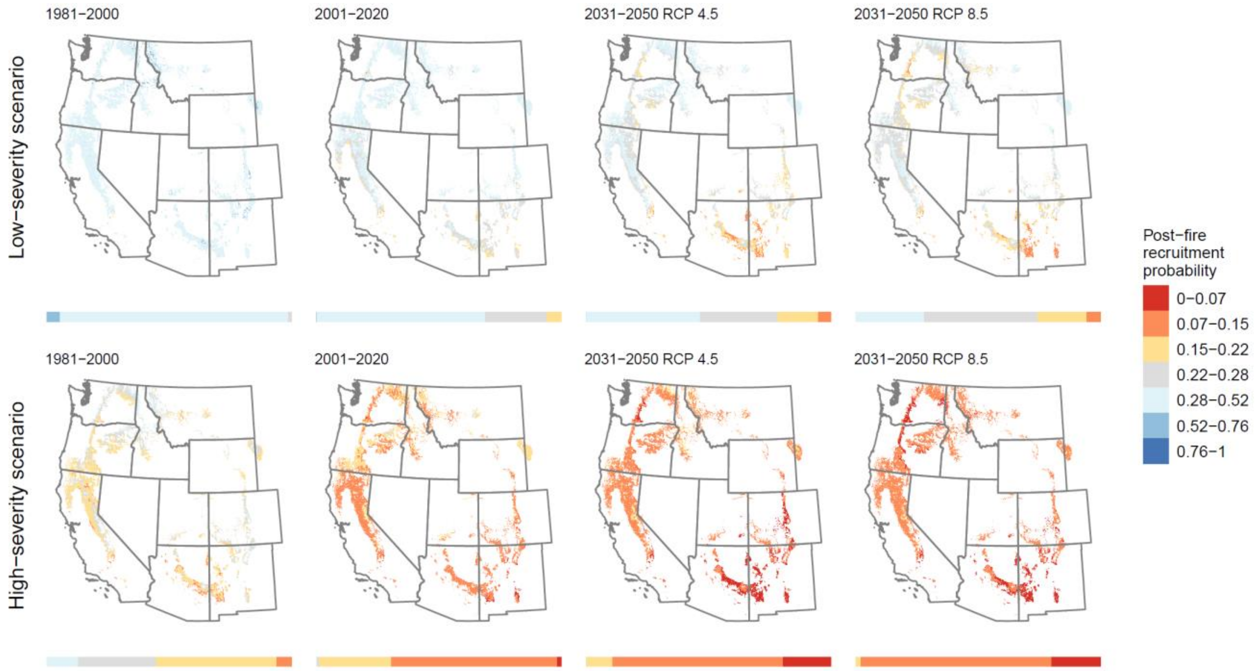

Figure S15. Post-fire recruitment probability from the *Pinus ponderosa/P. jeffreyi* model under past and current climate, future climate scenarios, and low- and high-severity scenarios. Bars beneath maps show the proportion of the study area that falls into each category. Shades of blue represent areas where recruitment is likely, whereas warm colors represent areas where recruitment is unlikely. Areas in gray highlight the range of threshold probabilities above which recruitment is likely ($Thresh_{SS}$=0.22, $Thresh_{K}$=0.28; Table S8).

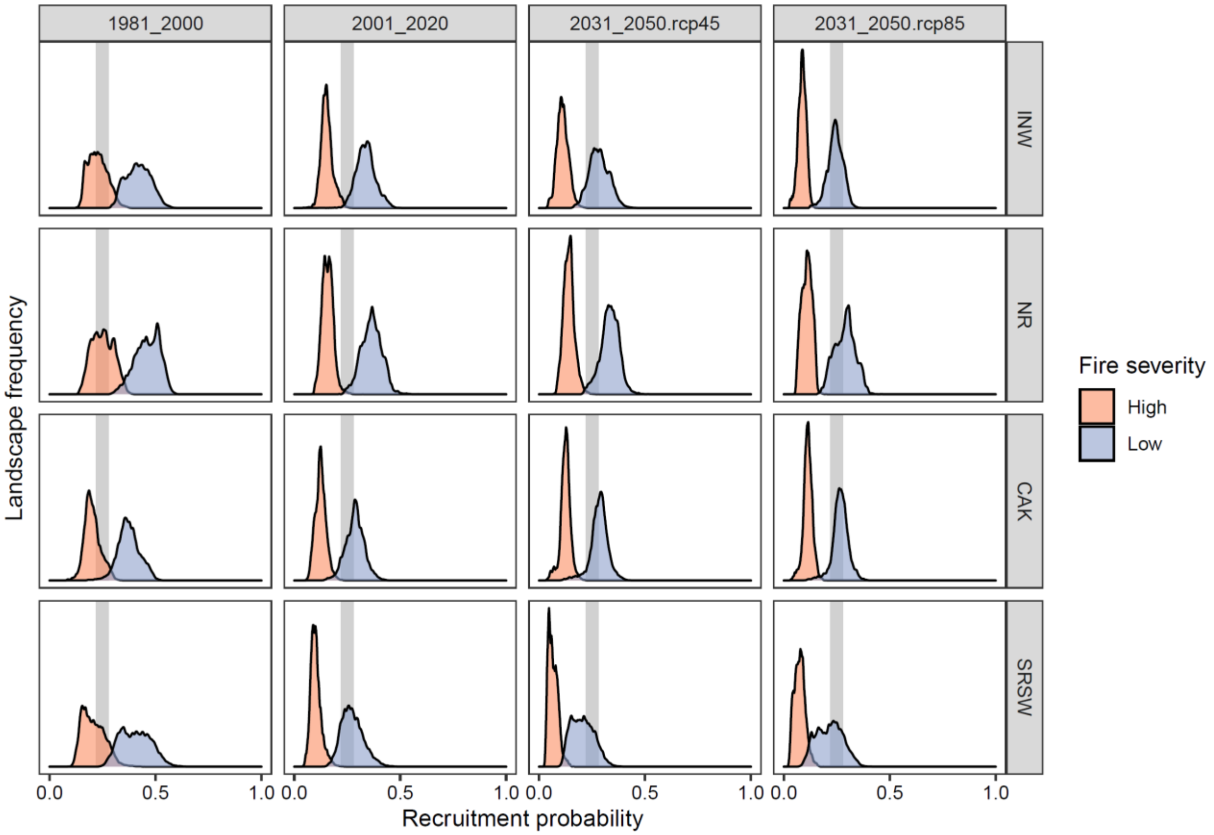

Figure S16. Distribution of *Pinus ponderosa/P. jeffreyi* recruitment probability across their range within each region (rows; INW: interior northwest; NR: northern Rockies; CAK: California and the Klamath; SRSW: southern Rockies and AZ/NM mountains). Different colors represent the fire severity scenarios. Gray vertical shading highlights the range of the probability thresholds above which recruitment is likely ($Thresh_{SS}$=0.22, $Thresh_{K}$=0.28; Table S8).

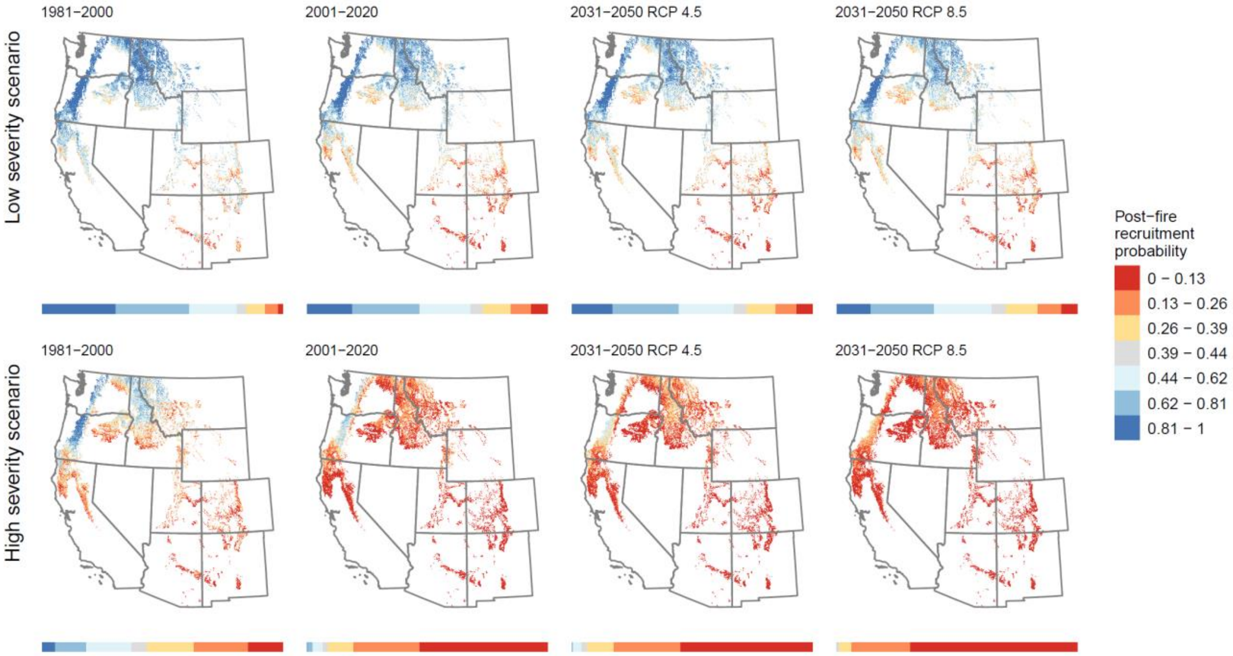

Figure S17. Post-fire recruitment probability from the *Pseudotsuga menziesii* model under past and current climate, future climate scenarios, and low- and high-severity scenarios. Bars beneath maps show the proportion of the study area that falls into each category. Shades of blue represent areas where recruitment is likely, whereas warm colors represent areas where recruitment is unlikely. Areas in gray highlight the range of threshold probabilities above which recruitment is likely ($Thresh_{ss}$=0.39, $Thresh_K$=0.44; Table S8).

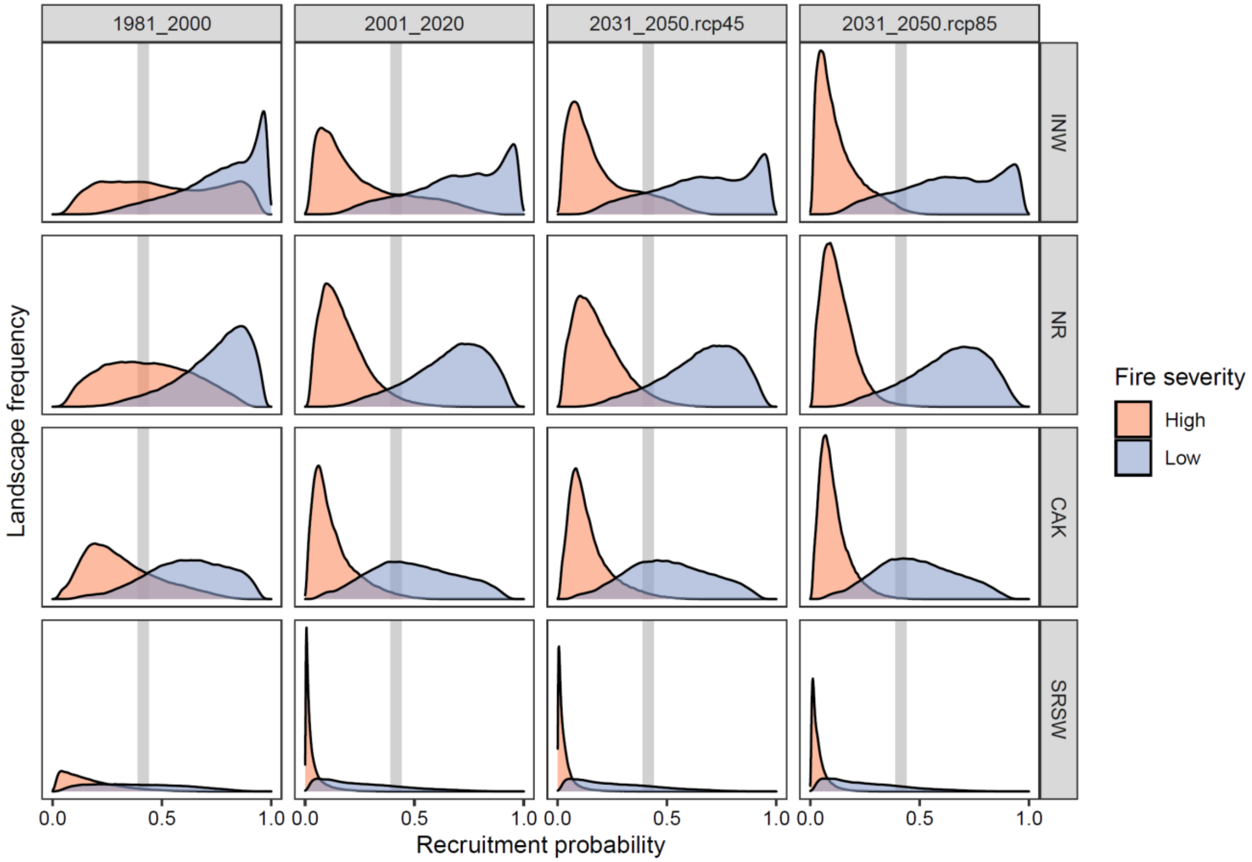

Figure S18. Distribution of *Pseudotsuga menziesii* recruitment probability across its range within each region (rows; INW: interior northwest; NR: northern Rockies; CAK: California and the Klamath; SRSW: southern Rockies and AZ/NM mountains). Different colors represent the fire severity scenarios. Gray vertical shading highlights the range of the probability thresholds above which recruitment is likely ($Thresh_{SS}$=0.39, $Thresh_{K}$=0.44; Table S8).

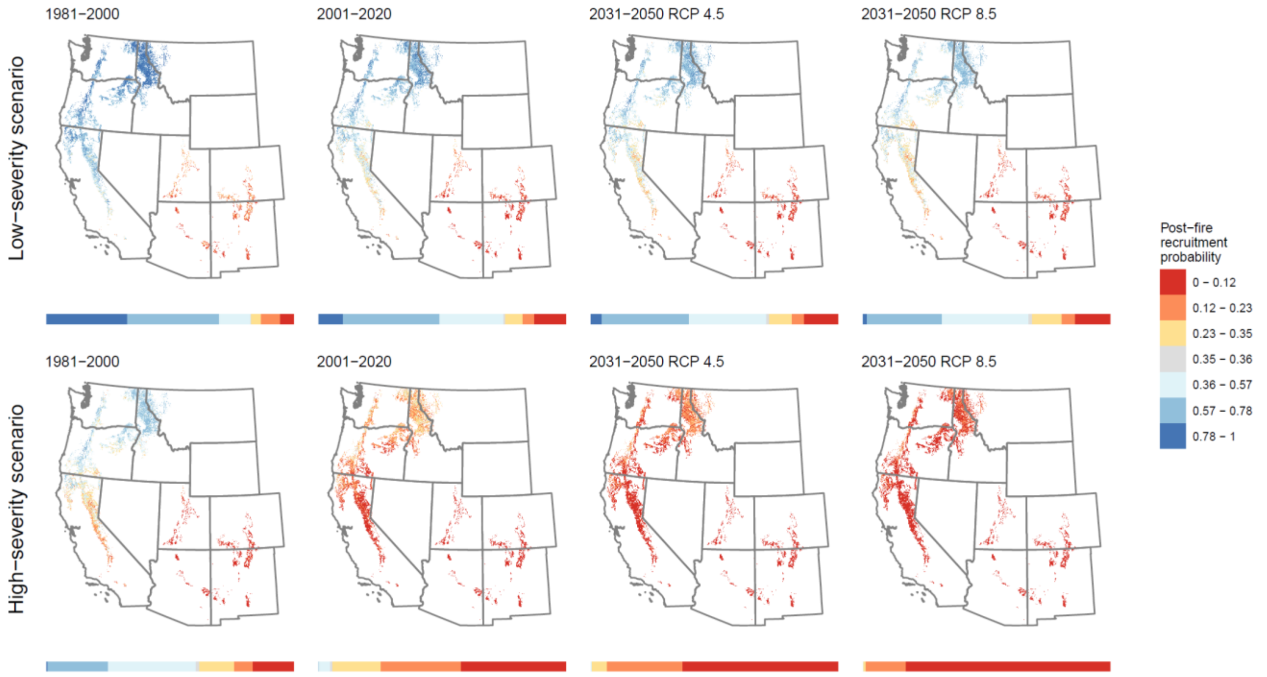

Figure S19. Post-fire recruitment probability from the *Abies concolor/A. grandis* model under past and current climate, future climate scenarios, and low- and high-severity scenarios. Bars beneath maps show the proportion of the study area that falls into each category. Shades of blue represent areas where recruitment is likely, whereas warm colors represent areas where recruitment is unlikely. Areas in gray highlight the range of threshold probabilities above which recruitment is likely ($Thresh_{SS}$=0.35, $Thresh_{K}$=0.36; Table S8).

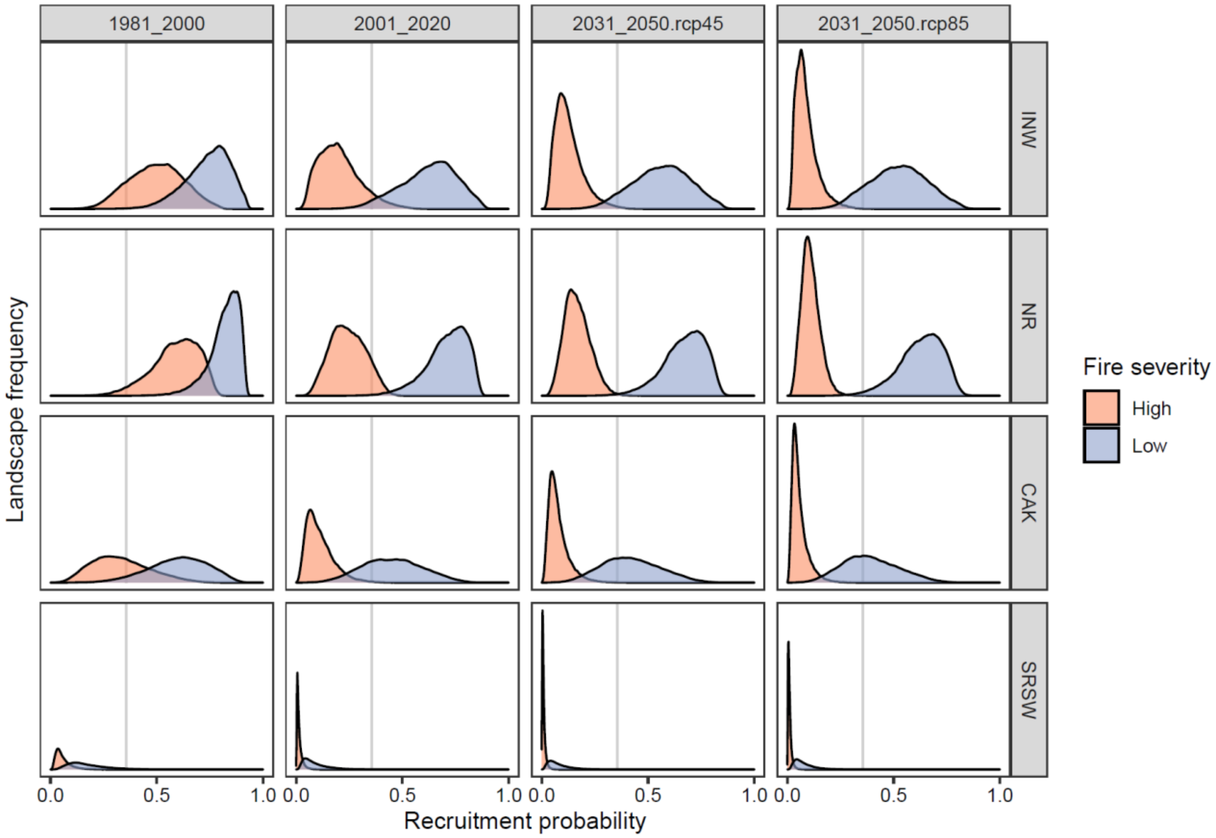

Figure S20. Distribution of *Abies concolor/A. grandis* recruitment probability across their range within each region (rows; INW: interior northwest; NR: northern Rockies; CAK: California and the Klamath; SRSW: southern Rockies and AZ/NM mountains). Different colors represent the fire severity scenarios. Gray vertical shading highlights the range of the probability thresholds above which recruitment is likely ($Thresh_{SS}$=0.35, $Thresh_{K}$=0.36; Table S8).

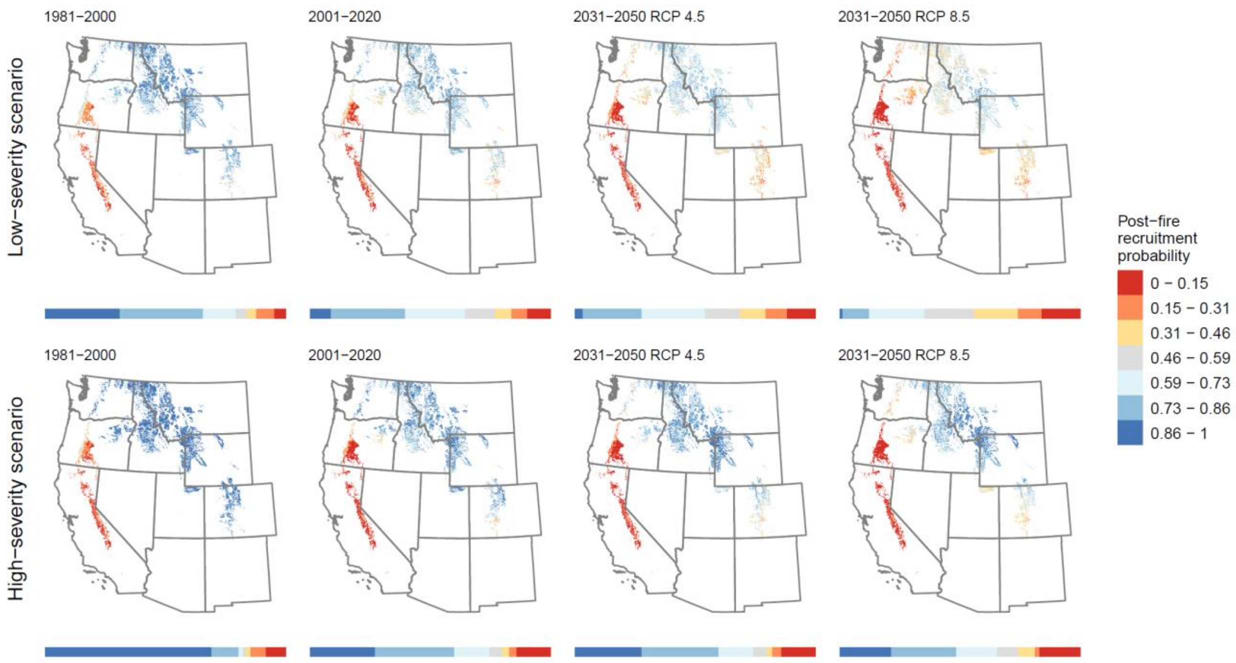

Figure S21. Post-fire recruitment probability from the *Pinus contorta* model under past and current climate, future climate scenarios, and low- and high-severity scenarios. Bars beneath maps show the proportion of the study area that falls into each category. Shades of blue represent areas where recruitment is likely, whereas warm colors represent areas where recruitment is unlikely. Areas in gray highlight the range of threshold probabilities above which recruitment is likely ($Thresh_{ss}$=0.59, $Thresh_{K}$=0.46; Table S8).

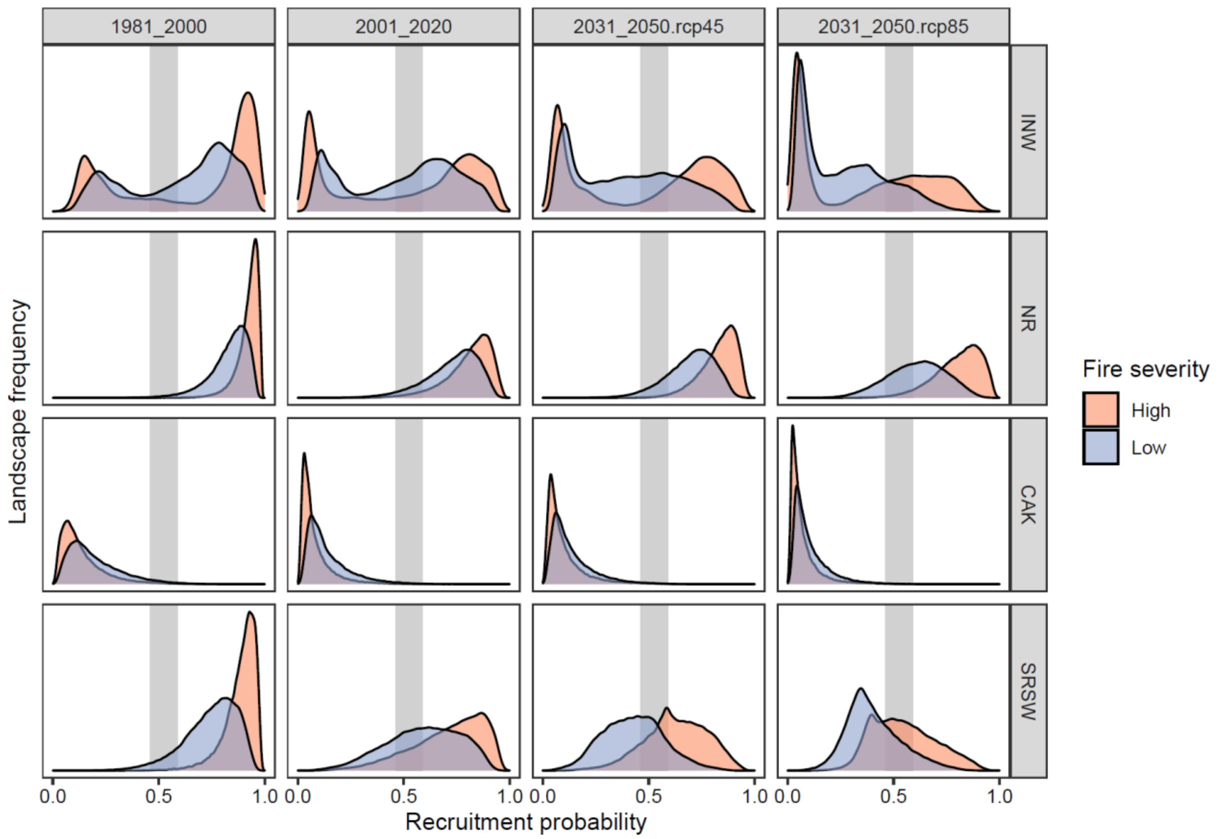

Figure S22. Distribution of *Pinus contorta* recruitment probability across its range within each region (rows; INW: interior northwest; NR: northern Rockies; CAK: California and the Klamath; SRSW: southern Rockies and AZ/NM mountains). Different colors represent the fire severity scenarios. Gray vertical shading highlights the range of the probability thresholds above which recruitment is likely ($Thresh_{SS}$=0.59, $Thresh_K$=0.46; Table S8).

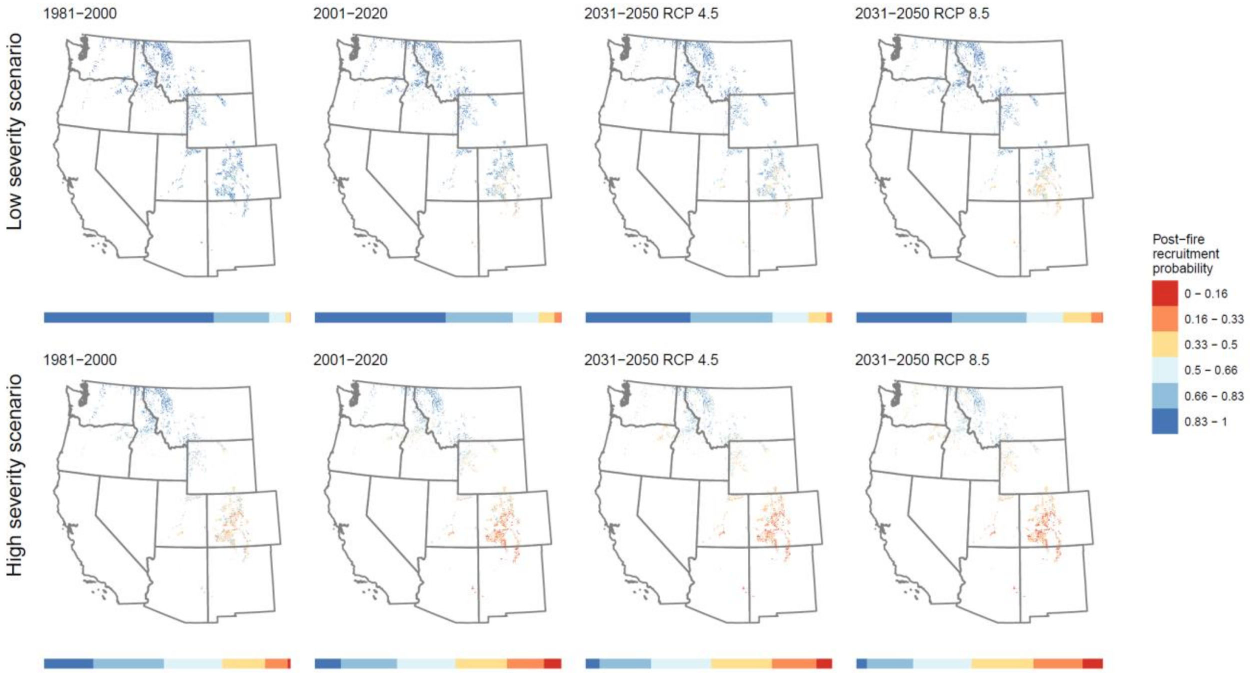

Figure S23. Post-fire recruitment probability from the *Picea engelmannii* model under past and current climate, future climate scenarios, and low- and high-severity scenarios. Bars beneath maps show the proportion of the study area that falls into each category. Shades of blue represent areas where recruitment is likely, whereas warm colors represent areas where recruitment is unlikely. The threshold probability at which recruitment is likely was the same for the approach that maximizes kappa and the approach that maximizes the sum of specificity and sensitivity ($Thresh_{SS}$=0.50, $Thresh_{K}$=0.50; Table S8).

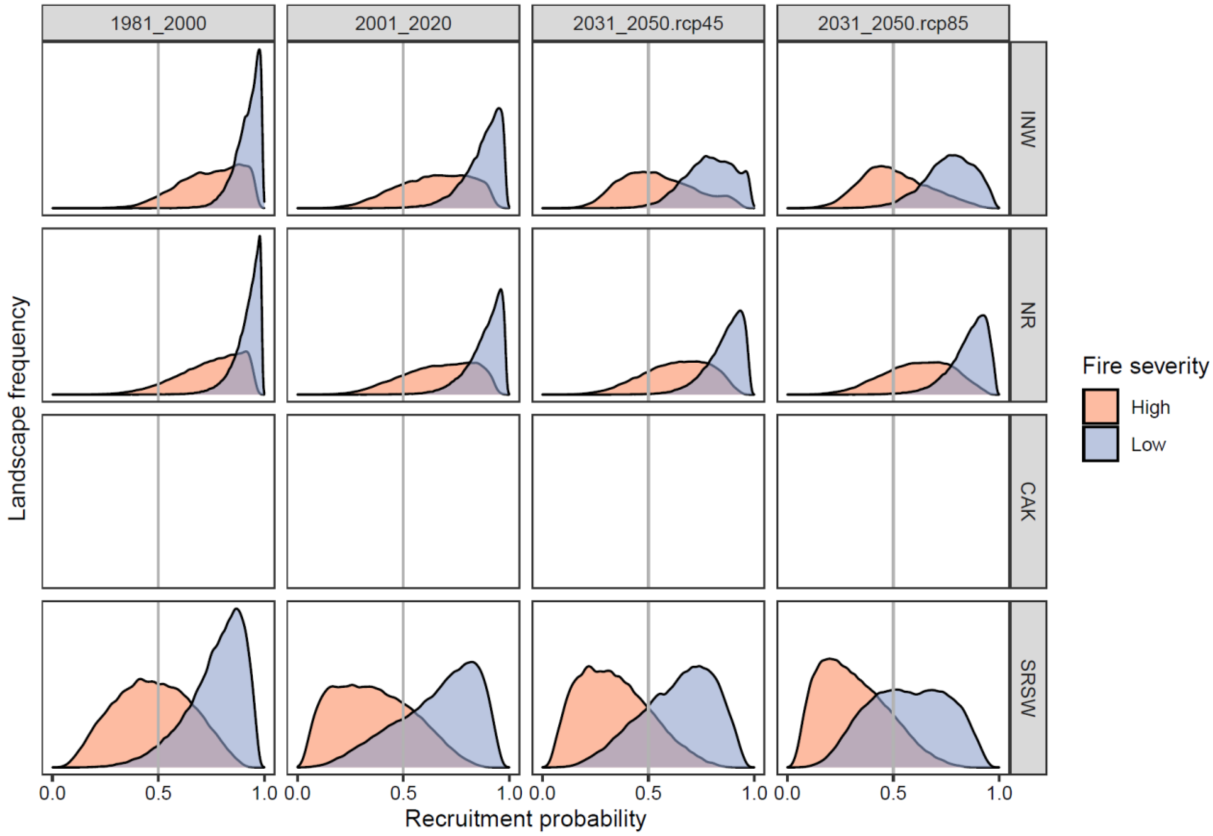

Figure S24. Distribution of *Picea engelmannii* recruitment probability across its range within each region (rows; INW: interior northwest; NR: northern Rockies; CAK: California and the Klamath; SRSW: southern Rockies and AZ/NM mountains). Different colors represent the fire severity scenarios. Gray vertical line shows the probability threshold above which recruitment is likely ($Thresh_{SS}$=0.5, $Thresh_{K}$=0.5; Table S8).

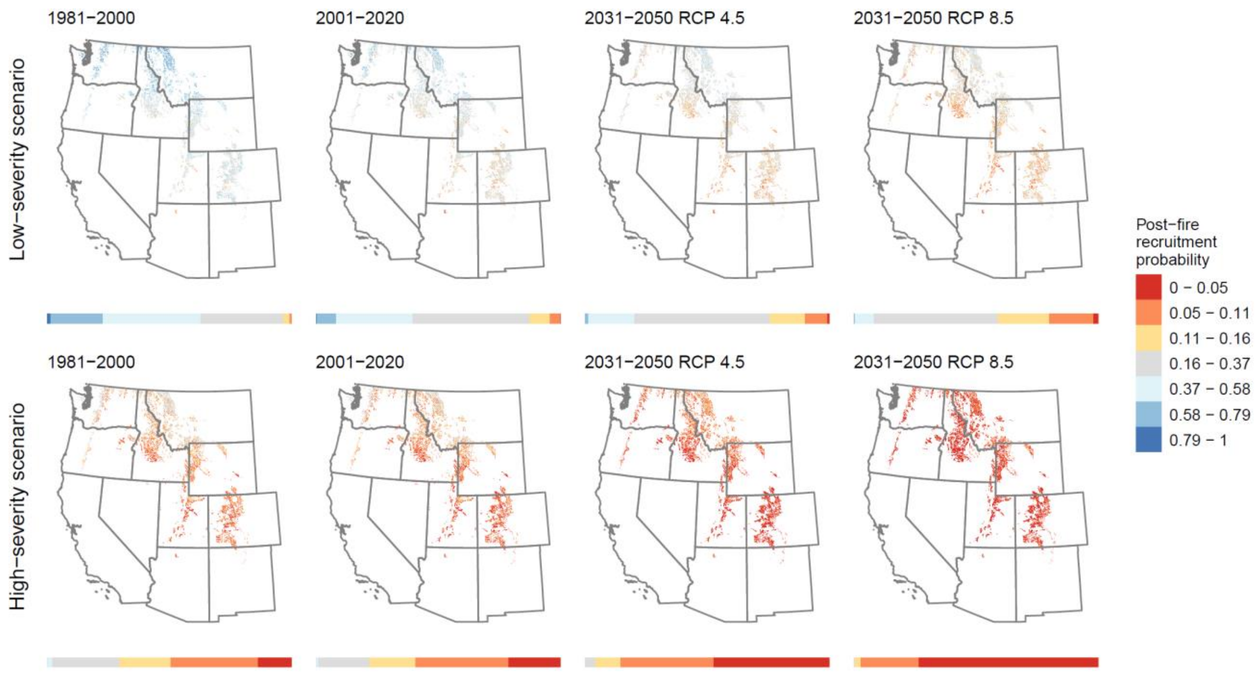

Figure S25. Post-fire recruitment probability from the *Abies lasiocarpa* model under past and current climate, future climate scenarios, and low- and high-severity scenarios. Bars beneath maps show the proportion of the study area that falls into each category. Shades of blue represent areas where recruitment is likely, whereas warm colors represent areas where recruitment is unlikely. Areas in gray highlight the range of threshold probabilities above which recruitment is likely ($Thresh_{SS}$=0.16, $Thresh_{K}$=0.37; Table S8).

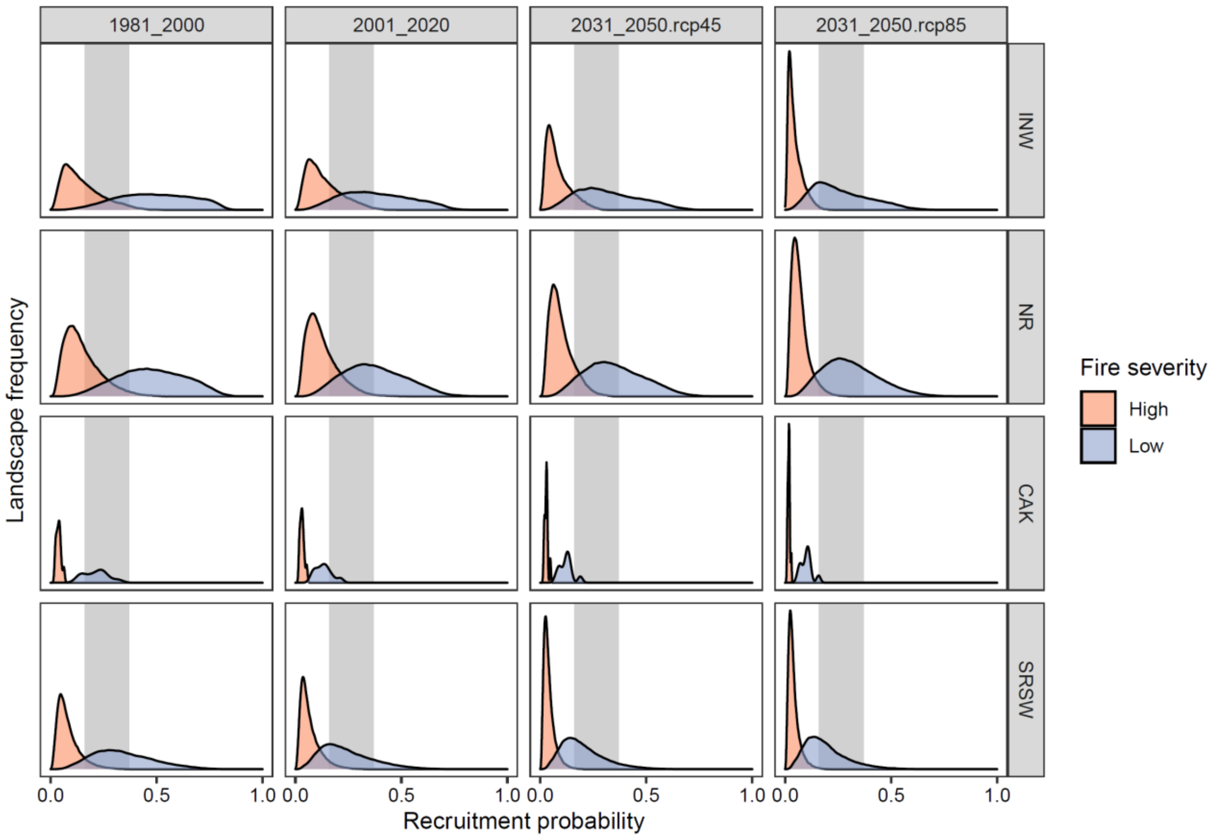

Figure S26. Distribution of *Abies lasiocarpa* recruitment probability across its range within each region (rows; INW: interior northwest; NR: northern Rockies; CAK: California and the Klamath; SRSW: southern Rockies and AZ/NM mountains). Different colors represent the fire severity scenarios. Gray vertical shading highlights the range of the probability thresholds above which recruitment is likely ($Thresh_{SS}$=0.16, $Thresh_{K}$=0.37; Table S8).

Timeseries of post-fire climate data

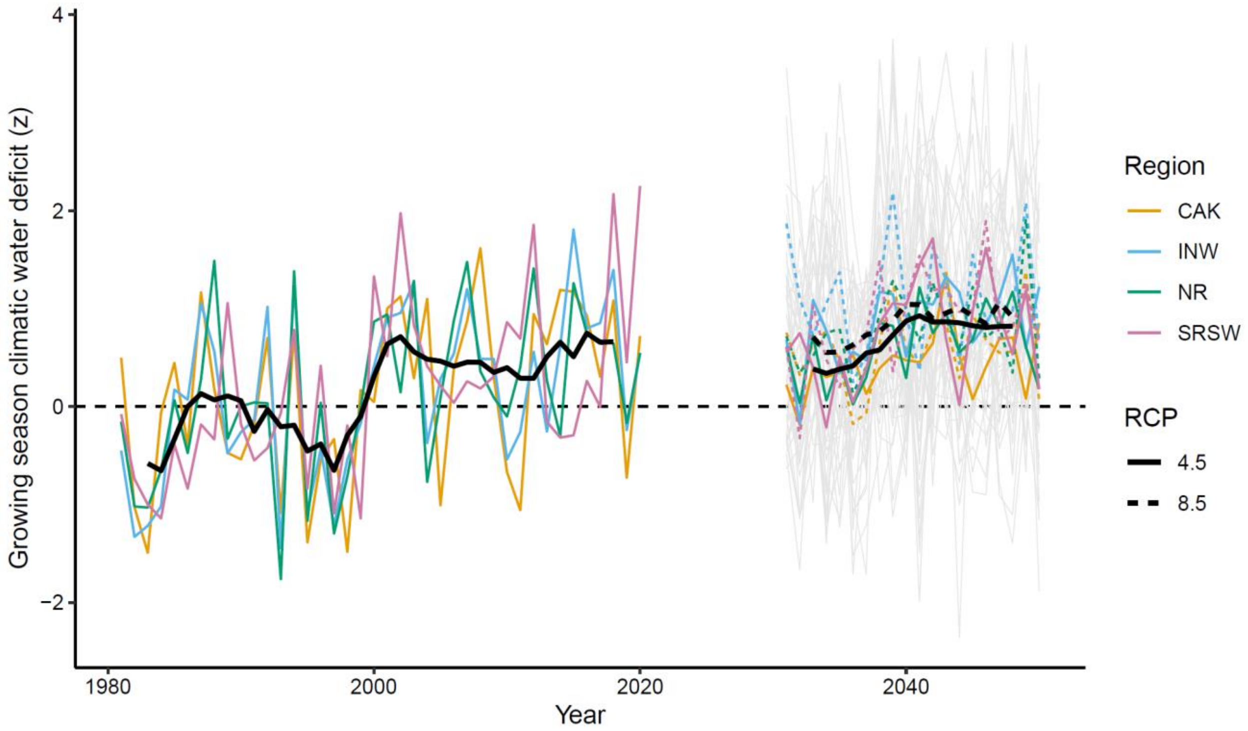

Figure S27. Time series of growing season (April-September) climatic water deficit anomaly (Z, relative to 1981-2010) across the range of the study species in the study region (Fig. 1A) and averaged by region (see Fig. 1A). Gray lines for future data show all individual combinations of region, GCM and RCP. Colored lines for future data are averaged across the five GCMs by region (Table S2). Black lines are the five-year rolling mean across all regions and by RCP scenario for future data. CAK: California and the Klamath; INW: interior northwest; NR: northern Rockies; SRSW: southern Rockies and AZ/NM mountains.

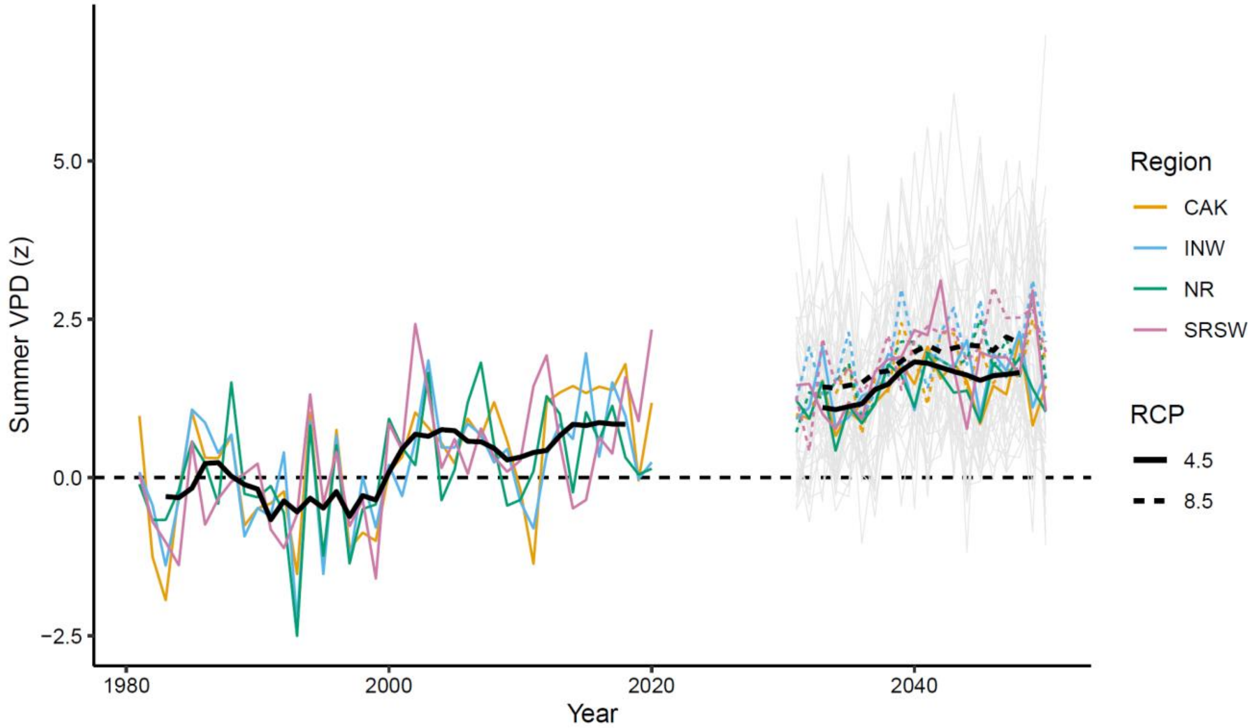

Figure 28. Time series of summer (June-August) vapor pressure deficit (VPD; Z, relative to 1981-2010) across the range of the study species in the study regions and averaged by region (see Fig. 1A). Gray lines for future data show all individual combinations of region, GCM and RCP. Colored lines for future data are averaged across the five GCMs by region (Table S2). Black lines are the five-year rolling mean across all regions and by RCP scenario for future data. CAK: California and the Klamath; INW: interior northwest; NR: northern Rockies; SRSW: southern Rockies and AZ/NM mountains.

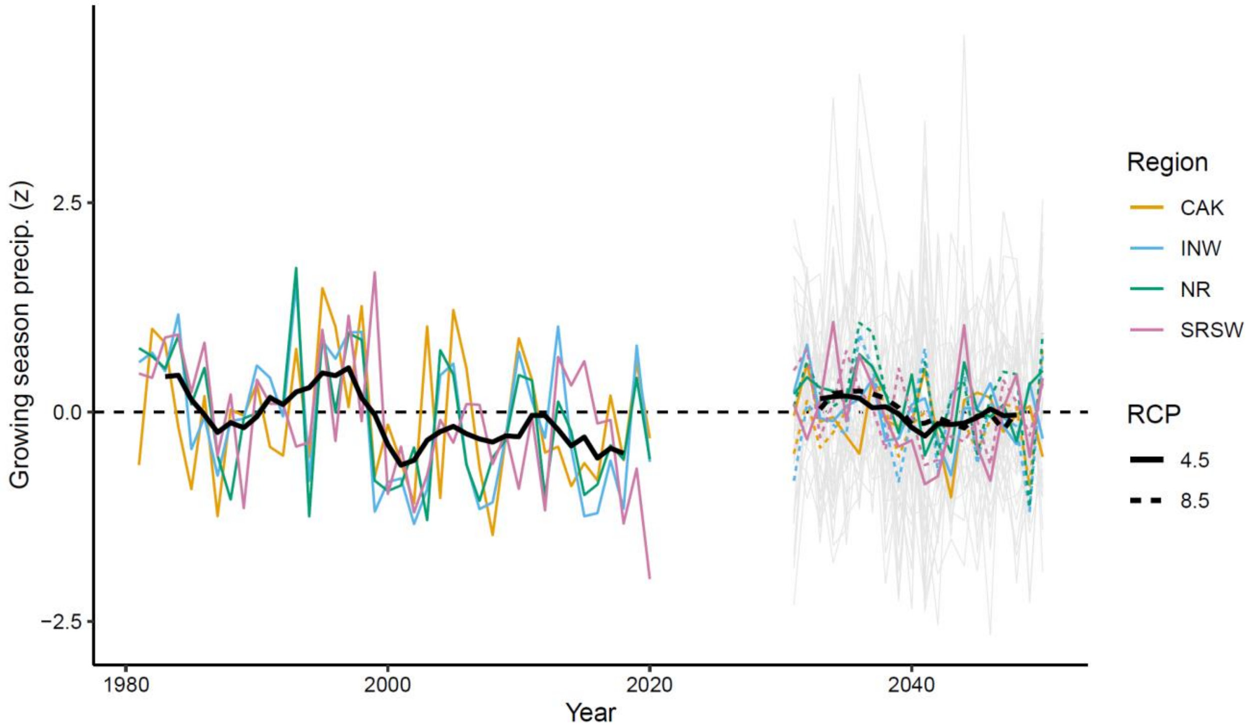

Figure S29. Time series of growing season (April-September) precipitation (precip.; Z, relative to 1981-2010) across the range of the study species in the study regions and averaged by region (see Fig. 1A). Gray lines for future data show all individual combinations of region, GCM and RCP. Colored lines for future data are averaged across the five GCMs by region (Table S2). Black lines are the five-year rolling mean across all regions and by RCP scenario for future data. CAK: California and the Klamath; INW: interior northwest; NR: northern Rockies; SRSW: southern Rockies and AZ/NM mountains.

Supplemental tables

Table S1. Predictors included in final post-fire regeneration models, grouped by predictor type. The “Models” column indicates which final models each predictor was included in. Each model included only one climatology predictor and one or two post-fire climate variables (see methods for model selection procedure). Post-fire climate variables were annual anomalies (z-scores) and the most extreme value from any one year in the first five post-fire years were used (both maximum and minimum extremes). All non-climate variables were included in all models, except variety/subspecies which was only included for species with varieties or subspecies (Table S5). “All” is the all-species model; “PIPO/PIJE” is *Pinus ponderosa/P. jeffreyi*; “PSME” is *Pseudotsuga menziesii*; “ABCO/ABGR” is *Abies concolor/A. grandis*; “PICO” is *Pinus contorta*; “PIEN” is *Picea engelmannii*; “ABLA” is *Abies lasiocarpa*.

**Predictor type**	**Predictors**	**Models**
Climatology	30-year (1981-2010) mean annual climatic water deficit	All, ABCO/ABGR, PIEN, PSME, PIPO/PIJE
	30-year (1981-2010) mean growing season (April-September) climatic water deficit	ABLA, PICO
Post-fire climate	Growing season (April-September) climatic water deficit (max in post-fire years 1-5)	All, ABLA, PICO, PIPO/PIJE, PSME
	Growing season (April-September) climatic water deficit (min in post-fire years 1-5)	All, PIEN, PSME
	Summer (June-August) vapor pressure deficit (VPD; min in post-fire years 1-5)	ABCO/ABGR
	Growing season precipitation (April-September; max in post-fire years 1-5)	PIPO/PIJE, PICO
Seed availability and fire severity	Distance to seed source	All models
	Surrounding tree cover (within 300 m radius of plot center)	All models
	Satellite-derived fire severity (relativized burn ratio; RBR)	All models
Site characteristics	Pre-fire disturbance type (none, fire, beetle, blowdown, fuel treatment)	All models
	Time since fire	All models
	Continuous Heat Insolation Load Index (Chili; (18))	All models
Other	Plot size (offset)	All models
	Fire event (random effect)	All models
Variety/subspecies	Recognized variety or subspecies (Table S5)	ABCO/ABGR, PICO, PIPO/PIJE, PSME

Table S2. Five GCMs used to represent future climate conditions that represent a range of future scenarios for the US.

Model Name	Climate model institution
MRI-CGCM3	Meterorological Research Institute, Japan
HadGEM2-ES	Met Office Hadley Centre, United Kingdom
IPSL-CM5A-MR	Institut Pierre Simon Laplace, France
CNRM-CM5	National Centre of Meteorological Research, France
NorESM1-M	Norwegian Climate Center, Norway

Table S3. Correlation between post-fire climate conditions used as predictors in models. No two variables with correlations greater than 0.60 were used in a single model. “GS” is growing season (April-September). Deficit refers to climatic water deficit. “Precip.” is precipitation. “VPD” is vapor pressure deficit. Summer is the months June through August.

	Max GS deficit	Min GS deficit	Max GS precip.	Min GS precip.	Max summer VPD	Min summer VPD
Max GS deficit	1.00					
Min GS deficit	0.24	1.00				
Max GS precip.	-0.06	-0.61	1.00			
Min GS precip.	-0.63	-0.21	0.16	1.00		
Max summer VPD	0.69	0.29	-0.16	-0.33	1.00	
Min summer VPD	0.46	0.55	-0.28	-0.33	0.45	1.00

Table S4. Description of the low- and high-severity scenarios used in model projections.

Variable	Low severity	High severity
Distance to seed source	10 m	150 m
RBR	100	400
Surrounding tree cover	30%	10%

Table S5. Varieties and subspecies for study species.

Species	Varieties/subspecies included	Citations
Abies concolor/Abies grandis	4 (see map in Ott et al. 2015)	Ott et al 2015 (8)
Abies lasiocarpa	2 (var. *lasiocarpa* & var. *bifolia*); not included in final model because no difference in regeneration between varieties	Adams et al. 2011 (19); Hunt and von Rudloff. 1979 (20)
Pinus contorta	2 (plots only sampled spp. *lasiocarpa* and spp. *murrayana*)	Bisbing et al. 2020 (21)
Pinus ponderosa	2 (var. *ponderosa* & var. *scopularum*)	Potter et al. 2015 (22)
Pseudotsuga menziesii	2 (var. *menziesii* & var. *glauca*)	Aagaard et al. 1995 (23); Gugger 2010 (24); Li &Adams 1989 (25)

Table S6. Number of plots in the all-species and individual species models by pre-fire disturbance type. "ABCO/ABGR" is *Abies concolor/A. grandis*; "ABLA" is *Abies lasiocarpa;* "PIEN" is *Picea engelmannii*; "PICO" is *Pinus contorta*; "PIPO/PIJE" is *Pinus ponderosa/P. jeffreyi*; "PSME" is *Pseudotsuga menziesii*.

Type	All	ABCO/ABGR	ABLA	PIEN	PICO	PIPO/PIJE	PSME
Blowdown	106	0	101	53	91	0	6
Douglas-fir beetle	72	41	16	3	40	64	71
Fuel treatment	186	151	0	0	14	186	61
None	9123	3577	1806	1241	2634	7229	5400
Pine beetle	185	0	37	10	120	0	87
Prescribed fire	12	12	0	0	10	12	0
Spruce beetle	80	8	80	75	9	1	32
Wildfire	466	57	230	141	341	227	361

Table S7. Accuracy of raster data projecting presence/absence of species compared to data from FIA plots from (7) and to field data from this study (Sensitivity, Balanced accuracy). “ABLA” is *Abies lasiocarpa*; “ABCO” is *Abies concolor*; “ABGR” is *Abies grandis*; “PICO” is *Pinus contorta*; “PIEN” is *Picea engelmannii*; “PIPO/PIJE” is *Pinus ponderosa/P. jeffreyi*; “PSME” is *Pseudotsuga menziesii*.

Species	FIA sensitivity	Sensitivity	Balanced accuracy
ABLA	0.88	0.81	0.74
ABCO	0.88	0.98	0.67
ABGR	0.87	0.95	0.80
PICO	0.92	0.76	0.73
PIEN	0.88	0.85	0.69
PIPO/PIJE	0.93	0.63	0.76
PSME	0.96	0.70	0.68

Model performance and output

Table S8. Model performance metrics for each species and the all-species models. “AUC” refers to the area under the receiver operating characteristic curve. “CV AUC” is the mean AUC from 10-fold cross validation (predictions made to data not used to build the model). “Threshold^{ss}” refers to the probability threshold used to categorize regeneration as likely or unlikely which maximizes the sum of specificity and sensitivity (26). “Threshold^{k}” refers to the probability threshold used to categorize regeneration as likely or unlikely which maximizes Cohen’s kappa (27).

Species	Plots	Fires	AUC	CV AUC	Threshold^{ss}	Threshold^{k}
Pinus ponderosa/P. jeffreyi	7719	276	0.70	0.69	0.22	0.28
Pseudotsuga menziesii	6018	274	0.74	0.75	0.39	0.44
Abies concolor/A. grandis	3846	192	0.73	0.71	0.35	0.36
Pinus contorta	3232	178	0.79	0.77	0.59	0.46
Picea engelmannii	1520	136	0.73	0.71	0.50	0.50
Abies lasiocarpa	2268	139	0.81	0.78	0.16	0.37
All species	10230	334	0.75	0.74	0.64	0.54

Table S9. Coefficient estimates, standard errors (SE), z-values, and p-values from the generalized linear mixed effect model for all species combined. "CHILI" is Continuous Heat Insolation Load Index (18). "GS" is growing season (April-September). The reference level for pre-fire disturbance was "none."

Predictor	Estimate	SE	z-value	p-value
Intercept	-1.9120	0.6480	-2.95	0.003
Time since fire	0.0401	0.0132	3.03	0.002
Pre-fire disturbance beetle	-0.4355	0.4291	-1.02	0.310
Pre-fire disturbance blowdown	-0.6983	0.5063	-1.38	0.168
Pre-fire disturbance fuel treatment	0.5522	0.2166	2.55	0.011
Pre-fire disturbance wildfire	0.0557	0.2577	0.22	0.829
CHILI	-46.8900	3.5820	-13.09	<0.001
$CHILI^2$	-7.9360	3.4740	-2.29	0.022
Distance seed source	-0.0048	0.0004	-12.13	<0.001
Fire severity (RBR)	0.0014	0.0006	2.34	0.019
Surrounding tree cover	52.1200	4.4590	11.69	<0.001
Surrounding tree $cover^2$	-13.2700	3.2350	-4.10	<0.001
30-yr annual climatic water deficit	-0.0031	0.0010	-3.20	0.001
Max post-fire GS climatic water deficit	0.7348	0.3641	2.02	0.044
Min post-fire GS climatic water deficit	-0.2648	0.1454	-1.82	0.069
30-yr annual climatic water deficit *max post-fire GS climatic water deficit	-0.0018	0.0006	-2.89	0.004
RBR*max post-fire GS climatic water deficit	-0.0010	0.0004	-2.77	0.006

Table S10. Coefficient estimates, standard errors (SE), z-values, and p-values from the generalized linear mixed effect model for *Pinus ponderosa/P. jeffreyi*. “CHILI” is Continuous Heat Insolation Load Index (18). “GS” is growing season (April-September). The reference level for pre-fire disturbance was “none.” The reference level for variety was var. *scopulorum*.

Predictor	Estimate	SE	z-value	p-value
Intercept	-4.2250	0.5913	-7.15	<0.001
Variety (*ponderosa*)	0.1057	0.2840	0.37	0.710
Time since fire	0.0073	0.0129	0.57	0.572
Pre-fire disturbance fuel treatment	0.8169	0.2591	3.15	0.002
Pre-fire disturbance other beetle	0.1770	0.5530	0.32	0.749
Pre-fire disturbance wildfire	-0.4621	0.3020	-1.53	0.126
CHILI	-0.0010	0.0011	-0.94	0.346
Distance seed source	-0.0061	0.0005	-12.25	<0.001
Fire severity (RBR)	0.0038	0.0007	5.26	<0.001
Surrounding tree cover	48.7800	7.2780	6.70	<0.001
Surrounding tree cover^2	-7.5590	5.7160	-1.32	0.186
30-yr annual climatic water deficit	-0.0016	0.0005	-3.49	<0.001
Max post-fire GS climatic water deficit	-0.2960	0.1646	-1.80	0.072
Max post-fire GS precip.	3.4080	7.3940	0.46	0.645
Max post-fire GS precip. ^{2}	-25.5800	6.9930	-3.66	<0.001
Variety*surrounding tree cover	-34.5100	9.1790	-3.76	<0.001
Variety*surrounding tree cover^2	-12.6800	7.1390	-1.78	0.076
Variety*RBR	-0.0016	0.0006	-2.80	0.005
RBR*max post-fire GS climatic water deficit	-0.0010	0.0004	-2.45	0.014

Table S11. Coefficient estimates, standard errors (SE), z-values, and p-values from the generalized linear mixed effect model for *Pseudotsuga menziesii*. “CHILI” is Continuous Heat Insolation Load Index (18). “GS” is growing season (April-September). The reference level for pre-fire disturbance was “none.” The reference level for variety was var. *glauca*.

Predictor	Estimate	SE	z-value	p-value
Intercept	-2.7910	0.5906	-4.73	<0.001
Variety (*menziesii*)	1.1330	0.2953	3.84	<0.001
Time since fire	0.0057	0.0162	0.35	0.723
Pre-fire disturbance Doug-fir beetle	0.0262	0.6230	0.04	0.966
Pre-fire disturbance fuel treatment	-0.5472	0.4475	-1.22	0.221
Pre-fire disturbance other beetle	-3.4010	1.0620	-3.20	0.001
Pre-fire disturbance wildfire	-0.5534	0.2307	-2.40	0.016
CHILI	-50.2900	3.3590	-14.97	<0.001
$CHILI^2$	-8.1890	3.2090	-2.55	0.011
Distance seed source	-0.0063	0.0006	-10.35	<0.001
Fire severity (RBR)	0.0013	0.0010	1.37	0.171
Surrounding tree cover	0.0402	0.0049	8.19	<0.001
30-yr annual climatic water deficit	-0.0054	0.0006	-9.77	<0.001
Min post-fire GS climatic water deficit	0.1781	0.2244	0.79	0.427
Max post-fire GS climatic water deficit	0.2077	0.1971	1.05	0.292
RBR*min post-fire GS climatic water deficit	-0.0019	0.0005	-3.90	<0.001
RBR*max post-fire GS climatic water deficit	-0.0030	0.0005	-5.93	<0.001

Table S12. Coefficient estimates, standard errors (SE), z-values, and p-values from the generalized linear mixed effect model for *Abies concolor/A. grandis*. “CHILI” is Continuous Heat Insolation Load Index (18). “GS” is growing season (April-September). The reference level for pre-fire disturbance was “none.” The reference level for variety was pure *A. concolor* (Con). Other values for variety include pure *A. grandis* (Gra), a hybrid of the two species (Hyb), and *A. concolor* var. *lowiana* (Low; see supplemental methods).

Predictor	Estimate	SE	z-value	p-value
Intercept	-6.1460	0.8139	-7.55	<0.001
Variety (*grandis*)	1.7400	0.5801	3.00	0.003
Variety (hybrid)	1.8360	0.5369	3.42	<0.001
Variety (*lowiana*)	2.1830	0.4663	4.68	<0.001
Time since fire	0.0049	0.0209	0.23	0.815
Pre-fire disturbance fuel treatment	0.4059	0.2816	1.44	0.150
Pre-fire disturbance other beetle	-1.2720	0.7243	-1.76	0.079
Pre-fire disturbance wildfire	-3.4720	0.6599	-5.26	<0.001
CHILI	-35.1900	3.4600	-10.17	<0.001
$CHILI^2$	-14.0400	3.2000	-4.39	<0.001
Distance seed source	-0.0045	0.0007	-6.17	<0.001
Fire severity (RBR)	-0.0016	0.0004	-3.79	<0.001
Surrounding tree cover	0.0552	0.0074	7.42	<0.001
30-yr annual climatic water deficit	-0.0034	0.0010	-3.56	<0.001
Min post-fire summer VPD	-1.3760	0.3040	-4.53	<0.001
Tree cover*min post-fire summer VPD	0.0367	0.0084	4.38	<0.001

Table S13. Coefficient estimates, standard errors (SE), z-values, and p-values from the generalized linear mixed effect model for *Pinus contorta*. "CHILI" is Continuous Heat Insolation Load Index (18). "GS" is growing season (April-September). The reference level for pre-fire disturbance was "none." The "other" group included beetles that do not affect lodgepole pine (49 plots) and fuel treatments (24 plots) due to small numbers of plots in both categories. The reference level for subspecies was spp. *latifolia*.

Predictor	Estimate	SE	z-value	p-value
Intercept	-3.6550	0.6004	-6.09	<0.001
Subspecies (*murrayana*)	-0.9568	0.6254	-1.53	0.126
Time since fire	0.0486	0.0223	2.18	0.029
Pre-fire disturbance blowdown	-1.1600	0.5002	-2.32	0.020
Pre-fire disturbance other	-0.5979	0.5833	-1.03	0.305
Pre-fire disturbance pine beetle	0.5309	1.6350	0.33	0.745
Pre-fire disturbance wildfire	0.1247	0.2985	0.42	0.676
CHILI	-35.5500	3.7440	-9.50	<0.001
$CHILI^2$	-14.1000	3.3460	-4.22	<0.001
Distance seed source	-0.0031	0.0005	-6.42	<0.001
Fire severity (RBR)	0.0025	0.0007	3.49	<0.001
Surrounding tree cover	0.0140	0.0088	1.59	0.112
30-yr GS climatic water deficit	-0.0056	0.0011	-5.03	<0.001
Max post-fire GS climatic water deficit	-2.3880	8.7990	-0.27	0.786
Max post-fire GS climatic water deficit ^{2}	-39.2000	6.9010	-5.68	<0.001
Max post-fire GS precipitation	0.1727	0.2664	0.65	0.517
RBR*subspecies (*murrayana*)	-0.0046	0.0013	-3.63	<0.001
RBR*max post-fire GS precipitation	0.0023	0.0007	3.45	<0.001

Table S14. Coefficient estimates, standard errors (SE), z-values, and p-values from the generalized linear mixed effect model for *Picea engelmannii*. “CHILI” is Continuous Heat Insolation Load Index (18). “GS” is growing season (April-September). The reference level for pre-fire disturbance was “none.” “Other” refers to beetles affecting other tree species (13 plots) and blowdown (50 plots).

Predictor	Estimate	SE	z-value	p-value
Intercept	-2.9114	0.7415	-3.93	<0.001
Time since fire	0.0289	0.0299	0.97	0.334
Pre-fire disturbance other	-2.5146	0.8682	-2.90	0.004
Pre-fire disturbance spruce beetle	-2.0939	0.6725	-3.11	0.002
Pre-fire disturbance wildfire	0.5583	0.3759	1.49	0.137
CHILI	-33.8008	3.8007	-8.89	<0.001
$CHILI^2$	-10.4936	3.4504	-3.04	0.002
Distance seed source	-0.0031	0.0006	-4.98	<0.001
Fire severity (RBR)	-8.1633	3.6775	-2.22	0.026
Fire severity $(RBR)^2$	-7.8182	3.1053	-2.52	0.012
Surrounding tree cover	0.0414	0.0118	3.49	<0.001
30-yr annual climatic water deficit	-0.0048	0.0014	-3.36	<0.001
Min post-fire GS climatic water deficit	-13.1105	8.0693	-1.63	0.104
Min post-fire GS climatic water $deficit^2$	-17.6632	5.3333	-3.31	<0.001

Table S15. Coefficient estimates, standard errors (SE), z-values, and p-values from the generalized linear mixed effect model for *Abies lasiocarpa*. “CHILI” is Continuous Heat Insolation Load Index (18). “GS” is growing season (April-September). The reference level for pre-fire disturbance was “none.”

Predictor	Estimate	SE	z-value	p-value
Intercept	-6.3520	0.6635	-9.57	<0.001
Time since fire	0.1643	0.0250	6.58	<0.001
Pre-fire disturbance blowdown	-1.9670	0.5441	-3.62	<0.001
Pre-fire disturbance other beetle	-1.4190	0.5736	-2.47	0.013
Pre-fire disturbance wildfire	-0.2662	0.3285	-0.81	0.418
CHILI	-30.3100	3.5580	-8.52	<0.001
$CHILI^2$	-9.2480	3.6740	-2.52	0.012
Distance seed source	-0.0070	0.0009	-7.60	<0.001
Fire severity (RBR)	-0.0011	0.0005	-2.12	0.034
Surrounding tree cover	0.0364	0.0104	3.51	<0.001
30-yr GS climatic water deficit	-0.0055	0.0015	-3.67	<0.001
Max post-fire GS climatic water deficit	2.8740	12.4900	0.23	0.818
Max post-fire GS climatic water deficit ^{2}	-55.5900	12.9500	-4.29	<0.001
Surrounding tree cover*max post-fire GS climatic water deficit	-0.3675	0.4549	-0.81	0.419
Surrounding tree cover*max post-fire GS climatic water deficit ^{2}	1.7420	0.4744	3.67	<0.001

Model projections under different climate and fire severity scenarios

Table S16. Median and standard deviation (SD) of recruitment probability projected by the all-species model across the range of the study species under different climate and fire scenarios.

Years	Severity scenario	Median recruitment probability	SD
1981-2000	High	0.70	0.22
1981-2000	Low	0.92	0.14
2001-2020	High	0.53	0.27
2001-2020	Low	0.86	0.23
2031-2050 RCP 4.5	High	0.40	0.26
2031-2050 RCP 4.5	Low	0.80	0.26
2031-2050 RCP 8.5	High	0.34	0.24
2031-2050 RCP 8.5	Low	0.75	0.25

Table S17. Difference (Dif.) in the median recruitment probability projected by the all-species model across the range of the study species under different climate and fire scenarios. Positive values indicate higher median recruitment probability under the scenario in “Comparison group 1” than “Comparison group 2.”

Effect	Constant	Comparison group 1	Comparison group 2	Dif. in median recruitment probability
Climate	High-severity scenario	1980-2000	2001-2020	0.166
Climate	Low-severity scenario	1980-2000	2001-2020	0.053
Climate	High-severity scenario	2001-2020	2031-2050 RCP 4.5	0.131
Climate	Low-severity scenario	2001-2020	2031-2050 RCP 4.5	0.069
Climate	High-severity scenario	2001-2020	2031-2050 RCP 8.5	0.191
Climate	Low-severity scenario	2001-2020	2031-2050 RCP 8.5	0.114
Severity	1981-2000	Low-severity scenario	High-severity scenario	0.217
Severity	2001-2020	Low-severity scenario	High-severity scenario	0.330
Severity	2031-2050 RCP 4.5	Low-severity scenario	High-severity scenario	0.392
Severity	2031-2050 RCP 8.5	Low-severity scenario	High-severity scenario	0.406

Table S18. Proportion of study area (Fig. 1) where recruitment is unlikely under both fire-severity scenarios, likely under only the low severity scenario, or likely under both severity scenarios for each time period and future climate scenario. We used the probability threshold that maximizes kappa (0.54) to determine where recruitment is “likely” or “unlikely” (see methods). See Figs. 2 and S10 for spatial distribution of these groups.

	1981-2000	2001-2020	2031-2050 RCP 4.5	2031-2050 RCP 8.5
Unlikely either severity	0.05	0.18	0.26	0.31
Likely low severity only	0.26	0.35	0.40	0.42
Likely both severities	0.69	0.47	0.34	0.27

References

1. B. W. Allred *et al.*, Improving Landsat predictions of rangeland fractional cover with multitask learning and uncertainty. *Methods in Ecology and Evolution* **12**, 841-849 (2021).
2. J. D. Coop *et al.*, Contributions of fire refugia to resilient ponderosa pine and dry mixed-conifer forest landscapes. *Ecosphere* **10**, e02809 (2019).
3. D. C. Donato *et al.*, Conifer regeneration in stand-replacement portions of a large mixed-severity wildfire in the Klamath-Siskiyou Mountains. *Canadian Journal of Forest Research* **39**, 823-838 (2009).
4. N. A. Povak *et al.*, Wildfire severity and postfire salvage harvest effects on long-term forest regeneration. *Ecosphere* **11** (2020).
5. A. J. Tepley, J. R. Thompson, H. E. Epstein, K. J. Anderson-Teixeira, Vulnerability to forest loss through altered postfire recovery dynamics in a warming climate in the Klamath Mountains. *Global Change Biology* **23**, 4117-4132 (2017).
6. N. S. Gill *et al.*, Limitations to Propagule Dispersal Will Constrain Postfire Recovery of Plants and Fungi in Western Coniferous Forests. *BioScience*, biab139 (2022).
7. J. R. Ellenwood, F. J. Krist, S. A. Romero (2015) National individual tree species atlas. in *FHTET-15-01* (U.S. Department of Agriculture, Forest Service, Forest Health Technology Enterprise Team, Fort Collins).
8. T. M. Ott, E. K. Strand, C. L. Anderson, Niche divergence of *Abies grandis-Abies concolor* hybrids. *Plant Ecology* **216**, 479-490 (2015).
9. B. M. Collins, G. B. Roller, Early forest dynamics in stand-replacing fire patches in the northern Sierra Nevada, California, USA. *Landscape Ecology* **28**, 1801-1813 (2013).
10. D. J. N. Young *et al.*, Post-fire forest regeneration shows limited climate tracking and potential for drought-induced type conversion. *Ecology* **100** (2019).
11. S. A. Parks, L. M. Holsinger, M. A. Voss, R. A. Loehman, N. P. Robinson, Mean Composite Fire Severity Metrics Computed with Google Earth Engine Offer Improved Accuracy and Expanded Mapping Potential. *Remote Sensing* **10** (2018).
12. J. T. Stevens, B. M. Collins, J. D. Miller, M. P. North, S. L. Stephens, Changing spatial patterns of stand-replacing fire in California conifer forests. *Forest Ecology and Management* **406**, 28-36 (2017).
13. C. A. Cansler, D. McKenzie, Climate, fire size, and biophysical setting control fire severity and spatial pattern in the northern Cascade Range, USA. *Ecological Applications* **24**, 1037-1056 (2014).
14. B. J. Harvey, D. C. Donato, M. G. Turner, Drivers and trends in landscape patterns of stand-replacing fire in forests of the US Northern Rocky Mountains (1984-2010). *Landscape Ecology* **31**, 2367-2383 (2016).
15. C. S. Stevens-Rumann, P. Morgan, Tree regeneration following wildfires in the western US: a review. *Fire Ecology* **15**, 15 (2019).
16. M. P. Singleton, A. E. Thode, A. J. S. Meador, J. M. Iniguez, J. T. Stevens, Management strategy influences landscape patterns of high-severity burn patches in the southwestern United States. *Landscape Ecology* **36**, 3429-3449 (2021).
17. S. A. Parks, J. T. Abatzoglou, Warmer and Drier Fire Seasons Contribute to Increases in Area Burned at High Severity in Western US Forests From 1985 to 2017. *Geophysical Research Letters* **47**, e2020GL089858 (2020).
18. D. M. Theobald, D. Harrison-Atlas, W. B. Monahan, C. M. Albano, Ecologically-

Relevant Maps of Landforms and Physiographic Diversity for Climate Adaptation Planning. *Plos One* **10** (2015).
19. R. P. Adams, C. J. Earle, D. Thornburg, Taxonomy of infraspecifc taxa of Abies lasiocarpa: leaf essential oils and DNA of Abies lasiocarpa, var. bifolia and var. arizonica. *Phytologia* **93**, 73-87 (2011).
20. R. S. Hunt, E. V. Rudloff, Chemosystematic studies in the genus *Abies* .4. Introgression in *Abies lasiocarpa* and *Abies bifolia*. *Taxon* **28**, 297-305 (1979).
21. S. M. Bisbing *et al.*, Can long-lived species keep pace with climate change? Evidence of local persistence potential in a widespread conifer. *Diversity and Distributions* **27**, 296-312 (2021).
22. K. M. Potter, V. D. Hipkins, M. F. Mahalovich, R. E. Means, Nuclear genetic variation across the range of ponderosa pine (*Pinus ponderosa*): Phylogeographic, taxonomic and conservation implications. *Tree Genetics & Genomes* **11**, 38 (2015).
23. J. E. Aagaard, S. S. Vollmer, F. C. Sorensen, S. H. Strauss, Mitochondrial DNA products among RAPD profiles are frequent and strongly differentiated between races of Douglas-fir. *Molecular Ecology* **4**, 441-446 (1995).
24. P. F. Gugger, S. Sugita, J. Cavender-Bares, Phylogeography of Douglas-fir based on mitochondrial and chloroplast DNA sequences: testing hypotheses from the fossil record. *Molecular Ecology* **19**, 1877-1897 (2010).
25. P. Li, W. T. Adams, Range-wide patterns of allozyme variation in Douglas-fir (*Pseudotsuga menziesii*). *Canadian Journal of Forest Research* **19**, 149-161 (1989).
26. C. R. Liu, P. M. Berry, T. P. Dawson, R. G. Pearson, Selecting thresholds of occurrence in the prediction of species distributions. *Ecography* **28**, 385-393 (2005).
27. E. A. Freeman, G. G. Moisen, A comparison of the performance of threshold criteria for binary classification in terms of predicted prevalence and kappa. *Ecological Modelling* **217**, 48-58 (2008).
