# Supplementary material for: Reduced fire severity offers near-term buffer to climate-driven declines in conifer resilience across the western United States
Source: Proc Natl Acad Sci U S A. 2023 Mar 6;120(11):e2208120120. doi: 10.1073/pnas.2208120120 (PMC10089158; doi:10.1073/pnas.2208120120)
Supplement: Supplementary file 2 — Dataset S01 (PDF) [file pnas.2208120120.sd01.pdf]

PNAS

Supplementary Dataset for

Reduced fire severity offers near-term buffer to climate-driven declines in conifer resilience across the western United States

Kimberley T. Davis, Marcos D. Robles, Kerry B. Kemp, Philip E. Higuera, Teresa Chapman, Kerry L. Metlen, Jamie L. Peeler, Kyle C Rodman, Travis Woolley, Robert N. Addington, Brian J. Buma, C. Alina Cansler, Michael J. Case, Brandon M. Collins, Jonathan D. Coop, Solomon Z. Dobrowski, Nathan S Gill, Collin Haffey, Lucas B. Harris, Brian J. Harvey, Ryan D. Haugo, Matthew D. Hurteau, Dominik Kulakowski, Caitlin E. Littlefield, Lisa McCauley, Nicholas Povak, Kristen L. Shive, Edward Smith, Jens T. Stevens, Camille S. Stevens-Rumann, Alan H. Taylor, Alan J. Tepley, Derek J. N. Young, Robert A. Andrus, Mike A. Battaglia, Julia K. Berkey, Sebastian U. Busby, Amanda Carlson, Marin Chambers, Erich Kyle Dodson, Daniel C. Donato, William M. Downing, Paula J. Fornwalt, Joshua S. Halofsky, Ashley Hoffman, Andres Holz, Jose M. Iniguez, Meg A. Krawchuk, Mark R. Kreider, Andrew J. Larson, Garrett W. Meigs, John Paul Roccaforte, Monica T. Rother, Hugh Safford, Michael Schaedel, Jason Sibold, Megan Singleton, Monica G. Turner, Alexandra K. Urza, Kyra D. Clark-Wolf, Larissa Yocom, Joseph B. Fontaine, John Campbell

Corresponding author: Kimberley T. Davis
Email: Kimberley.Davis@usda.gov

Dataset S1. Data contributors, number of plots, and related publication and data citations. Most datatsets involved multiple co-authors and the contributor column lists only the corresponding contributors, see citations for additional co-authors of each dataset.

Contributor	Plots	Manuscript and data citation
Andrus, R. A.	43	Andrus, R. A., S. J. Hart, N. Tutland, and T. T. Veblen. 2021. Future dominance by quaking aspen expected following short-interval, compounded disturbance interaction. Ecosphere 12:e03345. https://doi.org/10.1002/ecs2.3345
Berkey, J.K. & Larson, A.J.	209	Berkey, J. K., R. T. Belote, C. T. Maher, and A. J. Larson. 2021. Structural diversity and development in active fire regime mixed-conifer forests. Forest Ecology and Management 479:118548. https://doi.org/10.1016/j.foreco.2020.118548<br>**Data:** Larson, Andrew J.; Belote, R. Travis; Maher, Colin T.; Berkey, Julia K. 2019. Forest structure, regeneration, and fuels in unburned, once-burned and twice-burned mixed-conifer forests of the Bob Marshall Wilderness. Fort Collins, CO: Forest Service Research Data Archive. https://doi.org/10.2737/RDS-2019-0004
Boag, A.	176	Boag, A. E., M. J. Ducey, M. W. Palace, and J. Hartter. 2020. Topography and fire legacies drive variable post-fire juvenile conifer regeneration in eastern Oregon, USA. Forest Ecology and Management 474:118312. https://doi.org/10.1016/j.foreco.2020.118312
Buma, B.	111	Buma, B., and C. A. Wessman. (2011). Disturbance interactions can impact resilience mechanisms of forests. Ecosphere 2:1-13. https://doi.org/10.1890/ES11-00038.1
Busby, S. U.	373	Busby, S. U., K. B. Moffett, and S. Holz. 2020. High-severity and short-interval wildfires limit forest recovery in the Central Cascade Range. Ecosphere 11:22. https://doi.org/10.1002/ecs2.3247<br>Busby, S.U. and A. Holz. 2022. Interactions between fire refugia and climate-environment conditions determine mesic subalpine forest recovery after large and severe wildfires. Frontiers in Forests and Global Change 5: 890893. https://doi.org/10.3389/ffgc.2022.890893
Cansler, C. A.	76	Cansler, C.A., D. McKenzie, and C. B. Halpern. 2018. Fire enhances the complexity of forest structure in alpine treeline ecotones. Ecosphere, 9:e02091. https://doi.org/10.1002/ecs2.2091<br>Cansler, C.A., 2015. Multi-scale analysis of fire effects in alpine treeline ecotones (Doctoral dissertation; University of Washington).
Carlson, A. R.	26	Carlson, A. R., J. S. Sibold, and J. F. Negrón. (2020). Canopy structure and below-canopy temperatures interact to shape seedling response to disturbance in a Rocky Mountain subalpine forest. Forest Ecology and Management 472:118234. https://doi.org/10.1016/j.foreco.2020.118234<br>Carlson, A. R., J. S. Sibold, and J. F. Negrón. (2020). Wildfire and spruce beetle outbreak have mixed effects on below-canopy temperatures in a Rocky Mountain subalpine forest. Journal of Biogeography 48:216-230. https://doi.org/10.1111/jbi.13994
Chapman, T. B.	64	Chapman, T. B., T. L. Schoennagel, T. T. Veblen, and K. C. Rodman. In prep. A Closed Cone is a Terrible Thing to Lose: Serotiny and Regeneration of Lodgepole Pine Following Fire and Beetle Disturbance in Colorado and Wyoming, USA.
Clark-Wolf, K. D.	49	Clark-Wolf, K. D., P. E. Higuera, and K. T. Davis. 2022. Conifer seedling demography reveals mechanisms of initial forest resilience to wildfires in the northern Rocky Mountains. Forest Ecology and Management 523:120487. https://doi.org/10.1016/j.foreco.2022.120487<br>**Data:** Clark-Wolf, K. 2022. Conifer seedling demography reveals mechanisms of initial forest resilience to wildfires in the northern Rocky Mountains. Dryad, Dataset, https://doi.org/10.5061/dryad.9s4mw6mhj
Collins, B. M.	151	Collins, B. M., and G. B. Roller. 2013. Early forest dynamics in stand-replacing fire patches in the northern Sierra Nevada, California, USA. Landscape Ecology 28:1801-1813. https://doi.org/10.1007/s10980-013-9923-8

Contributor	Plots	Manuscript and data citation
Coop, J. D.	497	Coop, J. D., T. J. DeLory, W. M. Downing, S. L. Haire, M. A. Krawchuk, C. Miller, M.-A. Parisien, and R. B. Walker. 2019. Contributions of fire refugia to resilient ponderosa pine and dry mixed-conifer forest landscapes. Ecosphere 10:e02809. https://doi.org/10.1002/ecs2.2809<br>**Data:** Miller, C., Krawchuk, M. A., Coop, J. D., Downing, W. M., Walker, R. B., Haire, S. L., Chong, G., Whitman, E., Parisien, M. 2020. Field and spatial data from study areas in western U.S. for: Understanding the role of fire refugia in promoting ecosystem resilience of dry forests in the western United States. Fort Collins, CO: Forest Service Research Data Archive. https://doi.org/10.2737/RDS-2021-0003
Davis, K. T.	89	Davis, K. T., S. Z. Dobrowski, P. E. Higuera, Z. A. Holden, T. T. Veblen, M. T. Rother, S. A. Parks, A. Sala, and M. P. Maneta. 2019. Wildfires and climate change push low-elevation forests across a critical climate threshold for tree regeneration. Proceedings of the National Academy of Sciences of the United States of America 116:6193-6198. https://doi.org/10.1073/pnas.1815107116<br>Davis, K. T., P. E. Higuera, S. Dobrowski, S. Parks, J. T. Abatzoglou, M. Rother, and T. Veblen. 2020. Fire-catalyzed vegetation shifts in ponderosa pine and Douglas-fir forests of the western United States. Environmental Research Letters 15:1040b8. https://doi.org/10.1088/1748-9326/abb9df<br>**Data:** Davis, K. T. et al. (2021), Wildfires and climate change push low-elevation forests across a critical climate threshold for tree regeneration, Dryad, Dataset, https://doi.org/10.5061/dryad.pc3f9d8
Dobrowski, S. Z.	143	Carlson, C. H., Dobrowski, S. Z., and Safford, H. D. 2012. Variation in tree mortality and regeneration affect forest carbon recovery following fuel treatments and wildfire in the Lake Tahoe Basin, California, USA. Carbon balance and management 7.
Dodson, E. K.	104	Dodson, E. K., and H. T. Root. 2013. Conifer regeneration following stand-replacing wildfire varies along an elevation gradient in a ponderosa pine forest, Oregon, USA. Forest Ecology and Management 302:163-170. https://doi.org/10.1016/j.foreco.2013.03.050
Donato, D. C.	104	Campbell J.L., D.C. Donato, and J.B. Fontaine. 2016. Effects of post-fire logging on fuel dynamics in a mixed-conifer forest, Oregon, USA: A ten-year assessment. International Journal of Wildland Fire 25(6): 646-656. https://doi.org/10.1071/WF15119<br>Donato D.C., J.B. Fontaine, W.D. Robinson, J.B. Kauffman, and B.E. Law. 2009. Vegetation response to a short interval between high-severity wildfires in a mixed-evergreen forest. Journal of Ecology 97: 142-154. https://doi.org/10.1111/j.1365-2745.2008.01456.x<br>Donato D.C., J.B. Fontaine, J.L. Campbell, W.D. Robinson, J.B. Kauffman, and B.E. Law. 2009. Conifer regeneration in stand-replacement portions of a large mixed-severity wildfire in the Klamath-Siskiyou Mountains. Canadian Journal of Forest Research 39: 823-838. https://doi.org/10.1139/X09-016<br>Donato D.C., J.B. Fontaine, J.L. Campbell, W.D. Robinson, J.B. Kauffman, and B.E. Law. 2006. Post-wildfire logging hinders regeneration and increases fire risk. Science 311: 352. https://doi.org/10.1126/science.1122855<br>Donato DC, BJ Harvey, MG Turner. 2016. Regeneration of montane forests 24 years after the 1988 Yellowstone fires: A fire-catalyzed shift in lower treelines? Ecosphere 7:e01410. https://doi.org/10.1002/ecs2.1410<br>Stevens-Rumann, C. S., K. B. Kemp, P. E. Higuera, B. J. Harvey, M. T. Rother, D. C. Donato, P. Morgan, and T. T. Veblen. 2018. Evidence for declining forest resilience to wildfires under climate change. Ecology Letters 21:243-252. https://doi.org/10.1111/ele.12889
Donato, D. C. & Halofsky, J. S.	243	Data published as part of this manuscript: Davis, K. T. et al. 2023. Data from: Reduced fire severity offers near-term buffer to climate-driven declines in conifer resilience across the western United States. Dryad, Dataset, https://doi.org/10.5061/dryad.0rxwdbs47

Contributor	Plots	Manuscript and data citation
Downing, W. M.	186	Downing, W. M., M. A. Krawchuk, G. W. Meigs, S. L. Haire, J. D. Coop, R. B. Walker, E. Whitman, G. Chong, and C. Miller. 2019. Influence of fire refugia spatial pattern on post-fire forest recovery in Oregon's Blue Mountains. Landscape Ecology 34:771-792. https://doi.org/10.1007/s10980-019-00802-1<br>**Data:** Miller, C., Krawchuk, M. A., Coop, J. D., Downing, W. M., Walker, R. B., Haire, S. L., Chong, G., Whitman, E., Parisien, M. 2020. Field and spatial data from study areas in western U.S. for: Understanding the role of fire refugia in promoting ecosystem resilience of dry forests in the western United States. Fort Collins, CO: Forest Service Research Data Archive. https://doi.org/10.2737/RDS-2021-0003
Dunn, C. J.	51	Dunn, C. J., J. D. Johnston, M. J. Reilly, J. D. Bailey, and R. A. Miller. 2020. How does tree regeneration respond to mixed-severity fire in the western Oregon Cascades, USA? Ecosphere 11:e03003. https://doi.org/10.1002/ecs2.3003
Fornwalt, P. J.	753	Chambers M.E., P.J. Fornwalt, S.L. Malone, and M.A. Battaglia. 2016. Patterns of conifer regeneration following high severity wildfire in ponderosa pine–dominated forests of the Colorado Front Range. Forest Ecology and Management 378:57-67. https://doi.org/10.1016/j.foreco.2016.07.001<br>**Data:** Fornwalt, Paula J.; Chambers, Marin E.; Battaglia, Mike A.; Iniguez, Jose M.; Sieg, Carolyn H. 2022. Post-fire tree regeneration in burned ponderosa pine forests of South Dakota, Wyoming, and Colorado, USA. Fort Collins, CO: Forest Service Research Data Archive. https://doi.org/10.2737/RDS-2022-0042
Gill, N. S.	37	Data published as part of this manuscript; co-contributor Durboraw, T.
Haffey, C.	197	Haffey, C., T. D. Sisk, C. D. Allen, A. E. Thode, and E. Q. Margolis. 2018. Limits to ponderosa pine regeneration following large high-severity forest fires in the United States southwest. Fire Ecology 14:143–163. https://doi.org/10.4996/fireecology.140114316
Harris, L. B.	464	Harris, L.B., Drury, S.A., Taylor, A.H., 2021. Strong legacy effects of prior burn severity on forest resilience to a high-severity fire. Ecosystems 24, 774–787. https://doi.org/10.1007/s10021-020-00548-x<br>Pierce AD, Taylor AH (2011) Fire severity and seed source influence lodgepole pine (*Pinus contorta* var. murrayana) regeneration in the southern cascades, Lassen Volcanic National Park, California. Landscape Ecology 26, 225–237. https://doi.org/10.1007/s10980-010-9556-0<br>Harris LB, Taylor AH (2020) Rain-shadow forest margins resilient to low-severity fire and climate change but not high-severity fire. Ecosphere 11, e03258. https://doi.org/10.1002/ecs2.3258
Harvey, B. J.	365	Harvey, B. J., D. C. Donato, W. H. Romme, and M. G. Turner. 2013. Influence of recent bark beetle outbreak on fire severity and postfire tree regeneration in montane Douglas-fir forests. Ecology 94:2475-2486. https://doi.org/10.1890/13-0188.1<br>Harvey, B. J., D. C. Donato, and M. G. Turner. 2016. High and dry: post-fire tree seedling establishment in subalpine forests decreases with post-fire drought and large stand-replacing burn patches. Global Ecology and Biogeography 25:655-669. https://doi.org/10.1111/geb.12443<br>Stevens-Rumann, C. S., K. B. Kemp, P. E. Higuera, B. J. Harvey, M. T. Rother, D. C. Donato, P. Morgan, and T. T. Veblen. 2018. Evidence for declining forest resilience to wildfires under climate change. Ecology Letters 21:243-252. https://doi.org/10.1111/ele.12889
Hurteau, M. D.	50	Data published as part of this manuscript: Davis, K. T. et al. 2023. Data from: Reduced fire severity offers near-term buffer to climate-driven declines in conifer resilience across the western United States. Dryad, Dataset, https://doi.org/10.5061/dryad.0rxwdbs47

Contributor	Plots	Manuscript and data citation
Kemp, K. B.	155	Kemp, K. B., P. E. Higuera, and P. Morgan. 2016. Fire legacies impact conifer regeneration across environmental gradients in the U.S. northern Rockies. Landscape Ecology 31:619-636. https://doi.org/10.1007/s10980-015-0268-3<br>Kemp, K. B., P. E. Higuera, P. Morgan, and J. T. Abatzoglou. 2019. Climate will increasingly determine post-fire tree regeneration success in low-elevation forests, Northern Rockies, USA. Ecosphere 10:e02568. https://doi.org/10.1002/ecs2.2568<br>Stevens-Rumann, C. S., K. B. Kemp, P. E. Higuera, B. J. Harvey, M. T. Rother, D. C. Donato, P. Morgan, and T. T. Veblen. 2018. Evidence for declining forest resilience to wildfires under climate change. Ecology Letters 21:243-252. https://doi.org/10.1111/ele.12889<br>**Data:** Kemp, Kerry B.; Higuera, Philip E.; Morgan, Penelope; Abatzoglou, John T. 2019. Data from: Climate will increasingly determine post-fire tree regeneration success in low-elevation forests, Northern Rockies, USA. Dryad, Dataset, https://doi.org/10.5061/dryad.9g91451
Kreider, M. R. & Yocom, L. L.	401	Kreider, M. R., and L. L. Yocom. 2021. Aspen seedling establishment, survival, and growth following a high-severity wildfire. Forest Ecology and Management 493:119248. https://doi.org/10.1016/j.foreco.2021.119248<br>Kreider, M. R., and L. L. Yocom. 2021. Low-density aspen seedling establishment is widespread following recent wildfires in the western United States. Ecology 102:e03436. https://doi.org/10.1002/ecy.3436
Kulakowski, D. & Gill, N. S.	26	Gill, N. S., F. Sangermano, B. Buma, and D. Kulakowski. 2017. Populus tremuloides seedling establishment: An underexplored vector for forest type conversion after multiple disturbances. Forest Ecology and Management 404:156-164. https://doi.org/10.1016/j.foreco.2017.08.008<br>Gill, N. S., D. Jarvis, J. Rogan, and D. Kulakowski. 2020. Disturbance history modulates how litter and herbaceous cover influence conifer regeneration after fire. International Journal of Wildland Fire 29:519-529. https://doi.org/10.1071/WF19028<br>Gill, N. S., D. Jarvis, T. T. Veblen, S. T. Pickett, and D. Kulakowski. 2017. Is initial post-disturbance regeneration indicative of longer-term trajectories? Ecosphere 8:e01924. https://doi.org/10.1002/ecs2.1924<br>**Data:** Gill, N., Buma, B., Kulakowski, D., Sangermano, F. 2018. Data for: Constraints on *Populus tremuloides* seedling establishment define an opportunity for forest type conversion after multiple disturbances. Mendeley Data, V1, https://doi.org/10.17632/69dmg7rnty.1
Littlefield, C. E.	51	Littlefield, C. E. 2019. Topography and post-fire climatic conditions shape spatio-temporal patterns of conifer establishment and growth. Fire Ecology 15:34. https://doi.org/10.1186/s42408-019-0047-7
Meigs, G. W.	47	Meigs, G. W., D. C. Donato, J. L. Campbell, J. G. Martin, and B. E. Law. 2009. Forest Fire Impacts on Carbon Uptake, Storage, and Emission: The Role of Burn Severity in the Eastern Cascades, Oregon. Ecosystems 12:1246-1267. https://doi.org/10.1007/s10021-009-9285-x<br>**Data:** Law, B.E., and L.T. Berner. 2015. NACP TERRA-PNW: Forest Plant Traits, NPP, Biomass, and Soil Properties, 1999-2014. ORNL DAAC, Oak Ridge, Tennessee, USA. http://dx.doi.org/10.3334/ORNLDAAC/1292
Ouzts, J.	41	Ouzts, J., T. Kolb, D. Huffman, and A. S. Meador. 2015. Post-fire ponderosa pine regeneration with and without planting in Arizona and New Mexico. Forest Ecology and Management 354:281-290. https://doi.org/10.1016/j.foreco.2015.06.001<br>**Data:** Rodman, K. C. et al. (2022), Data from: A changing climate is snuffing out post-fire recovery in montane forests, Dryad, Dataset, https://doi.org/10.5061/dryad.qz612jmb7

Contributor	Plots	Manuscript and data citation
Peeler, J. L.	69	Peeler, J. L. and E. A. H. Smithwick. 2020. Seed source pattern and terrain have scale-dependent effects on post-fire tree recovery. Landscape Ecology 35:1945-1959. https://doi.org/10.1007/s10980-020-01071-z<br>Peeler, J. L. and E. A. H. Smithwick. 2021. Interactions between landscape and local factors inform spatial action planning in post-fire forest environments. Landscape Ecology 36:3523-3537 https://doi.org/10.1007/s10980-021-01325-4
Povak, N.A. & Larson, A.J.	248	Povak, N. A., D. J. Churchill, C. A. Cansler, P. F. Hessburg, V. Kane, J. T. Kane, J. A. Lutz, and A. J. Larson. 2020. Wildfire severity and postfire salvage harvest effects on long-term forest regeneration. Ecosphere 11:e03199. https://doi.org/10.1002/ecs2.3199<br>**Data:** Povak, N. A., D. J. Churchill, C. A. Cansler, P. F. Hessburg, V. Kane, J. T. Kane, J. A. Lutz, and A. J. Larson. 2021. Data from "Wildfire severity and postfire salvage harvest effects on long-term forest regeneration". Fort Collins, CO: Forest Service Research Data Archive. https://doi.org/10.2737/RDS-2020-0079
Roccaforte, J. P.	217	Roccaforte, J. P., P. Z. Fulé, W. W. Chancellor, and D. C. Laughlin. 2012. Woody debris and tree regeneration dynamics following severe wildfires in Arizona ponderosa pine forests. Canadian Journal of Forest Research 42:593-604. https://doi.org/10.1139/x2012-010<br>Roccaforte, J. P., A. S. Meador, A. E. M. Waltz, M. L. Gaylord, M. T. Stoddard, and D. W. Huffman. 2018. Delayed tree mortality, bark beetle activity, and regeneration dynamics five years following the Wallow Fire, Arizona, USA: Assessing trajectories towards resiliency. Forest Ecology and Management 428:20-26. https://doi.org/10.1016/j.foreco.2018.06.012
Rodman, K. C.	547	Rodman, K. C., T. T. Veblen, M. A. Battaglia, M. E. Chambers, P. J. Fornwalt, Z. A. Holden, T. E. Kolb, J. R. Ouzts, and M. T. Rother. 2020. A changing climate is snuffing out post-fire recovery in montane forests. Global Ecology and Biogeography 29:2039-2051. https://doi.org/10.1111/geb.13174<br>Rodman, K. C., T. T. Veblen, T. B. Chapman, M. T. Rother, A. P. Wion, and M. D. Redmond. 2020. Limitations to recovery following wildfire in dry forests of southern Colorado and northern New Mexico, USA. Ecological Applications 30:e02001. https://doi.org/10.1002/eap.2001<br>**Data:** Rodman, K. C. et al. (2022), Data from: A changing climate is snuffing out post-fire recovery in montane forests, Dryad, Dataset, https://doi.org/10.5061/dryad.qz612jmb7
Rother, M. T.	291	Rother, M. T., and T. T. Veblen. 2016. Limited conifer regeneration following wildfires in dry ponderosa pine forests of the Colorado Front Range. Ecosphere 7:e01594. https://doi.org/10.1002/ecs2.1594<br>Stevens-Rumann, C. S., K. B. Kemp, P. E. Higuera, B. J. Harvey, M. T. Rother, D. C. Donato, P. Morgan, and T. T. Veblen. 2018. Evidence for declining forest resilience to wildfires under climate change. Ecology Letters 21:243-252. https://doi.org/10.1111/ele.12889<br>**Data:** Rodman, K. C. et al. (2022), Data from: A changing climate is snuffing out post-fire recovery in montane forests, Dryad, Dataset, https://doi.org/10.5061/dryad.qz612jmb7

Contributor	Plots	Manuscript and data citation
Safford, H. D.	2007	Shive, K. L., H. K. Preisler, K. R. Welch, H. D. Safford, R. J. Butz, K. L. O'Hara, and S. L. Stephens. 2018. From the stand scale to the landscape scale: predicting the spatial patterns of forest regeneration after disturbance. Ecological applications 28:1626-1639. https://doi.org/10.1002/eap.1756<br>Stewart, J.A.E., Mantgem, P.J. van, Young, D.J.N., Shive, K.L., Preisler, H.K., Das, A.J., Stephenson, N.L., Keeley, J.E., Safford, H.D., Wright, M.C., Welch, K.R., Thorne, J.H., 2021. Effects of postfire climate and seed availability on postfire conifer regeneration. Ecological Applications n/a, e2280. https://doi.org/10.1002/eap.2280<br>Welch, K. R., H. D. Safford, and T. P. Young. 2016. Predicting conifer establishment post wildfire in mixed conifer forests of the North American Mediterranean-climate zone. Ecosphere 7:e01609. https://doi.org/10.1002/ecs2.1609
Shive, K. L.	90	Shive, K. L., H. K. Preisler, K. R. Welch, H. D. Safford, R. J. Butz, K. L. O'Hara, and S. L. Stephens. 2018. From the stand scale to the landscape scale: predicting the spatial patterns of forest regeneration after disturbance. Ecological applications 28:1626-1639. https://doi.org/10.1002/eap.1756<br>Stewart, J.A.E., Mantgem, P.J. van, Young, D.J.N., Shive, K.L., Preisler, H.K., Das, A.J., Stephenson, N.L., Keeley, J.E., Safford, H.D., Wright, M.C., Welch, K.R., Thorne, J.H., 2021. Effects of postfire climate and seed availability on postfire conifer regeneration. Ecological Applications n/a, e2280. https://doi.org/10.1002/eap.2280
Singleton, M. P.	54	Singleton, M. P., A. E. Thode, A. J. S. Meador, and J. M. Iniguez. 2021. Moisture and vegetation cover limit ponderosa pine regeneration in high-severity burn patches in the southwestern US. Fire Ecology 17:14. doi.org/10.1186/s42408-021-00095-3
Stevens, J. T.	372	Stevens, J. T., H. D. Safford, and A. M. Latimer. 2014. Wildfire-contingent effects of fuel treatments can promote ecological resilience in seasonally dry conifer forests. Canadian Journal of Forest Research 44:843-854. doi.org/10.1139/cjfr-2013-0460<br>Barclay, A. D., J. L. Betancourt, and C. D. Allen. 2004. Effects of seeding ryegrass (*Lolium multiflorum*) on vegetation recovery following fire in a ponderosa pine (*Pinus ponderosa*) forest. International Journal of Wildland Fire 13:183-194. https://doi.org/10.1071/WF03012<br>**Related data:** Stevens, Jens T.; Safford, Hugh D.; Harrison, Susan; Latimer, Andrew M. (2016), Data from: Forest disturbance accelerates thermophilization of understory plant communities, Dryad, Dataset, https://doi.org/10.5061/dryad.q2n8p
Stevens-Rumann, C. S.	573	Stevens-Rumann, C. S., K. B. Kemp, P. E. Higuera, B. J. Harvey, M. T. Rother, D. C. Donato, P. Morgan, and T. T. Veblen. 2018. Evidence for declining forest resilience to wildfires under climate change. Ecology Letters 21:243-252. https://doi.org/10.1111/ele.12889<br>Schapira, Z., C. S. Stevens-Rumann, D. Shorrock, C. Hoffman, and A. Chambers. 2021. Beetlemania: Is the bark worse than the bite? Rocky Mountain subalpine forests recover differently after spruce beetle outbreaks and wildfires. Forest Ecology and Management 482:118879. https://doi.org/10.1016/j.foreco.2020.118879<br>Stevens-Rumann, C. S., and P. Morgan. 2016. Repeated wildfires alter forest recovery of mixed-conifer ecosystems. Ecological Applications 26:1842-1853. https://doi.org/10.1890/15-1521.1<br>Wooten J., C. S. Stevens-Rumann, M. Rocca M, Z. H. Schapira. 2022. Microenvironment characteristics and early regeneration after the 2018 Spring Creek Wildfire and post-fire logging in Colorado, USA. Fire Ecology 18:10. https://doi.org/10.1186/s42408-022-00133-8<br>Wooten J. 2021. The effects of postfire logging on microclimates and surface fuels. Masters of Science Thesis. Colorado State University.<br>Stevens-Rumann, C., and P. Fornwalt. 2018. Forest vulnerability to disturbances. Final Report to Boulder County Open Space, Small Grants Program.

Contributor	Plots	Manuscript and data citation
Swanson, D. J.	13	Data published as part of this manuscript: Davis, K. T. et al. 2023. Data from: Reduced fire severity offers near-term buffer to climate-driven declines in conifer resilience across the western United States. Dryad, Dataset, https://doi.org/10.5061/dryad.0rxwdbs47
Taylor, A.	71	Knapp, E., A. Taylor, M. Coppoletta, and N. Pawlikowski. 2019. Vegetation succession in an old-growth ponderosa pine forest following structural restoration with fire: implications for retreatment and maintenance. Joint Fire Science Program Report, Project 15-07-1-19. https://www.firescience.gov/projects/15-1-07-19/project/15-1-07-19_final_report.pdf
Tepley, A. J.	57	Tepley, A. J., J. R. Thompson, H. E. Epstein, and K. J. Anderson-Teixeira. 2017. Vulnerability to forest loss through altered postfire recovery dynamics in a warming climate in the Klamath Mountains. Global Change Biology 23:4117-4132. https://doi.org/10.1111/gcb.13704<br>Cinoğlu, D., H. E. Epstein, A. J. Tepley, K. J. Anderson-Teixeira, J. R. Thompson, and S. S. Perakis. 2021. Climatic aridity shapes post-fire interactions between *Ceanothus* spp. and Douglas-fir (*Pseudotsuga menziesii*) across the Klamath Mountains. Forests 12:1567. https://doi.org/10.3390/f12111567
Turner, M. G.	72	Turner, M. G., T. G. Whitby, D. B. Tinker, and W. H. Romme. 2016. Twenty-four years after the Yellowstone Fires: Are postfire lodgepole pine stands converging in structure and function? Ecology 97:1260-1273. https://doi.org/10.1890/15-1585.1<br>Turner, M. G., D. B. Tinker, W. H. Romme, D. M. Kashian and C. M. Litton. 2004. Landscape patterns of sapling density, leaf area, and aboveground net primary production in postfire lodgepole pine forests, Yellowstone National Park (USA). Ecosystems 7:751-775. https://doi.org/10.1007/s10021-004-0011-4<br>**Data:** Turner, Monica G.; Whitby, Timothy G.; Tinker, Daniel B.; Romme, William H. (2017), Data from: Twenty-four years after the Yellowstone fires: are postfire lodgepole pine stands converging in structure and function?, Dryad, Dataset, https://doi.org/10.5061/dryad.1pr7k
Urza, A. K.	73	Urza, A. K. and J. S. Sibold. 2017. Climate and seed availability initiate alternate post-fire trajectories in a lower subalpine forest. Journal of Vegetation Science 28:43-56. https://doi.org/10.1111/jvs.12465
Young, D. J. N.	194	Young, D. J. N., C. M. Werner, K. R. Welch, T. P. Young, H. D. Safford, and A. M. Latimer. 2019. Post-fire forest regeneration shows limited climate tracking and potential for drought-induced type conversion. Ecology 100:e02571. https://doi.org/10.1002/ecy.2571
